# Taurine metabolism is modulated in *Vibrio*-infected *Penaeus vannamei* to shape shrimp antibacterial response and survival

**DOI:** 10.1186/s40168-022-01414-9

**Published:** 2022-12-05

**Authors:** Zhongyan Wang, Jude Juventus Aweya, Defu Yao, Zhihong Zheng, Chuanqi Wang, Yongzhen Zhao, Shengkang Li, Yueling Zhang

**Affiliations:** 1 Institute of Marine Sciences and Guangdong Provincial Key Laboratory of Marine Biotechnology, College of Science, Shantou University, Shantou, 515063 Guangdong China; 2 College of Ocean Food and Biological Engineering, Fujian Provincial Key Laboratory of Food Microbiology and Enzyme Engineering, Jimei University, Xiamen, 361021 Fujian China; 3 Guangxi Academy of Fishery Sciences, Guangxi Key Laboratory of Aquatic Genetic Breeding and Healthy Aquaculture, Nanning, 530021 China; 4 Southern Marine Science and Engineering Guangdong Laboratory, Guangzhou, 511458 China

**Keywords:** Metabolites, Transcriptome, Taurine, Penaeid shrimp, Antibacterial, *Vibrio parahaemolyticus*

## Abstract

**Background:**

Numerous microorganisms are found in aquaculture ponds, including several pathogenic bacteria. Infection of cultured animals by these pathogens results in diseases and metabolic dysregulation. However, changes in the metabolic profiles that occur at different infection stages in the same ponds and how these metabolic changes can be modulated by exogenous metabolites in *Penaeus vannamei* remain unknown.

**Results:**

Here, we collected gastrointestinal tract (GIT) samples from healthy, diseased, and moribund *P. vannamei* in the same aquaculture pond for histological, metabolic, and transcriptome profiling. We found that diseased and moribund shrimp with empty GITs and atrophied hepatopancreas were mainly infected with *Vibrio parahaemolyticus* and *Vibrio harveyi*. Although significant dysregulation of crucial metabolites and their enzymes were observed in diseased and moribund shrimps, diseased shrimp expressed high levels of taurine and taurine metabolism-related enzymes, while moribund shrimp expressed high levels of hypoxanthine and related metabolism enzymes. Moreover, a strong negative correlation was observed between taurine levels and the relative abundance of *V. parahaemolyticus* and *V. harveyi*. Besides, exogenous taurine enhanced shrimp survival against *V. parahaemolyticus* challenge by increasing the expression of key taurine metabolism enzymes, mainly, cysteine dioxygenase (CDO) and cysteine sulfinic acid decarboxylase (CSD).

**Conclusions:**

Our study revealed that taurine metabolism could be modulated by exogenous supplementation to improve crustacean immune response against pathogenic microbes.

Video Abstract

**Supplementary Information:**

The online version contains supplementary material available at 10.1186/s40168-022-01414-9.

## Introduction

The Pacific white shrimp, *Penaeus vannamei*, is the most cultured species globally, but various infections and diseases have impacted its farming and resulted in huge economic losses in recent years [1–3]. Shrimp are poikilothermic; hence, drastic changes in the ecosystem increase their susceptibility to bacterial [4–6], viral [7–9], and parasitic [10, 11] infections. Moreover, shrimp possess only an innate immune system [12] that employs mechanisms such as phagocytosis [13], apoptosis [14], immune effector molecules (see recent review [15]), and metabolic reprogramming [16–18] to respond to pathogens.

Generally, substantial metabolic changes occur during pathogenic infections, which are either induced by the pathogens to optimize their survival in the host or as a host response to restrict the replication and spread of the pathogens [16, 19]. Metabolic changes resulting from host–pathogen interactions establish a close relationship between metabolic systems and the immune system [20]. Modulation of metabolic pathways, therefore, impacts the development and progression of infections or diseases. Indeed, most nutrients (i.e., lipids, organic acids, fatty acids, nucleic acids, vitamins, amino acids, etc.) and their metabolites serve both nutritional [21] and immune functions [22, 23]. For instance, intermediates or metabolites of glycolysis and the tricarboxylic acid (TCA) cycle also play crucial roles in cell signaling [24]. In *Klebsiella pneumoniae*-challenged mice, plasma levels of d-glucose, glutamine, l-serine, and inositol in the survival group increased compared with the dead group, while exogenous addition of l-serine, l-valine, and l-leucine enhanced bacteria phagocytosis to increase mouse survival [19]. Similarly, when zebrafish were challenged with *Vibrio alginolyticus*, the surviving group had significantly high levels of malic acid in their body fluids compared with healthy and moribund groups [25]. In white spot syndrome virus (WSSV)-infected penaeid shrimp (*P. vannamei*), intestinal levels of linoleic acid increased significantly, resulting in the activation of the extracellular signal-regulated kinase (ERK)/nuclear factor kappa-light-chain-enhancer of activated B cells (NF-κB) signaling pathway to induce the expression of antibacterial peptides and *Vago5* (an IFN-like gene), thereby inhibiting viral proliferation and enhancing shrimp survival [16]. Thus, through metabolic reprogramming, the host immune response against pathogens could be enhanced [26].

During host–pathogen interactions, some pathogens directly or indirectly skew host metabolism in their favor. For instance, in the Gram-negative bacterium *Pseudomonas aeruginosa*, the virulence factor and quorum sensing signaling molecule, pyocyanin, modulates some physiological processes in human cells, such as lysosomal dysfunction and protease release, to induce apoptosis and impair host defenses, thus allowing the bacterium to proliferate [27]. Similarly, during infection of mammalian cells, a secondary metabolite, gliotoxin (GT), produced by the saprophytic fungus *Aspergillus fumigatus*, enables the fungus to induce reactive oxygen species (ROS) production by activating Bak (Bcl-2 family member) to promote apoptosis and cause high morbidity and mortality [28]. In aquatic animals, during infection of Chinese perch (*Siniperca chuatsi*) with infectious spleen and kidney necrosis virus (ISKNV), the virus metabolizes high levels of glucose for replication in the early infection stages, but switches to the use of more glutamine (for lipid synthesis) in the later stages to ensure viral maturation [29]. Similarly, in *Vibrio parahaemolyticus*-challenged mud crab (*Scylla paramamosain*), moribund crabs had elevated plasma levels of saturated fatty acids (e.g., myristic acid, palmitic acid, stearic acid, etc.) but low levels of unsaturated fatty acids (e.g., arachidonic and eicosapentaenoic acids) and amino acids (e.g., hydroxyproline, arginino-succinic acid, and malate) compared with the survival group, suggesting that the bacteria attenuate host energy biosynthesis and decrease levels of metabolites with immune-related functions to impair host bacterial clearance [30]. When infected by the strain of *V. parahaemolyticus* that causes acute hepatopancreatic necrosis disease (AHPND), increased levels of bile acids, pVA plasmid, and Pir toxin were found in the stomach of shrimp (*Penaeus vannamei*), suggesting that bile acid modulates the bacterium’s virulence [31]. The Warburg effect and glutamine metabolism are also triggered in WSSV-infected *P. vannamei*, indicating that both oxidative and reductive glutamine metabolic pathways are activated to promote viral replication [32].

Various amino acids and their metabolites have been implicated in immunomodulation in mammals, while few studies have thus far explored the role of amino acids and/or their metabolites in the immune regulation of crustaceans [33]. Among the amino acids that play key physiological functions in mammals, taurine, the most abundant free nonproteinogenic amino acid [34, 35], has been implicated in various developmental and biological functions [36]. For instance, taurine and its metabolites play vital roles in mammalian immune response [37, 38], by inducing the release of IL-35 [39], inhibiting oxidative stress [40], regulating the release of pro-inflammatory cytokines [41], and modulating inflammatory responses [42, 43], through the AMPK-mTOR [44] or TLRs/NF-κB [45] pathways. However, in crustaceans, the role of endogenous taurine is unclear, although dietary taurine supplementation has been shown to modulate several physiological processes, including immune response, antioxidant capacity, inflammation, etc. in various marine organisms, such as juvenile yellow catfish *Pelteobagrus fulvidraco* [46, 47], zebrafish *Danio rerio* [48], Chinese mitten crab *Eriocheir sinensi* [49], European seabass *Dicentrarchus labrax* [50], and *Carassius carassius* [51].

Here, we report that, in an aquaculture pond containing Pacific white shrimp (*P. vannamei*) of different pathological stages, most diseased shrimp were infected with *Vibrio* (mainly *V. parahaemolyticus* and *V. harveyi*) and key metabolites and their corresponding enzymes were dysregulated. Notably, the gastrointestinal tract (GIT) of diseased surviving shrimp expressed high levels of taurine and its related metabolism enzymes, whereas diseased moribund shrimp expressed low taurine but high levels of hypoxanthine and its related metabolism enzymes. Most importantly, exogenous taurine supplementation enhanced shrimp survival against *V. parahaemolyticus* challenge.

## Materials and methods

### Experimental animals, sample preparation, and pathogen challenge

Healthy (control), diseased (survival), and moribund adult shrimp (*Penaeus vannamei*) with a mean weight of 7 ± 0.5 g were obtained from Haosheng aquaculture Co., Ltd., Shantou, Guangdong, China (23.28 °N, 116.69 °E). Tissues (i.e., hemolymph, hepatopancreas, stomach, and intestine) were collected from each group of shrimps (*n*=26) as previously described [52]. Samples for DNA, RNA, and protein determination were snap-frozen in liquid nitrogen before being stored at −80 °C, while samples for metabolomics analysis were preserved in cold methanol. The samples used for bacterial determination with thiosulfate-citrate-bile salts-sucrose (TCBS) agar were stored on ice, while those for histological examination were directly placed into 4% paraformaldehyde for fixation.

Healthy *P. vannamei* (mean weight 5–8 g) were also purchased from Huaxun Aquatic Product Corporation, Shantou, Guangdong, China (23.36 °N, 116.66 °E) for pathogen challenge experiments. Shrimp were cultured in laboratory tanks (containing artificial seawater of salinity 10 ppm and temperature 23–26 °C) for 2 to 3 days and fed twice daily with commercial feed (34–37% protein). In the challenge and survival experiments, shrimp were injected with 100 μL of taurine (1.25, 2.50, and 5.00 mg/mL) or control (solvent vehicle) for 24 h before being challenged with 5 × 10^5^ CFU/shrimp of *V. parahaemolyticus* (isolate PD-2), the strain that causes acute hepatopancreatic necrosis disease (AHPND). Shrimp used for the transcriptome analysis were injected with taurine (2.50 mg/mL) for 24 h followed by a challenge with 5 × 10^5^ CFU/shrimp of *V. parahaemolyticus* (PD-2) or an equal volume of sterile saline (0.65%). Hepatopancreas samples were collected from five randomly selected shrimp from each group at 24 h post-challenge for total RNA extraction.

The bacteria used, i.e., *Vibrio harveyi* (MCCC1H00031, GenBank: X74706.1) and *Vibrio parahaemolyticus* (MCCC1H00057, GenBank: FJ161313.1), were purchased from the Marine Culture Collection of China (MCCC), while *V. parahaemolyticus* (PD-2) was a kind gift from Professor Lo Chufang (National Cheng Kung University, Taiwan, China) [53]. On the other hand, *Streptococcus iniae* (GenBank: NZ_JH930418.1) and *Escherichia coli* (K-12) (GenBank: NC_000913.3) were previously isolated from penaeid shrimp by our group. Bacteria were cultured at 37 ° C in Tryptic Soy Broth (TSB) medium (17 g peptone, 3 g soybean peptone, 2.5 g glucose, 30 g sodium chloride, 2.5 g dipotassium hydrogen phosphate, pH 7.2) for 24 h. before being diluted to the required concentration before use. In China, because no specific ethical approval is required for carrying out experimental work with shrimp or crustaceans (Regulations of Guangdong Province on the Administration of Experimental Animals http://www.gd.gov.cn/zwgk/wjk/zcfgk/content/post_2524545.html and Regulations of the People’s Republic of China on the Administration of Experimental Animals https://kyc.jnmc.edu.cn/2021/0826/c2735a122933/page.htm), all animal experiments were conducted in accordance with these guidelines and the Animal Research and Ethics Committee of Shantou University, Guangdong, China.

### Genomic DNA and total RNA extraction

Genomic DNA (gDNA) was extracted from shrimp hepatopancreas samples using the genomic DNA extraction kit for marine animal tissues (TIANGEN, Beijing, China) following the manufacturer’s protocol. The gDNA concentration was determined using a NanoDrop 2000 spectrophotometer (Thermo Fisher Scientific, Wilmington, MA, USA), and the quality and integrity were validated with an Agilent 2100 Bioanalyzer (Agilent Technologies, Santa Clara, CA, USA) and also using 1% agarose gel electrophoresis.

Total RNA was also extracted from hepatopancreas using TRIzol Plus RNA Purification Kit (Invitrogen, Carlsbad, CA) according to the manufacturer’s instructions. The total RNA concentration was measured using a NanoDrop 2000 spectrophotometer (Thermo Fisher Scientific, Wilmington, MA, USA) followed by determining the RNA Integrity Number (RIN) using an Agilent 2100 Bioanalyzer (Agilent Technologies, Santa Clara, CA, USA). The quality of the total RNA was further ascertained using the 260/280 ratio (>1.9) and also analyzed on 1% agarose gel electrophoresis. Only high-quality samples were used for downstream analyses.

### Bacteria types and abundance determination using PCR

To identify bacteria types and abundance in shrimp tissue samples, quantitative PCR-based methods were used. First, standard curves were prepared using different bacteria strains and gene-specific primers (Table S1) as previously described [54–56] followed by qPCR analysis with 5 μL of 2 × RealStar Green Power Mixture (Genstar, Beijing, China), 0.5 μL(10 μM) each of the forward and reverse gene-specific primers (Table S1), 1 μL (50 ng/μL) gDNA, and 3 μL ddH_2_O. The qPCR reaction was carried out on the qTOWER 3 G RT-PCR system (Analytik Jena, Jena, Germany) using the following cycling conditions: one cycle at 95 °C for 10 min, 45 cycles at 95 °C for 15 s, and 60 °C for 30 s. For the PCR-based bacteria identification, gene-specific primers for the various bacteria (Supplemental Table S1) were used with gDNA (20 ng/reaction) at the following conditions for each gene: 16S rRNA (one cycle at 96 °C for 3 min; 28 cycles of 95 °C for 30 s, 55 °C for 30 s, and 72 °C for 30 s; and one cycle at 72 °C for 10 min); *Vibrio* (one cycle at 96 °C for 3 min; 35 cycles of 95 °C for 30 s, 55 °C for 30 s, and 72 °C for 30 s; one cycle at 72 °C for 5 min); *V. harveyi* (one cycle at 96 °C for 3 min; 35 cycles of 95 °C for 30 s, 55 °C for 30 s, and 72 °C for 30 s; one cycle at 72 °C for 5 min); *pirB* (one cycle at 96 °C for 3 min; 35 cycles of 95 °C for 30 s, 55 °C for 30 s, and 72 °C for 30 s; one cycle at 72 °C for 5 min); AP4 (first PCR: one cycle at 96 °C for 6 min; 33 cycles of 95 °C for 30 s, 55 °C for 30 s, and 72 °C for 90 s; one cycle at 72 °C for 10 min. Second PCR: one cycle at 95 °C for 3 min; 33 cycles of 95°C for 30 s, 55 °C for 30 s, and 72 °C for 20 s; one cycle at 72 °C for 10 min) [57]; and *tlh* (one cycle at 95 °C for 3 min; 32 cycles of 95 °C for 30 s, 60 °C for 30 s, and 72 °C for 35 s; one cycle at 72 °C for 5 min) [58]. Bacteria used as control were directly subjected to PCR without extracting DNA.

### Sample preparation, histological examination, and metabolomics profiling

Histological examination of hepatopancreas samples from healthy, diseased, and moribund shrimp was processed and observed after H&E staining. First, the hepatopancreas samples were fixed with 4% paraformaldehyde for 48 h before being sliced and stained with hematoxylin and eosin (H&E) as previously described [59]. Samples were then observed and imaged with a Pannoramic MIDI light microscope (3DHISTECH, Budapest, Hungary).

To profile shrimp hepatopancreas metabolites, samples were prepared as previously described [25, 60] with some modifications. Briefly, 30 mg hepatopancreas were placed in 1000 μL ice-cold chromatographic grade (Sigma-Aldrich, St. Louis, MO, USA) before being lysed for 6 min with an ultrasonic cell disruptor (Xinyi 650E, Ningbo, China) and then centrifuged at 12000 rpm (10 min at 4 °C) to collect the supernatant. As an internal standard, 10 μL of 0.1 mg/mL ribitol (Sigma-Aldrich, St. Louis, MO, USA) was added. After samples were concentrated and dried in a rotary vacuum centrifuge (Labconco Corporation, Kansas, MO, USA), they were analyzed by gas chromatography coupled with mass spectrometry (GC-MS) as previously reported [61, 62]. Briefly, samples were first methoxylated and derivatized to protect the carbonyl moieties by incubating samples at 37 °C for 90 min on a shaker (200 rpm/min) with 80 μL of 20 mg/mL methoxyamine hydrochloride (Sigma-Aldrich, St. Louis, MO, USA) in pyridine. Next, 80 μL of N-methyl-N-trimethylsilyltri-fluoroacetamide (Sigma-Aldrich, St. Louis, MO, USA) was added before being incubated at 37 °C for 30 min on a shaker to obtain the derivatized of acidic protons. The derivatized samples were then analyzed by GC-MS by split-less injection of samples into a 30 m × 250 μm i.d. × 0.25 μm DBS-MS column and detected by Agilent 5975C VL MSD detector (Agilent Technologies, Palo Alto, USA). The initial temperature of the GC oven was maintained at 85 °C for 5 min, before being increased to 270 °C at a rate of 15 °C/min, and maintained for another 5 min. The carrier gas (helium) was maintained at a constant flow rate of 1 mL/min and the MS data was obtained in full scan mode at an operating range of 50–600 m/z. Each needle was run for 55 min, with a starting delay of 5 min, and electron impact ionization was applied to 70 eV at a scanning rate of 2 scan/s. Duplicate samples were analyzed and repeated for two biological samples. All of the metabolomic raw data were deposited to MetaboLights (http://www.ebi.ac.uk/metabolights/) [63]. The unique identifier is MTBLS4770, which can be found through the link www.ebi.ac.uk/metabolights/MTBLS4770.

The raw GC-MS data was analyzed using the automated mass spectral deconvolution and identification system software (AMDIS, version 2.62) followed by searching in the National Institute of Standards and Technology (NIST 08) mass spectrometry library to identify metabolites. After normalizing metabolite abundance with the internal standard (ribitol) to obtain a single matrix data [64], data were analyzed using in silico tools before being log converted, normalized data, and used to perform hierarchical clustering and heat map analysis on R (version 3.6.1). Principal component analysis (PCA) was performed on the normalized data using partial least squares discriminant analysis (PLS-DA), while S-plot analysis was performed on the normalized data using orthogonal partial least squares discriminant analysis (OPLS-DA) in SIMCA 14 software (Umetrics, Umea, Sweden). The data were also searched in the Kyoto Encyclopedia of Genes and Genomes (KEGG) (http://www.genome.jp/kegg/) database to obtain the pathways that the identified metabolites are enriched.

### ELISA

Shrimp hepatopancreas lysates were prepared as previously described [65] and used for enzyme-linked immunosorbent assay (ELISA) to validate the GS-MS data. Briefly, hepatopancreas samples were gently minced in 1 mL of 0.01 M PBS (pH 7.2) before being strained through a 150-mm steel mesh and centrifuged at 200 g (4°C for 10 min) to collect the cells. After being washed three times with PBS, cells were lysed at 4°C for 20 min with cell lysis buffer (25 mM Tris-HCl [pH 7.4], 1 mM EDTA, 150 mM NaCl, 1% NP-40, 5% glycerol) and centrifuged at 20,000 g (4°C for 10 min) to collect the supernatant, which was stored in aliquots at −80°C for later use. Next, the levels of taurine, uracil, and hypoxanthine were determined using commercial ELISA kits (Yajikit, Shanghai, China) following the manufacturer’s protocols. Briefly, standard stock solutions of taurine (400 pg/mL), uracil (240 ng/L), and hypoxanthine (400 ng/L) were diluted as follows: taurine (200 pg/mL, 100 pg/mL, 50 pg/mL, 25 pg/mL, and 12.5 pg/mL), uracil (120 ng/L, 60 ng/L, 30 ng/L, 15 ng/L, and 7.5 ng/L), and hypoxanthine (20 ng/L, 10 ng/L, 5 ng/L, 2.5 ng/L, and 1.25 ng/L). Next, 50 μL diluted standard or sample was placed into antibody pre-coated ELISA plates before being mixed gently and incubated at 37°C for 30 min. After extensive washing, 50 μL HRP-conjugate reagent was added and incubated at 37°C for 30 min. After washing 5 times, 50 μL chromogen solution A and 50 μL chromogen solution B were added in succession, mixed gently by shaking, and incubated in the dark at 37°C for 10 min. Finally, 50 μL stop solution was added and the optical density (OD) was measured on a microplate reader (Synergy H1, BioTek, Winooski, VT, USA) at 450 nm within 15 min.

Proline and glycerol levels were assayed using different commercial ELISA kits (Nanjing Jiancheng Bioengineering Institute, Nanjing, China) according to the manufacturer’s protocols. For proline, a standard curve was first prepared by diluting the proline standard (100 μg/mL) with reagent 1 into the following concentrations: 1μg/mL, 2μg/mL, 4μg/mL, 8μg/mL, and 16μg/mL. The ODs of these dilutions were then measured and a standard curve was plotted (*R*^2^=0.9996). Next, the assay was validated by determining the limit of detection (LOD) or limit of quantification (LOQ). To do this, the ODs of 12 blank samples were determined and the LOD was taken as OD greater than 0.043. Next, one of the test samples was diluted 10, 50, and 100 times, followed by measuring the ODs, and the concentration of proline was then extrapolated from the standard curves, which was within 1–9 μg/mL at the highest dilution (100 times). Based on this LOQ, all samples were diluted 100 times before testing. To determine the proline content in the test samples, 50 μL reagent 1 (blank), 50μL standard solution (proline 5 μg/mL), and 50 μL hepatopancreas cell lysates (diluted 100-fold) were added into 96-well plates, followed by the addition of 100 μL buffer and 100 μL chromogenic solution. Samples were incubated in a water bath at 100°C for 30 min, and after cooling with running water, the OD at 520 nm was measured. The ODs of the samples were then extrapolated to find the corresponding proline concentrations in the samples from the standard curve. For the determination of glycerol, the stock glycerol standard (4 mM) was first diluted (250 μmol/L, 125 μmol/L, 62.5 μmol/L, 31.25 μmol/L, 15.625 μmol/L, 7.8125 μmol/L). Next, reagent R1 and reagent R2 were mixed at a ratio of 4:1 to form the working solution, after which 10 μL distilled water (blank), 10 μL diluted standard solution, and 10 μL hepatopancreas cell lysates were added to 96-well plates, followed by the addition of 190 μL working solution. After being incubated at 37°C for 10 min, the OD at 550 nm was measured. The protein concentration of cell lysates was also measured using the Micro BCA protein assay kit (Nanjing Jiancheng Bioengineering Institute) according to the manufacturer’s instructions.

### Sample preparation and transcriptomic analysis

In the shrimp hepatopancreas transcriptome analysis, three cDNA libraries (designated control, PD-2, and taurine+PD-2) were constructed using high-quality total RNA samples (see the section “Genomic DNA and total RNA extraction”). First, mRNA was enriched from the total RNA samples using Oligo (dT) beads that base pair (A-T) with the poly-A before being fragmented randomly (into 100–400-bp fragments) with an ultra-sonicator and reverse transcribed into first-strand cDNA using the MGIEasy RNA Directional Library Prep Set kit (MGI Tech Co., Ltd., Shenzhen, Guangdong, China, Cat #1000006385). The cDNA samples were diluted to 200 ng/μL and three samples per treatment group were pooled together before being sent to a commercial company (BGI, Shenzhen, Guangdong, China), where sequencing adapters are added to the short cDNA fragments followed by paired-end RNA sequencing on the Illumina platform (Illumina Hiseq 4000). The assembled sequencing data has been submitted to GenBank under accession number PRJNA813696.

The obtained raw reads were filtered to remove adaptors, low-quality reads (more than 20% Q≤10 bases), and sequences with unknown nucleotides greater than 5%, followed by comparing the clean reads with the reference genome (https://www.ncbi.nlm.nih.gov/genome/10710) using the Trinity software release-20130225 [66]. After obtaining unigenes from transcript predictions, the fragments per kb per million reads (FPKM) was used as the unit of quantification [67], and the expression levels of genes and transcripts were quantified by normalized FPKM. The false discovery rate (FDR) control method was used to ensure the high quality of differentially expressed genes (DEGs). A threshold of unigenes with FDR <0.05 and |log2Ratio|≥1 was used to identify the DEGs [68]. Correlation analysis between samples and principal component analysis (PCA) were performed, while DEG clustering was analyzed using TBtools software version 1.089 [69]. Gene ontology (GO) functional classification was performed on all DEGs using the web gene ontology (WEGO v2.0) software (http://wego.genomics.org.cn/cgi-bin/wego/index.pl) [70]. The biological functions of unigenes were analyzed by the online KEGG server (KAAS) (http://www.genome.jp/kegg/kaas/) [71].

### Statistical analysis

Data are expressed as the mean ± standard error of the mean (SEM) unless otherwise stated. Statistical analyses were performed on the SPSS software (version 20) using Duncan’s multiple range test, one-way ANOVA, or Tamhane’s T2 test with significance considered at *p* < 0.05. Survival curves were analyzed by the Kaplan–Meier estimate, while Pearson correlation and linear regression were performed in GraphPad Prism 8.

## Results

### Gross signs, histopathological features, and gastrointestinal tract bacteria content

**Fig. 1:**
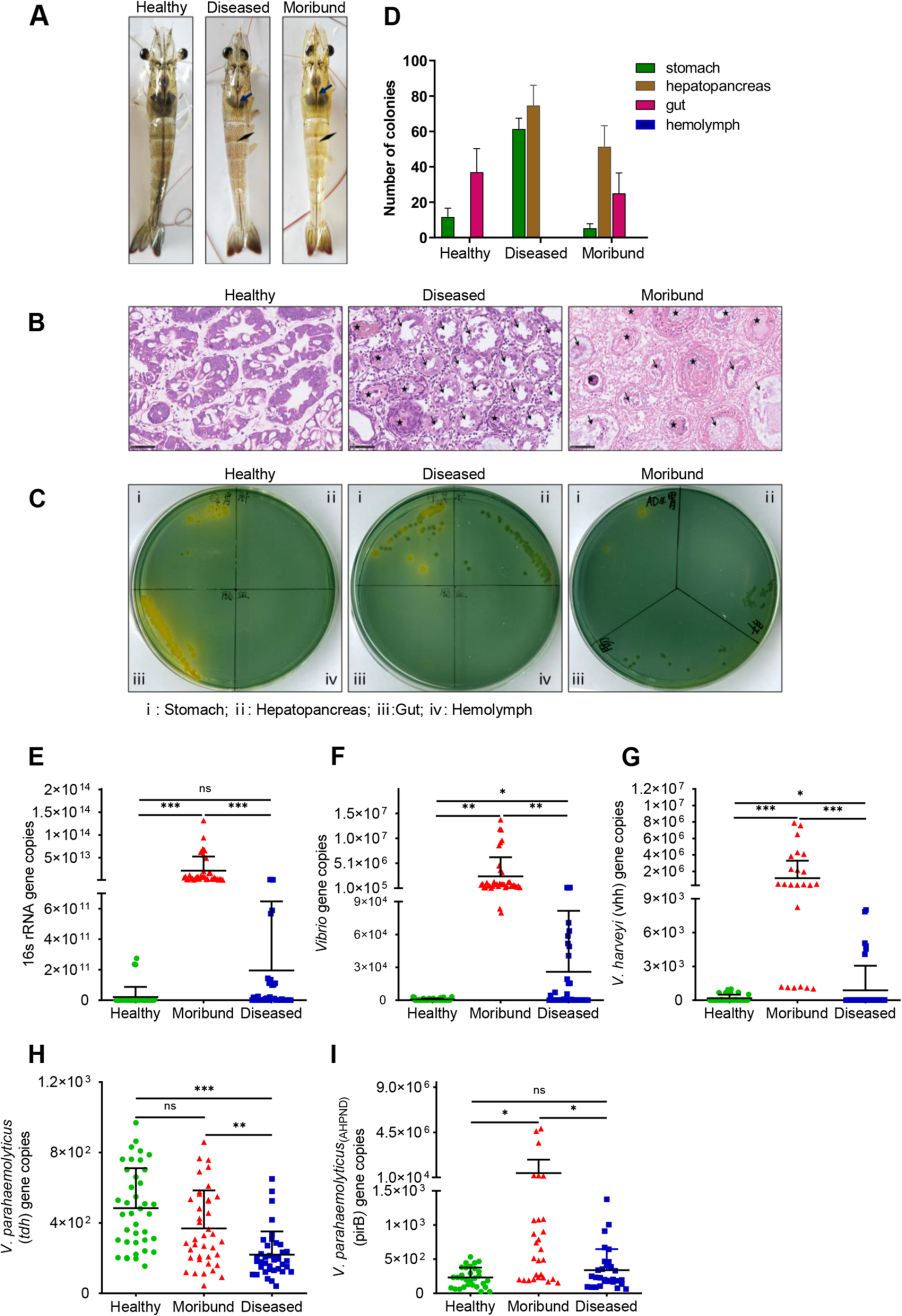
Pathological features of healthy, diseased, and moribund *Penaeus vannamei.* **A** Gross signs. **B** Cross-sectional histological micrographs of hepatopancreas after hematoxylin–eosin (HE) staining. Scale bar: 50μm. **C** Growth of *Vibrio* on thiosulfate–citrate–bile salts–sucrose (TCBS) agar selective culture plates. Green colonies: *Vibrio parahaemolyticus* and *Vibrio fluvialis*; yellow colonies: other *Vibrio* strains (i.e., *Vibrio alginolyticus*, *Vibrio cholerae*, *Vibrio harveyi*, and *Vibrio anguillarum*). **D** Quantified bacteria colonies (*n*=3). PCR-based quantification of the 16S rRNA gene in shrimp hepatopancreas. **E** Total bacterial abundance, **F** *Vibrio* abundance, **G** *V. harveyi* (*vhh*), **H** *V. parahaemolyticus* (*tdh*), and **I** *V. parahaemolyticus* (*pirB*). Results were reported as mean ± S.E.M (*n* = 3). ns, not significant, *p<0.05, **p < 0.01, ***p < 0.001

Gross examination of healthy and diseased penaeid shrimp (*Penaeus vannamei*) revealed that healthy shrimp had full and brown gastrointestinal tracts or GITs (stomachs, hepatopancreas, and intestine), whereas diseased and moribund shrimp had empty and pale GITs (Fig. 1A). Histopathological examination of hepatopancreas sections revealed exfoliation of hepatopancreatic tubular epithelial cells (arrow) and hemocyte infiltration (asterisk) in diseased and moribund shrimp but not in healthy shrimp (Fig. 1B). Moreover, the hepatopancreatic tubules of moribund shrimp were damaged, a characteristic feature of *Vibrio* infection [59]. To ascertain these results, GIT samples (stomach, hepatopancreas, and intestine) and hemolymph were cultured on TCBS selective medium. As shown in Fig. 1C and D, *Vibrio* were found in the intestine and stomach samples, but not in the hemolymph and hepatopancreas of healthy shrimp. In diseased shrimp, *Vibrio* were not found in the intestine and hemolymph, but in the stomach and hepatopancreas. On the other hand, the stomach, intestine, and hepatopancreas of moribund shrimp all had *Vibrio*, while hemolymph could not be withdrawn from moribund shrimp.

The rest of the experiments used hepatopancreas samples because it contains *Vibrio* in both diseased and moribund shrimp. To identify the *Vibrio* strains, PCR-based methods were used. Moribund shrimp had high total bacteria abundance compared with healthy (*p<0.001*) and diseased shrimp (*p<0.001*) (Fig. 1E and Fig. S1A). The absolute abundance of *Vibrio* was significantly high in diseased (*p<0.05*) and moribund (*p<0.01*) shrimp compared with healthy shrimp (Fig. 1F and Fig. S1B). Next, the main pathogenic *Vibrio* strains of penaeid shrimp (i.e., *V. parahaemolyticus*_(AHPND)_: the strain that causes acute hepatopancreatic necrosis disease (AHPND), *V. parahaemolyticus*, and *V. harveyi*) were screened and their relative abundance in hepatopancreas samples was determined using gene-specific primers. In both healthy and diseased shrimp, copies of the *vhh* gene (specific to *V. harveyi*) were found (Fig. 1G and Fig. S1C), while the *tdh* and *tlh* genes specific to *V. parahaemolyticus* were found in both healthy and diseased shrimp (Fig. 1H and Fig. S1D). On the other hand, copies of the *pirB* and *AP4* genes, which are specific to *V. parahaemolyticus*_(AHPND)_, were mainly found in moribund shrimp (Fig. 1I, Fig. S1E and S1F).

### Healthy and diseased shrimp display different global metabolic profiles

**Fig. 2:**
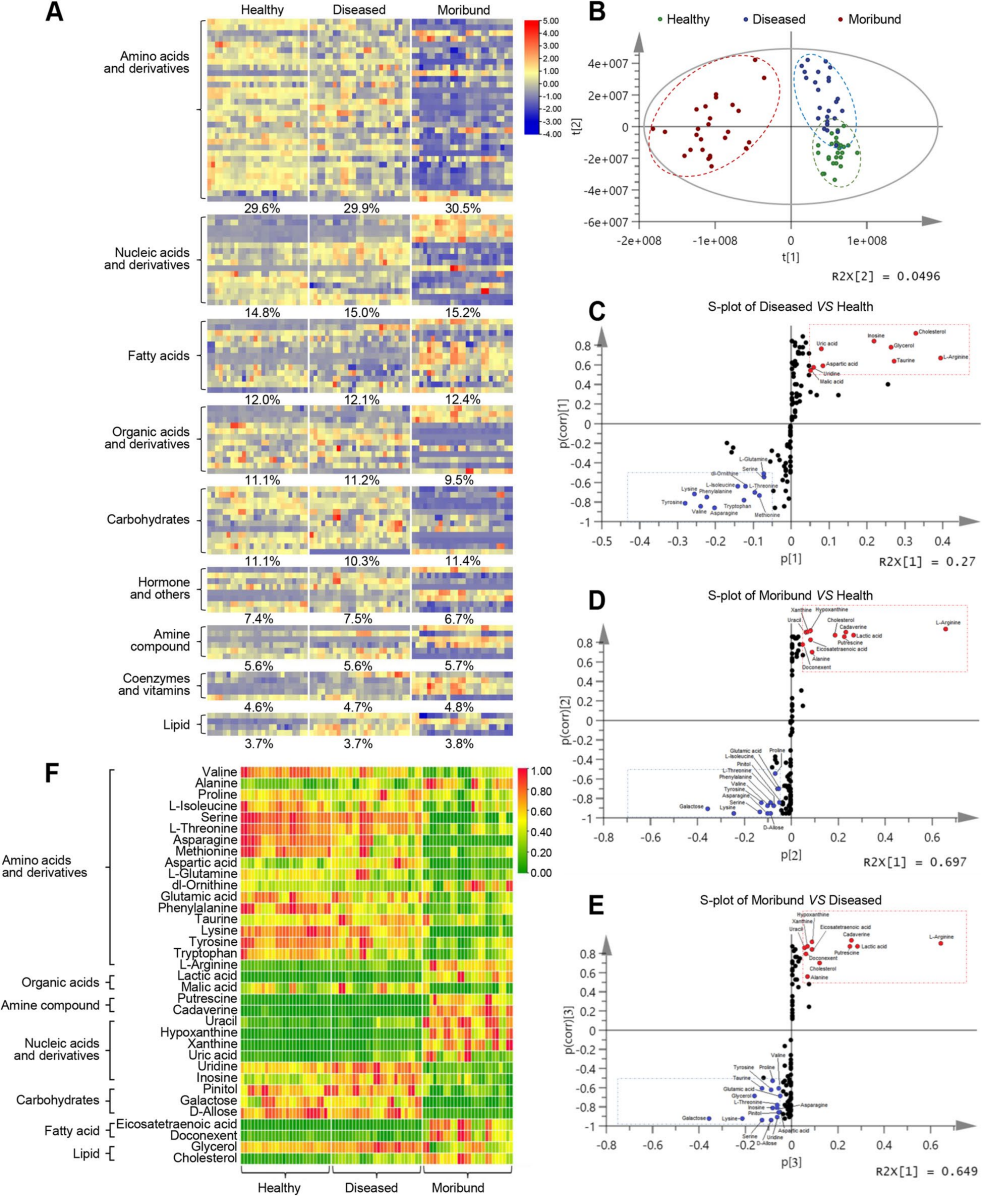
Global metabolic profiles of healthy, diseased, and moribund shrimps. **A** Metabolite categories analyzed by heatmap (*n*=26). **B** Principal component analysis (PCA) based on Bray–Curtis distance. **C** S-plot generated from OPLS-DA for diseased vs healthy shrimp. Dots represent individual metabolites (red: upregulated and blue: downregulated metabolites in disease shrimp). **D** S-plot generated from OPLS-DA for moribund vs healthy shrimp. Dots represent individual metabolites (red: upregulated and blue: downregulated metabolites in moribund shrimp). **E** S-plot generated from OPLS-DA for moribund vs diseased shrimp. Dots represent individual metabolites (red: upregulated and blue: downregulated metabolites in moribund shrimp). Covariance *p≥0.05* and correlation *p (corr)≥0.5.* **F** Significantly expressed metabolite categories related to survival identified from the S-plots and analyzed by heatmap (*n*=26)

Untargeted metabolomics analysis was used to profile metabolite changes in the hepatopancreas of healthy, diseased, and moribund shrimp. In healthy, diseased, and moribund shrimp, 108, 107, and 106 metabolites, respectively, were identified (Table S2). All the identified metabolites have similar biological functions and were mainly grouped into the following metabolites categories (Fig. 2A) in descending order: amino acids and derivatives (29–31%), nucleic acids and derivatives (14–16%), fatty acids (12–13%), organic acids and derivatives (9–12%), carbohydrates (10–12%), hormones and others (6–8%), amine compounds (5–6%), coenzymes and vitamins (4–5%), and lipids (3–4%).

Principal component analysis (PCA) and partial least-squares discrimination analysis (PLS-DA) revealed that the metabolites in the three groups of shrimps clustered separately (Fig. 2B) with no marked differences within groups. Next, we screened for metabolites related to survival using orthogonal partial least-squares discrimination analysis (OPLS-DA) and S-plot models. Based on an absolute covariance *p *≥ 0.05 and a correlation *p* ≥ 0.5 as a cutoff, three S-plots (Fig. 2C–E) were constructed and used to identify key metabolites or biomarkers in the three groups. When diseased and healthy shrimp were compared, 21 differential metabolites were identified, with 9 upregulated and 12 downregulated (Fig. 2C and Fig. S2A). Similarly, when moribund and healthy shrimp were compared, 24 differential metabolites were identified, 11 of which were upregulated and 13 downregulated (Fig. 2D and Fig. S2B). On the other hand, when moribund and diseased shrimp were compared, 27 differential metabolites were identified, including 11 upregulated and 16 downregulated (Fig. 2E and Fig. S2C). When the significantly dysregulated metabolites (Fig. S2A–S2C) were analyzed in terms of metabolite types (Fig. 2F), seven metabolite categories were obtained (Fig. S2D). Amino acids and derivatives constituted the most (51.43%), followed by nucleic acids and derivatives (17.14%), with the least being organic acids, amine compound, fatty acid, and lipid (5.71%). Further analysis of the metabolic pathways involved in the significantly altered metabolites using MetaboAnalyst, a pathway analysis tool [72], revealed that the top 25 enriched pathways were mainly enriched in 9 categories, i.e., translation, metabolism of other amino acids, amino acid metabolism, carbohydrate metabolism, energy metabolism, lipid metabolism, metabolism of cofactors and vitamins, metabolism of terpenoids and polyketides, and nucleotide metabolism (Fig. S2E).

### Significantly altered metabolites essential for shrimp survival

**Fig. 3:**
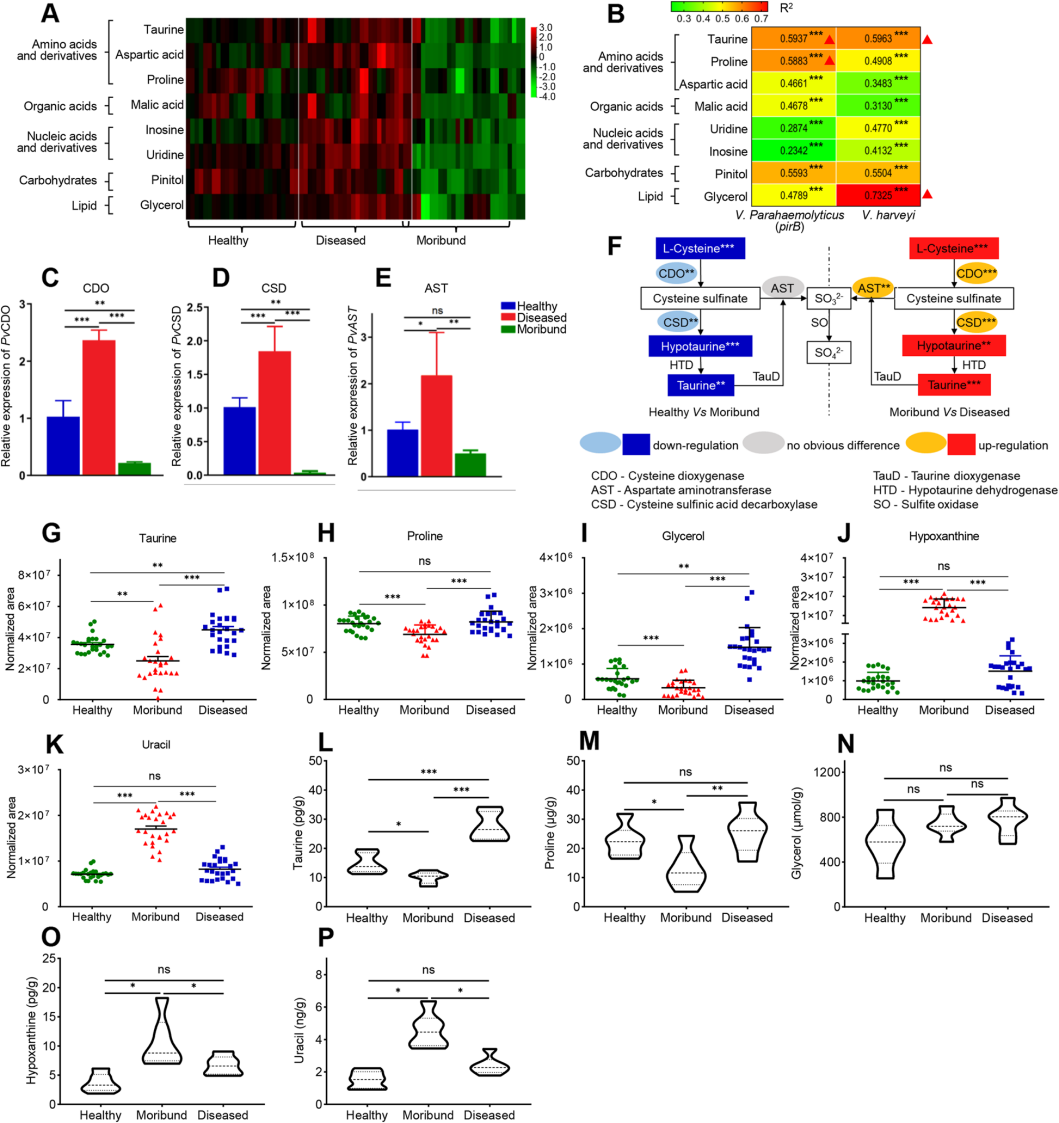
Changes in specific metabolites at different pathological stages of shrimp. **A** Immune-related metabolites analyzed by heatmap (*n*=26). **B** Correlation between different metabolites and *V. parahaemolyticus* (*pirB*) or *V. harveyi* (*vhh*) analyzed by heatmap. Relative **C** *CDO* gene, **D** *CSD* gene, and **E** *AST* gene expression in shrimp hepatopancreas. Results were reported as mean ±S.E.M (*n* = 3). *p < 0.05; **p < 0.01; ***p < 0.001. **F** Schematic diagram showing changes in taurine metabolism-related enzymes and metabolites at different pathologic stages in shrimp (*P. vannamei*). Scatter plots showing the differential expression of **G** taurine, **H** proline, **I** glycerol, **J** hypoxanthine, and **K** uracil determined by GC-MS analysis. Each dot represents an independent sample (*n*=26). *p < 0.05; **p < 0.01; ***p < 0.001. Expression levels of **L** taurine, **M** proline, **N** glycerol, **O** hypoxanthine, and **P** uracil determined by ELISA. Results were reported as mean ± S.E.M (*n* = 6). *p < 0.05; **p < 0.01; ***p < 0.001

To screen for metabolites that are crucial for shrimp survival, altered metabolites that were significantly downregulated in moribund shrimp compared with healthy shrimp and/or healthy shrimp compared with diseased shrimp were selected. Eight significantly altered metabolites were identified (Fig. 3A). When the correlation between these 8 metabolites and bacteria (*V. parahaemolyticus*_(AHPND)_ or *V. harveyi*) infection was analyzed, a significant negative correlation was observed between these metabolites and the bacteria pathogens (Fig. 3B). For instance, taurine (*p<0.001*, *R*^2^= 0.5937) and proline (*p<0.001*, *R*^2^= 0.5883) had a strong negative correlation with *V. parahaemolyticus*_(AHPND)_. Similarly, glycerol (*p<0.001*, *R*^2^= 0.7325) and taurine (*p<0.001*, *R*^2^= 0.5963) had a strong negative correlation with *V. harveyi*. These results indicate that these metabolites are beneficial to the host and therefore important for survival against infection by these pathogens. Most importantly, taurine had a strong correlation with both *V. parahaemolyticus*_(AHPND)_ (*R*^2^=0.5937) and *V. harveyi* (*R*^2^=0.5963)*.*

The mRNA transcripts of some key enzymes involved in taurine metabolism, including cysteine dioxygenase (CDO) (*p<0.01*) and cysteine sulfinic acid decarboxylase (CSD) (*p<0.01*) were significantly downregulated in moribund compared with healthy shrimp, whereas mRNA transcripts of CDO (*p<0.001*), CSD (*p<0.001*), and aspartate aminotransferase (AST) (*p<0.01*) increased in diseased shrimp compared with moribund shrimp (Fig. 3C–E). Similarly, several metabolites in taurine metabolism, such as l-cysteine (*p<0.001*), hypotaurine (*p<0.001*), and taurine (*p<0.01*) were significantly attenuated in moribund compared with healthy shrimp, while increased levels of l-cysteine (*p<0.001*), hypotaurine (*p<0.01*), and taurine (*p<0.001*) were observed in diseased compared with moribund shrimp (Fig. 3F). When the GC-MS metabolomics data (Fig. 3G–K) was validated using ELISA, similar results (Fig. 3L–P) were observed, except glycerol (Fig. 3N). Nonetheless, given that different sample sizes were used for the GC-MS analysis (i.e., 26 samples per group) and the ELISA validation (6 samples per group), there was a slight variation in the two datasets. For instance, while levels of hypoxanthine (Fig. 3J, O) and uracil (Fig. 3K, P) were decreased in diseased shrimp, their levels increased in moribund shrimp (Fig. 2F and Fig. S3A), suggesting that these two metabolites are pro-bacterial because they could be used by the infecting bacteria to proliferate and cause harm to the host, as observed in diseased and moribund shrimp.

### Taurine metabolism is essential for shrimp survival during *Vibrio* infection

**Fig. 4:**
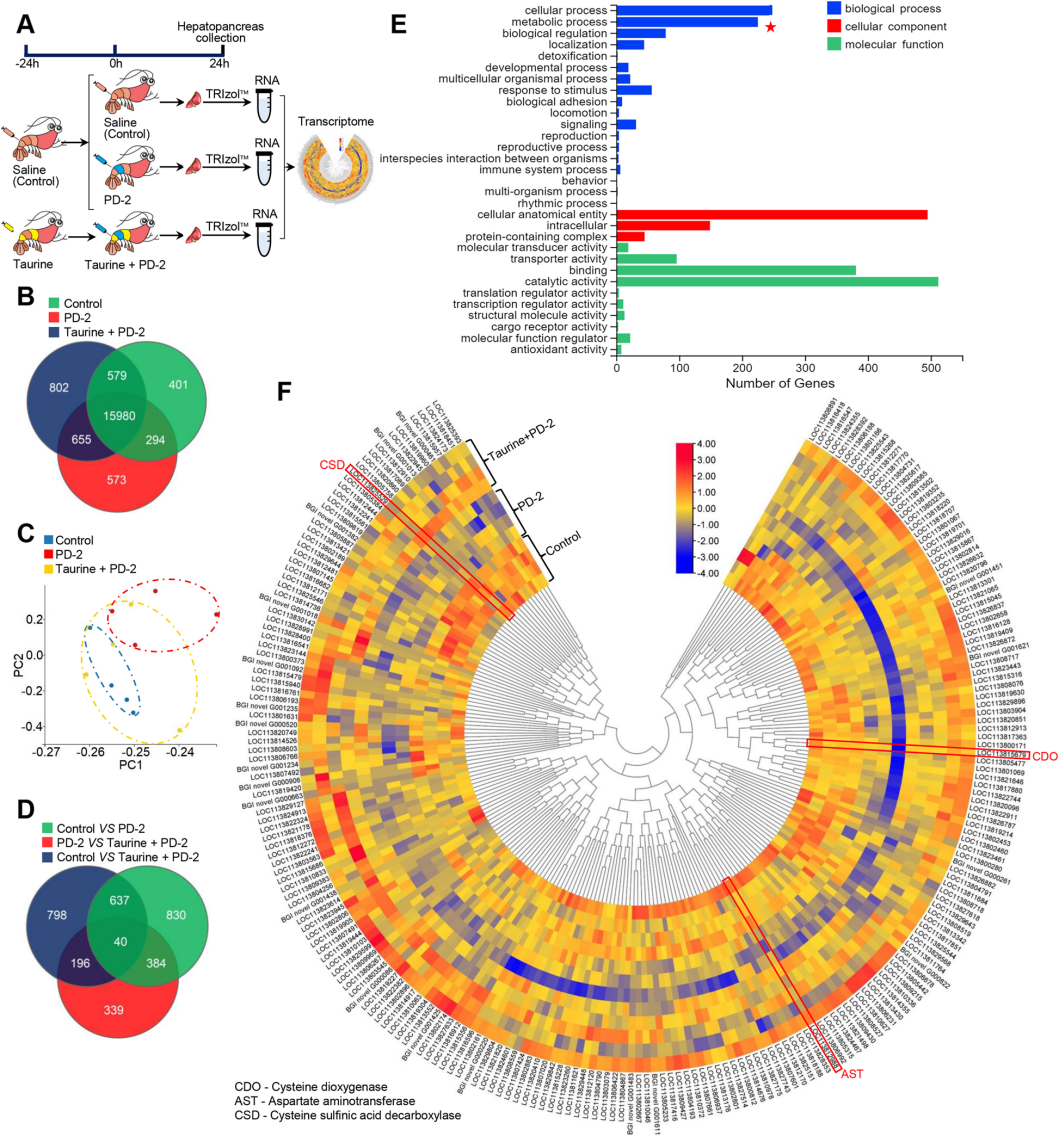
Analysis of genes modulated by taurine during vibrio infection. **A** Exogenous treatment of *P. vannamei* with taurine followed by *V. parahaemolyticus* (PD-2) infection. **B** Distribution of expressed genes. **C** Principal component analysis (PCA) based on princomp function. Dots represent independent biological samples. **D** Distribution of significant (*p*<0.05) differentially expressed genes (DEGs). **E** Gene ontology (GO) functional enrichment analysis of significantly downregulated DEGs (*V. parahaemolyticus* (PD-2) vs control or taurine + *V. parahaemolyticus* (PD-2)). **F** Metabolism-related genes identified by GO analysis and shown by heatmap (*n*=4)

To explore the role of taurine in shrimp immune response to *V. parahaemolyticus* (isolate PD-2) infection, hepatopancreas transcriptome analysis was performed using three cDNA libraries, i.e., control, PD-2, and taurine+PD-2 (Fig. 4A). A total of 19,906 genes were identified (Fig. 4B) that clustered into three groups, as shown by PCA (Fig. 4C), with the control (saline) and taurine+PD-2 groups being closer. These results suggest that the hepatopancreas transcriptome could be modulated by *V. parahaemolyticus* infection, while exogenous taurine attenuates the effect of the bacteria-induced metabolic changes.

Based on *p*<0.05 as the cutoff, 1891 differentially expressed genes (DEGs) were found when control and PD-2 groups are compared and 959 DEGs when PD-2 and taurine+PD-2 are compared, while 1671 DEGs were found by comparing control and taurine+PD-2 groups (Fig. 4D). Among these DEGs, 1169 were significantly downregulated in the PD-2 vs control or taurine+PD-2 groups (Table S3). Thus, to explore how dietary taurine supplementation could modulate the expression of these downregulated genes to boost shrimp survival against pathogens, we went on to further analyze these 1169 significantly downregulated DEGs. Gene ontology (GO) analysis of the 1169 significantly downregulated DEGs revealed that these genes were mainly enriched in cellular process and metabolic process (biological process category), cellular anatomical entity and intracellular (cellular component category), and catalytic activity and binding (molecular function category) (Fig. 4E). To identify metabolites that modulate shrimp response to infection, genes involved in metabolic processes were selected (Fig. 4F).

**Fig. 5:**
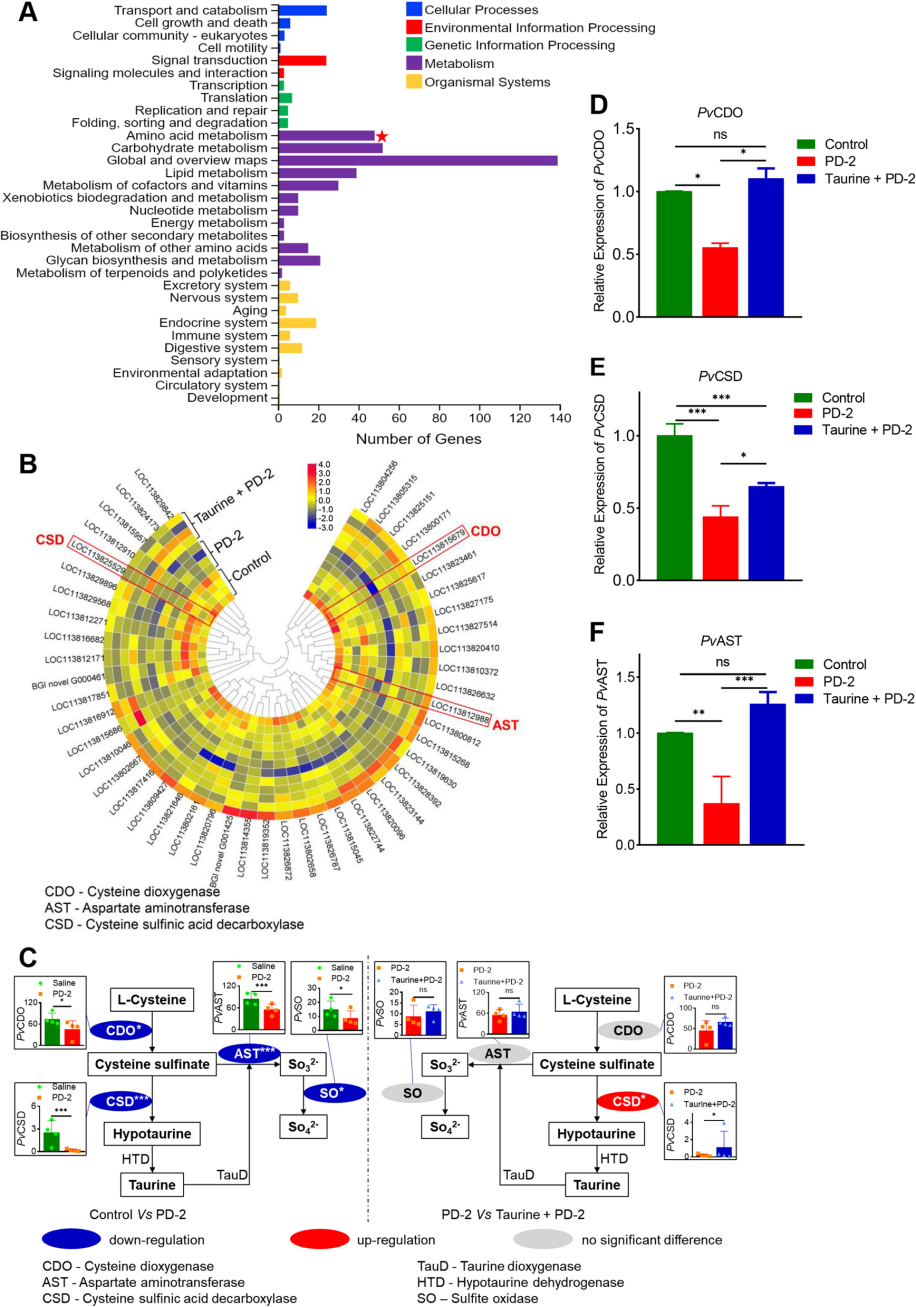
Annotation of genes significantly downregulated by *V. parahaemolyticus* (PD-2) with or without taurine treatment. **A** KEGG pathway enrichment analysis and annotation of significantly downregulated DEGs (*V. parahaemolyticus* (PD-2) vs control or taurine + *V. parahaemolyticus* (PD-2)). **B** Amino acid metabolism-related genes identified from KEGG pathway enrichment analysis and shown by heatmap (*n*=4). **C** Schematic diagram showing the expression of key taurine metabolism-related enzymes with and without *V. parahaemolyticus* (PD-2) challenge. Relative expression of **D** CDO gene, **E** CSD gene, and **F** AST gene. Results were reported as mean ± S.E.M (*n* = 3). *p < 0.05; **p < 0.01; ***p < 0.001

When the 1169 DEGs were analyzed using the KEGG pathway analysis, they were enriched in five major pathways, i.e., cellular processes, environmental information processing, genetic information processing, metabolism, and organismal systems (Fig. 5A). Moreover, most of these genes were enriched in metabolism pathways, especially, global and overview maps, carbohydrate, amino acid, and lipid metabolism. Given the importance of amino acids and their metabolites in the immune response and immune-related signaling pathways in crustaceans [33, 73], we decided to focus on amino acid metabolism in the rest of the study. Thus, further analysis of the 48 genes enriched in amino acid metabolism (Fig. 5B) revealed that these genes were mainly enriched in the taurine metabolism pathway (Fig. 5C). The mRNA transcript levels of cysteine dioxygenase (CDO), cysteine sulfinic acid decarboxylase (CSD), and aspartate aminotransferase (AST), the rate-limiting enzymes in taurine synthesis, decreased significantly after shrimp were challenged with *V. parahaemolyticus* (PD-2) (Fig. 5D–F). However, when shrimp were treated with exogenous taurine followed by *V. parahaemolyticus* (PD-2) challenge, mRNA transcript levels of CSD and CDO, but not AST, increased significantly compared with taurine untreated shrimp challenged with PD-2 (Fig. 5D–F). These results indicate that taurine and its metabolic products play essential roles in shrimp’s antibacterial immune response.

**Fig. 6:**
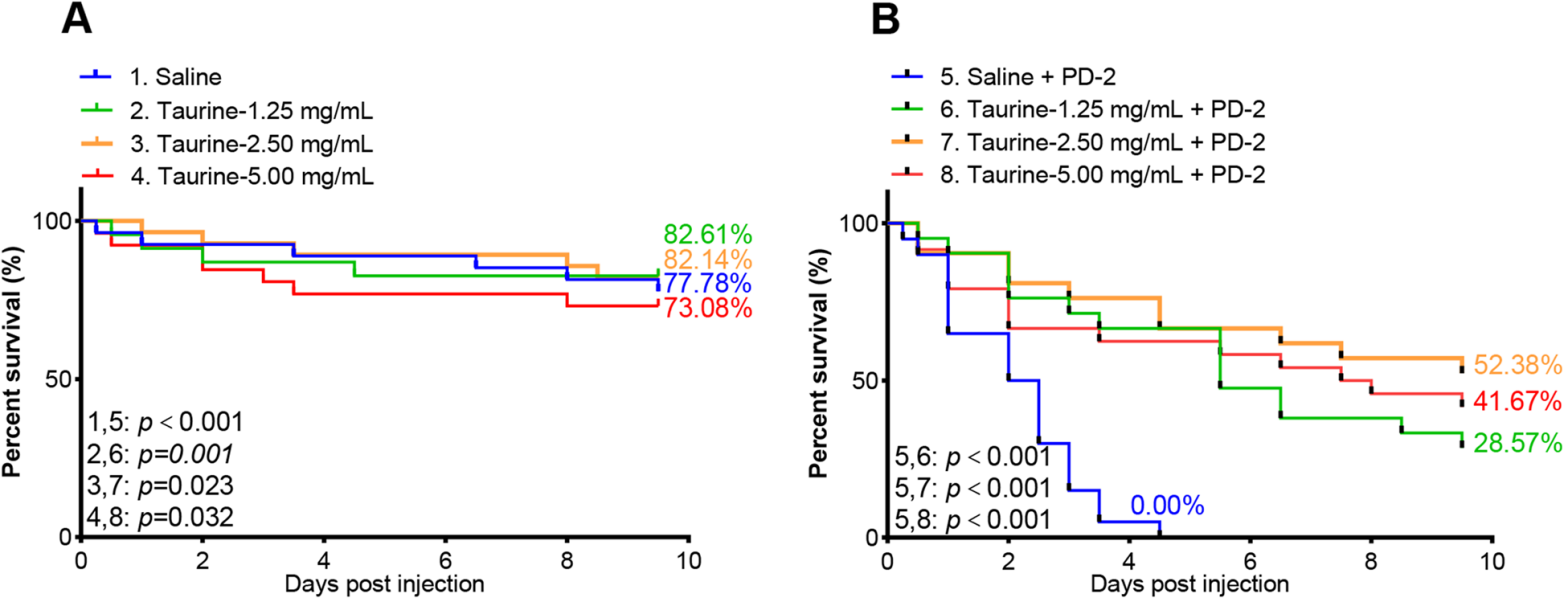
Effect of exogenous taurine on shrimp survival. Shrimp survival rate after **A** exogenous taurine treatment and **B** exogenous taurine treatment plus *V. parahaemolyticus*_(AHPND)_ challenge and recorded. The product-limit method of Kaplan–Meier was used to calculate shrimp survival rate and the significance compared using the log-rank test

To further ascertain the role of taurine in shrimp survival upon vibrio infection, shrimp were injected with taurine (1.25, 2.50, and 5.00 mg/mL) before being challenged with *V. parahaemolyticus* (PD-2). Shrimp survival rates were 28.57, 52.38, and 41.67%, respectively, compared with untreated shrimp challenged with PD-2 (Fig. 6A, B). The highest survival rate was observed when shrimp were injected with 2.50 mg/mL of taurine (*p<0.001*). These results indicate that appropriate amounts of taurine supplementation could enhance shrimp survival against *V. parahaemolyticus* (PD-2) infection.

## Discussion

The World Organization for Animal Health (OIE) has listed acute hepatopancreas necrosis disease (AHPND) as one of the seven most infectious bacterial diseases that impact shrimp aquaculture. Since its discovery, AHPND has become the most pathogenic and destructive disease that affects shrimp aquaculture due to its acute onset, high fatality rate, and widespread infection [4]. In the current study, diseased and moribund penaeid shrimp (*P. vannamei*) displayed massive gross and histological damage to their GITs (Fig. 1) and were found to be mainly infected with two strains of *Vibrio*, i.e., *V. harveyi* and *V. parahaemolyticus* (the strain that causes AHPND), which altered the levels of key metabolites essential for shrimp survival. Atrophied hepatopancreas, shedding of hepatic tubules, and empty GIT are the typical pathological characteristics of AHPND in penaeid shrimp [59, 74]. Although these two *Vibrio* species (*V. harveyi* and *V. parahaemolyticus*_(AHPND)_) seem to be responsible for the pathological features in the diseased and moribund shrimp, none of these bacteria was found in the gut and hemolymph of diseased shrimp, which could be due to the gradual clearance of the bacteria by the host immune system, but their released toxins could damage shrimp tissues [75]. The hepatopancreas was found to contain the highest relative abundance of *Vibrio* in both diseased and moribund shrimp, and since it is also the main metabolic organ in crustaceans [76, 77], it was used for the rest of the studies.

Some metabolites have direct immune functions [78], while others modulate immune response indirectly via other factors or pathways [79–81]. After metabolomic profiling of hepatopancreas samples from healthy, diseased, and moribund shrimp, the metabolites that enhance shrimp survival against the bacterial pathogens (*Vibrio*) were identified as those that were significantly dysregulated in diseased (resistant) and moribund (susceptible) shrimp. This criterion is based on the assumption that metabolites upregulated in diseased (resistant) shrimp but decreased in moribund (susceptible) shrimp should be crucial metabolites that enhance shrimp immune resistance against the infection. Indeed, strong negative correlations were observed between relative bacteria abundance (*V. parahaemolyticus* (PD-2) and *V. harveyi*) and several key metabolites, including taurine, proline, aspartic acid, glycerol, pinitol, malic acid, uridine, and inosine (Fig. 3B). These metabolites play important direct or indirect roles in shrimp immune response, given that dietary proline supplementation (2.29–2.34%) in low fishmeal diets could improve antioxidant capacity, immune response, and ammonia stress tolerance in *P. vannamei* [82]. Similarly, bioflocs grown on glycerol as the carbon source could protect brine shrimp (*Artemia franciscana*) larvae against *V. harveyi* infection [83]. Although malic acid and aspartic acid have not been implicated in shrimp immune response, they can increase the immune response of aquatic animals, such as common carp (*Cyprinus carpio*) [84] and zebrafish (*D. rerio*) [85]. The role of inosine and uridine in immune response has only been reported in mammals, including an anti-inflammatory effect of inosine in mice [86] and improved intestinal development and growth performance by uridine in piglets [87]. Thus, these metabolites (taurine, proline, aspartic acid, glycerol, pinitol, malic acid, uridine, and inosine) could enhance the immune response of shrimp against pathogens, given that their levels increased in diseased shrimp but decreased in moribund shrimp (Fig. 3A).

In host–pathogen interaction, metabolome modulation could be a strategy by the host to clear the invading pathogen [88] or that adopted by the pathogen to evade the host immune surveillance [89, 90]. Indeed, levels of some metabolites, including lactic acid, putrescine, cadaverine, hypoxanthine, xanthine, etc. that were downregulated in diseased (resistant) shrimp but increased in moribund (susceptible) shrimp, had a strong positive correlation with bacteria abundance (*V. parahaemolyticus* (PD-2) and *V. harveyi*), suggesting that these metabolites are beneficial to the pathogens. This observation is consistent with previous studies, where *P. vannamei* challenged with *Vibrio campbellii* had decreased oxygen uptake and increased lactic acid levels [91]. Given that most *Vibrio* species are facultative anaerobic bacteria, anaerobic conditions and lactic acid are more conducive to their growth and replication. Increased levels of lactic acid and putrescine have also been observed in the hepatopancreas of *P. vannamei* upon white spot syndrome virus (WSSV) infection [17]. Although no studies have thus far reported the role of hypoxanthine in shrimp, many *Neisseria gonorrhoeae* isolates from patients with disseminated gonococcal infection require arginine, hypoxanthine, and uracil for their growth [92]. Thus, levels of metabolites that increased in moribund shrimp are beneficial to the pathogens because they could be essential for their proliferation and or enable them to escape the host immune response.

In most animal tissues, taurine exists as an abundant free amino acid, involved in many crucial biological processes [36, 93–97], including innate immune response [41–43]. In mammals, taurine mediates the AMPK-mTOR [44] and TLRs/NF-κB [45] pathways to inhibit excessive activation of inflammatory responses to reduce cell damage. Similarly, the antioxidant properties of taurine [98, 99] are important for cells’ protection under acute inflammatory conditions [100] and enhancement of immune and antioxidant responses [47, 49]. Most importantly, taurine improves mammalian host defense against pathogens [37, 101–103] by potentiating the immune defense abilities of lymphocytes [104], neutrophils [105, 106], and macrophages [107]. Although there is currently limited information on the role of taurine in shrimp immune response, dietary taurine supplementation could enhance the survival and immune response of several marine organisms. For instance, exogenous taurine can reduce ammonia toxicity in juvenile yellow catfish (*Pelteobagrus fulvidraco*) by regulating inflammatory factors [46], improving immune response and antioxidant indices [47], regulating the expression of innate immune genes to enhance antibacterial (*Vibrio alginolyticus*) response in zebrafish [48], and improving cholesterol metabolism to enhance the antimicrobial immune response in yellowtail *Seriola quinqueradiata* [108]. Similarly, exogenous taurine upregulates the expression of immune genes and enhances antioxidant capacity in the Chinese mitten crab *Eriocheir sinensi* [49], improves the antioxidant response of European seabass (*Dicentrarchus labrax*) [50], and enhances the survival rate of Crucian Carps against *Edwardsiella tarda* [51]. In the present study, mRNA transcript levels of rate-limiting enzymes of taurine metabolism, i.e., *CDO*, *CSD*, and *AST*, were attenuation by *V. parahaemolyticus* (PD-2) infection, while taurine supplementation could induce *CDO* and *CSD* expression, except *AST*, to enhance shrimp (*P. vannamei*) survival against *V. parahaemolyticus* (PD-2) infection (Fig. 5).

**Fig. 7:**
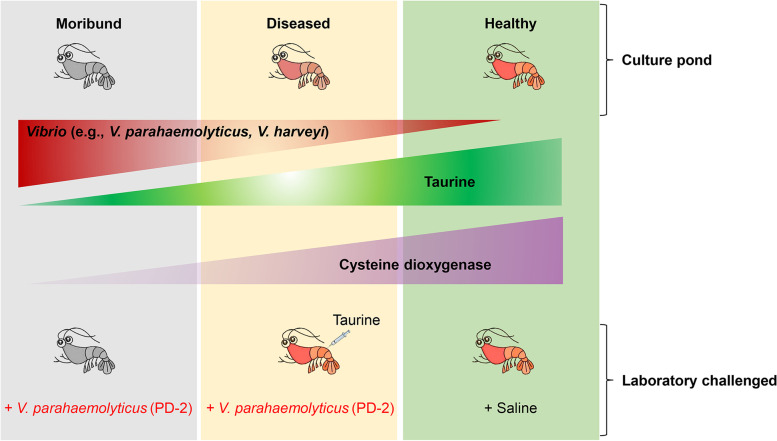
Proposed schematic illustration of the mechanism by which taurine metabolism is modulated during infection of shrimp by *V. parahaemolyticus* (AHPND strain)

Our present data indicate that taurine metabolism is dysregulated by bacterial (*Vibrio*) pathogens, while exogenous taurine restores taurine metabolism under pathogen infection. Nonetheless, our results do not rule out some effect of taurine on the host’s microbiota [38]. For instance, in mice, exogenous taurine enhanced the production of sulfides, cellular respiration inhibitors of most pathogens, to remodel microbiota functionally and enhance resistance to *Klebsiella pneumonia* [37]. Here, transcript levels of the enzymes that catalyze sulfide production, i.e., aspartate aminotransferase (AST) (*p<0.001*) and sulfite oxidase (SO) (*p<0.05*), were significantly decreased after *V. parahaemolyticus* (PD-2) challenge. However, taurine supplementation followed by *V. parahaemolyticus* (PD-2) challenge improved taurine metabolism and increased the mRNA transcript levels of AST and SO, although not statistically significant. It is therefore conceivable that taurine could modulate microbiota and host immune system to enhance shrimp immune response against bacterial pathogens such as *V. parahaemolyticus* (PD-2). We illustrate diagrammatically (Fig. 7), a proposed mechanism of the dysregulation of taurine metabolism in diseased shrimp and how optimal taurine supplementation enhances shrimp survival against *V. parahaemolyticus* (AHPND-causing strain) and by extension other pathogenic bacteria.

## Conclusion

The present study reveals that diseased and moribund shrimp, displaying empty and atrophied GITs, were mainly infected with pathogenic vibrio (mainly AHPND causing *V. parahaemolyticus*). These bacteria induced the dysregulation of many metabolites, including taurine, which was upregulated in diseased shrimp but downregulated in moribund shrimp. Moreover, a certain concentration of dietary supplemented taurine, in this study, 2.5 mg/mL, enhanced shrimp survival by more than 50% when stimulated with *V. parahaemolyticus* (PD-2), indicating that an optimal amount of taurine could significantly enhance penaeid shrimp’s antibacterial response. Therefore, these findings could be leveraged to improve the aquaculture of shrimp and other crustaceans.

## Supplementary Information

**Additional file 1: Figure S1.** PCR screening of 16S rRNA gene for identification of pathogenic bacteria in shrimp. PCR analysis of (A) total bacteria (16S rRNA gene), (B) *Vibrio*-specific (16S rRNA gene, (C) *V. harveyi* (*vhh* gene), (D) *V. parahaemolyticus* (*tlh* gene), (E) *V. parahaemolyticus* (pirB gene), and (F) *V. parahaemolyticus* (AP4 gene) expressed in shrimp hepatopancreas of *P. vannamei*. Numbers 1 – 13: individual shrimp (*P. vannamei*), i: *Streptococcus iniae*, ii: *Vibrio harveyi*, iii: *Vibrio parahaemolyticus* (isolate PD-2). **Figure S2.** Global metabolic profiles of healthy and diseased shrimp. Heat maps showing significantly dysregulated metabolites in the hepatopancreas of (A) Healthy vs diseased shrimp, (B) Healthy vs moribund shrimp, and (C) Diseased vs moribund shrimp. The heat map scale shows green to red, representing low to high abundance. (n=26). (D) Proportion of metabolites categories significantly dysregulated among healthy, diseased, and moribund shrimp. (E) Top 25 KEGG pathway enriched differentially expressed metabolites associated with survival of *P. vanname*i. **Figure S3.** Distribution of metabolites essential for shrimp survival. (A) Immune-related metabolites upregulated in the hepatopancreas of moribund compared with healthy or diseased shrimp. The heat map scale shows green to red, representing low to high abundance. (n=26). (B) Correlation between significantly dysregulated metabolites and the expression of pathogen-specific genes (pirB of *V. parahaemolyticus* and vhh of *V. harveyi*). The heat map scale shows green to red, representing low to high abundance.

**Additional file 2: Supplementary Table 1.** List of primers used for molecular screening of pathogen in the infected shrimp samples along with its product size.

**Additional file 3: Supplementary Table 2.** Normalized area of metabolites in *P. vannamei* hepatopancreas.

**Additional file 4: Supplementary Table 3.** Normalized area of metabolites in *P. vannamei* hepatopancreas.

## Data Availability

The datasets supporting the conclusions of this article are available in the NCBI Sequence Read Archive database with the unique identifier PRJNA813696 (https://www.ncbi.nlm.nih.gov/sra/?term=PRJNA813696) for the transcriptome dataset and in MetaboLights with the unique identifier MTBLS4770 (http://www.ebi.ac.uk/metabolights/) for the metabolomics dataset.
