# Supplementary material for: Taurine metabolism is modulated in Vibrio-infected Penaeus vannamei to shape shrimp antibacterial response and survival
Source: Microbiome. 2022 Dec 5;10:213. doi: 10.1186/s40168-022-01414-9 (PMC9721036; doi:10.1186/s40168-022-01414-9)
Supplement: Supplementary file 2 — Additional file 1: Figure S1. PCR screening of 16S rRNA gene for identification of pathogenic bacteria in shrimp. PCR analysis of (A) total bacteria (16S rRNA gene), (B) Vibrio-specific (16S rRNA gene, (C) V. harveyi (vhh gene), (D) V. parahaemolyticus (tlh gene), (E) V. parahaemolyticus (pirB gene), and (F) V. parahaemolyticus (AP4 gene) expressed in shrimp hepatopancreas of P. vannamei. Numbers 1 – 13: individual shrimp (P. vannamei), i: Streptococcus iniae, ii: Vibrio harveyi, iii: Vibrio parahaemolyticus (isolate PD-2). Figure S2. Global metabolic profiles of healthy and diseased shrimp. Heat maps showing significantly dysregulated metabolites in the hepatopancreas of (A) Healthy vs diseased shrimp, (B) Healthy vs moribund shrimp, and (C) Diseased vs moribund shrimp. The heat map scale shows green to red, representing low to high abundance. (n=26). (D) Proportion of metabolites categories significantly dysregulated among healthy, diseased, and moribund shrimp. (E) Top 25 KEGG pathway enriched differentially expressed metabolites associated with survival of P. vannamei. Figure S3. Distribution of metabolites essential for shrimp survival. (A) Immune-related metabolites upregulated in the hepatopancreas of moribund compared with healthy or diseased shrimp. The heat map scale shows green to red, representing low to high abundance. (n=26). (B) Correlation between significantly dysregulated metabolites and the expression of pathogen-specific genes (pirB of V. parahaemolyticus and vhh of V. harveyi). The heat map scale shows green to red, representing low to high abundance. [file 40168_2022_1414_MOESM1_ESM.docx]

**Additional file 1.**

**A.**


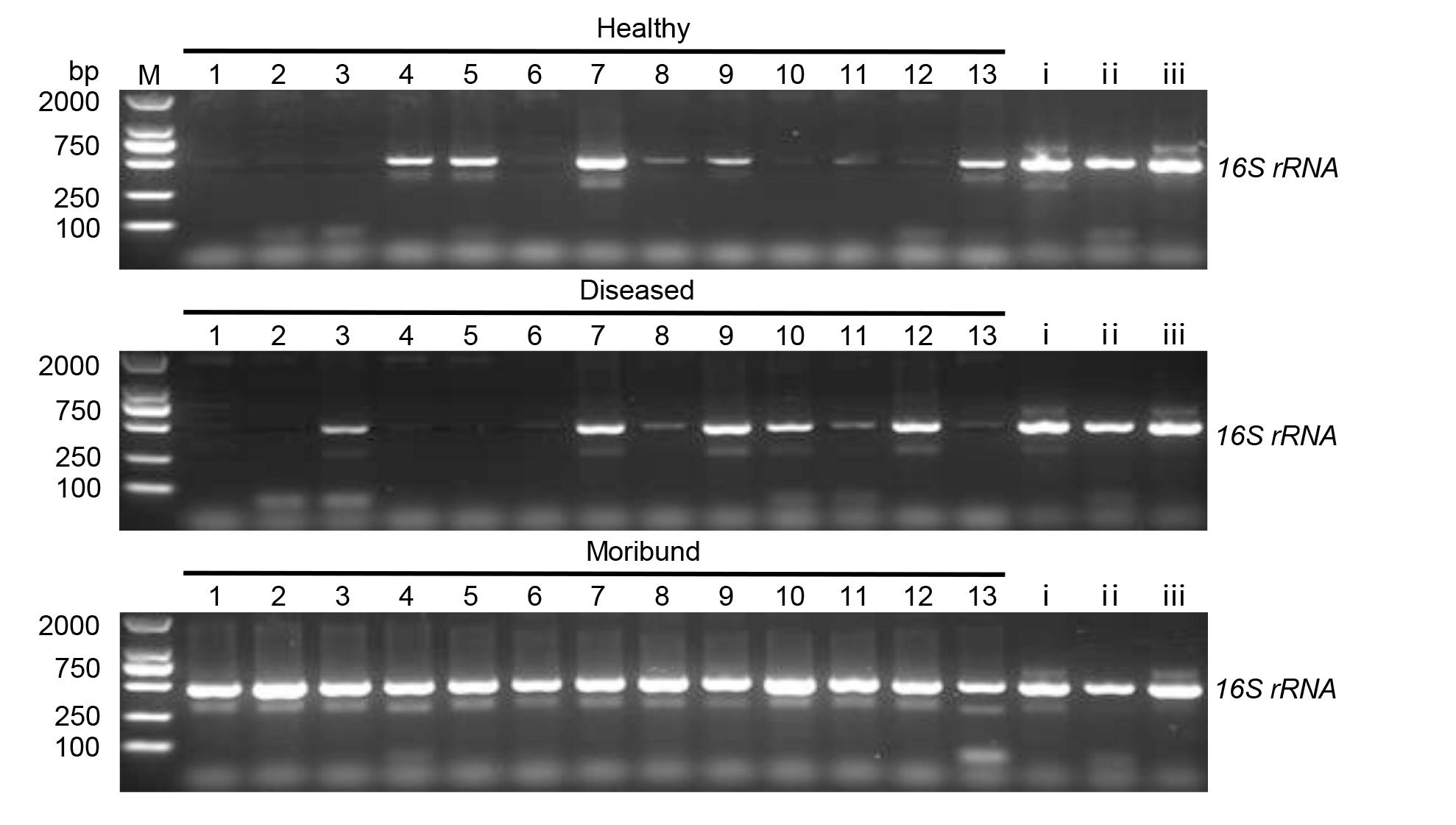


**B.**


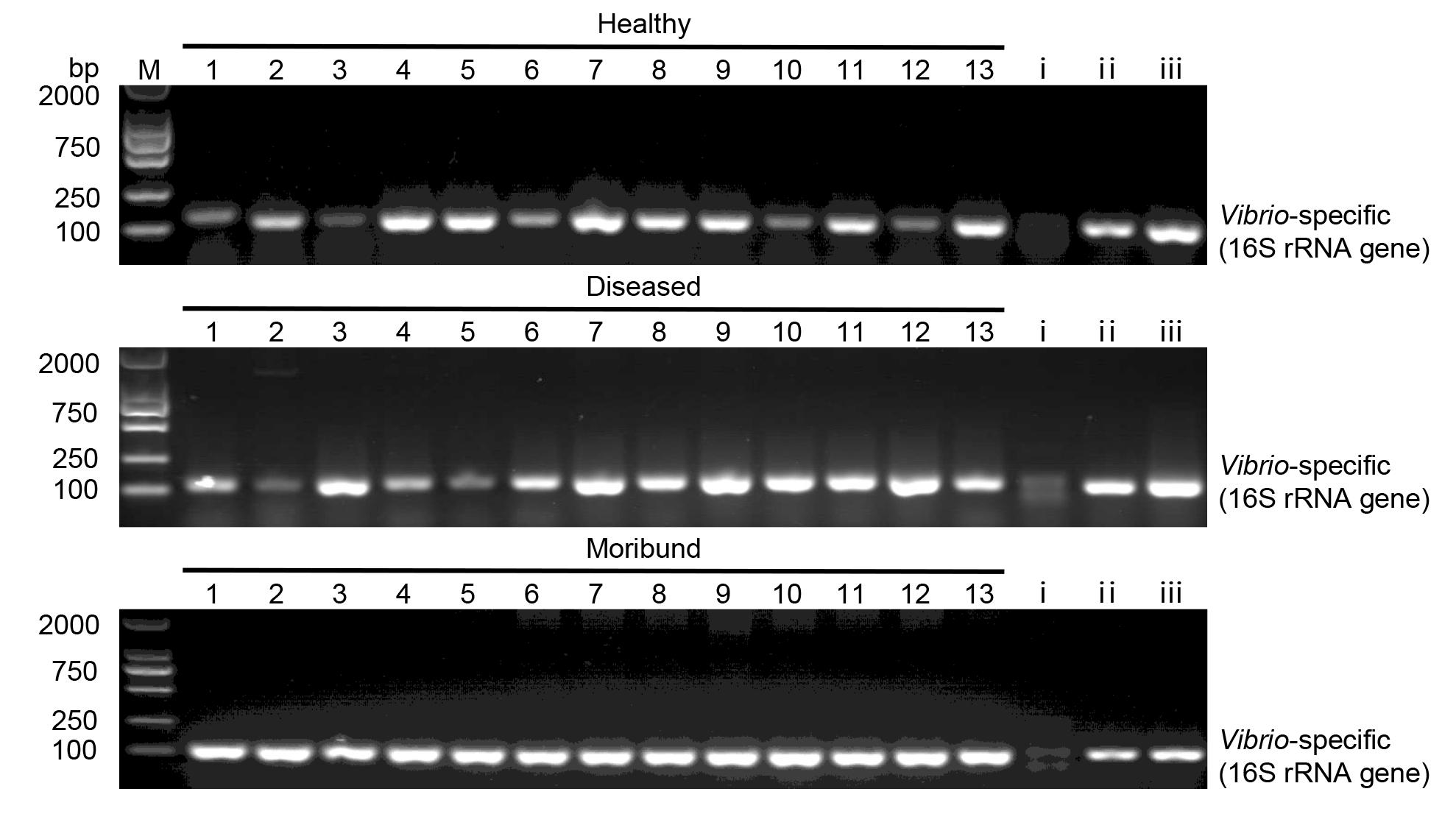


**C.**


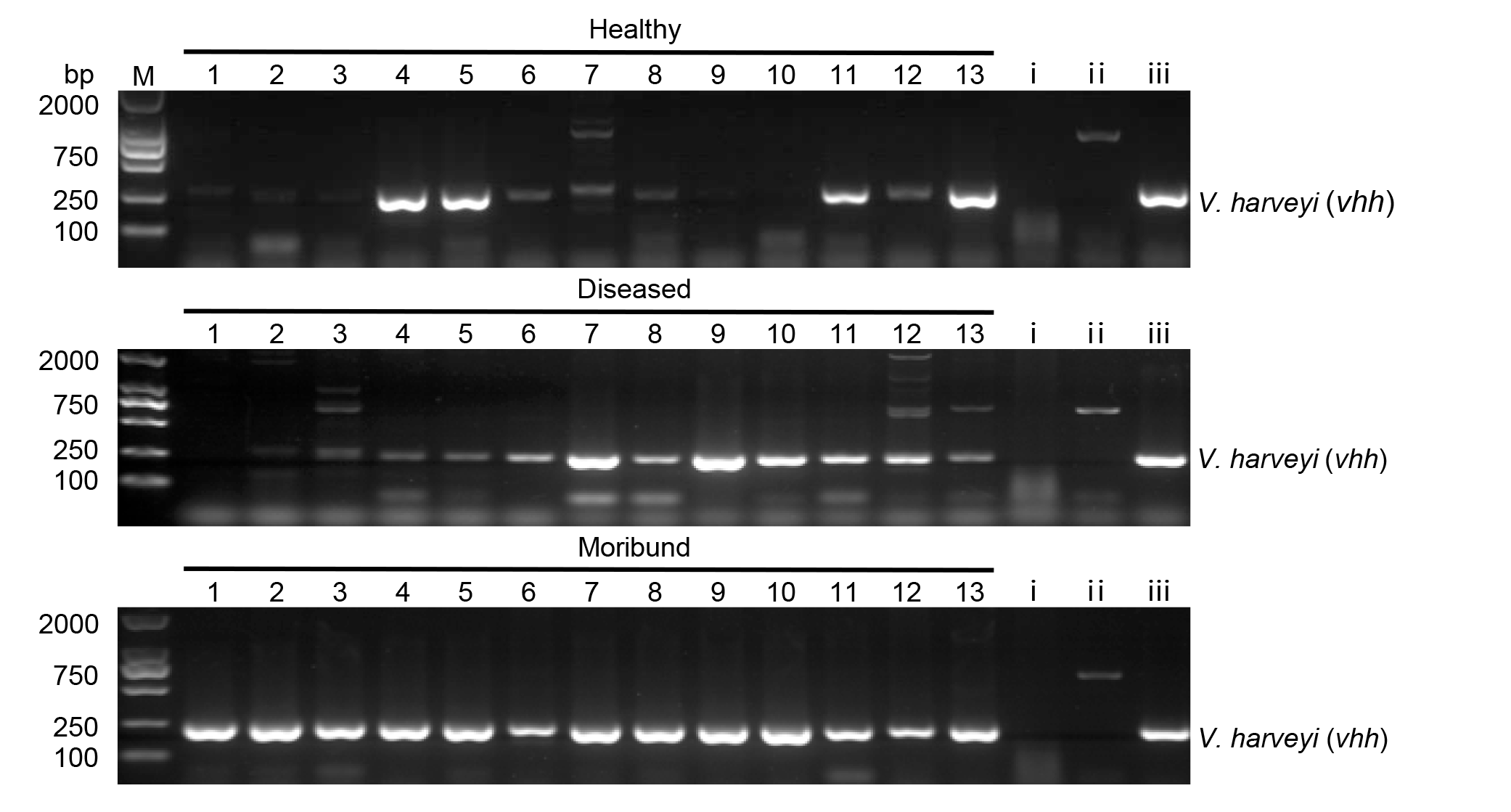


**D.**


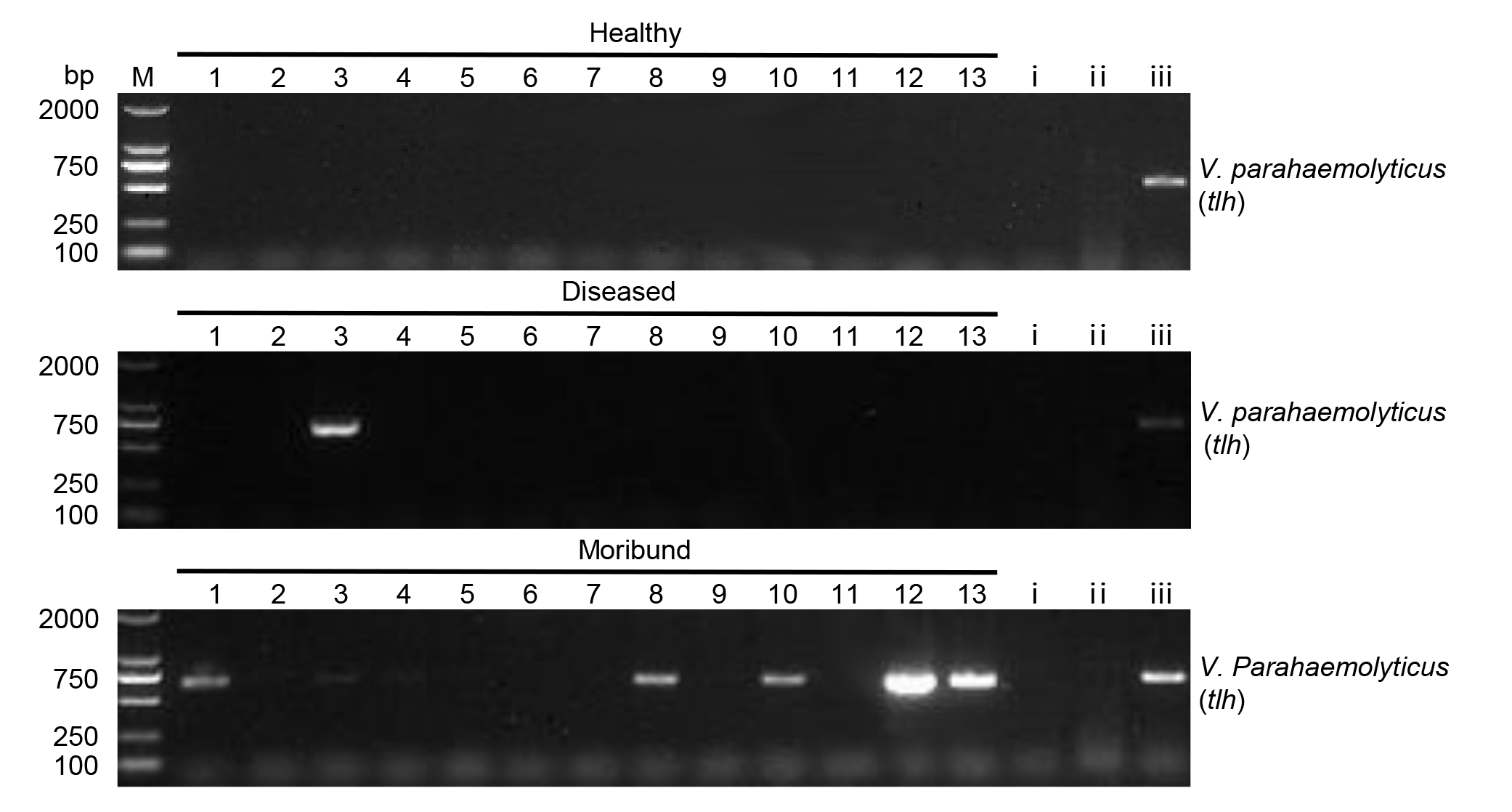


**E.**


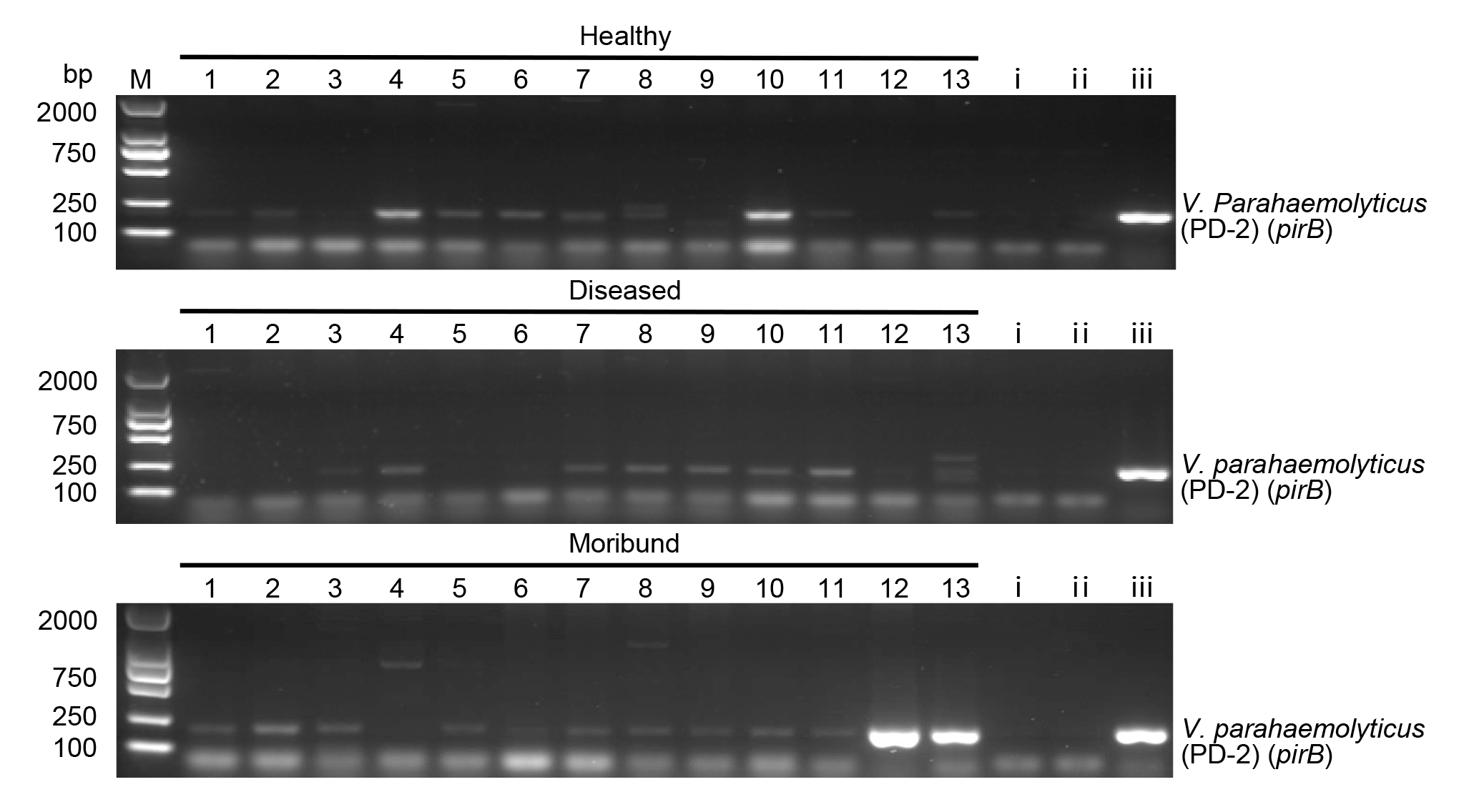


**F.**


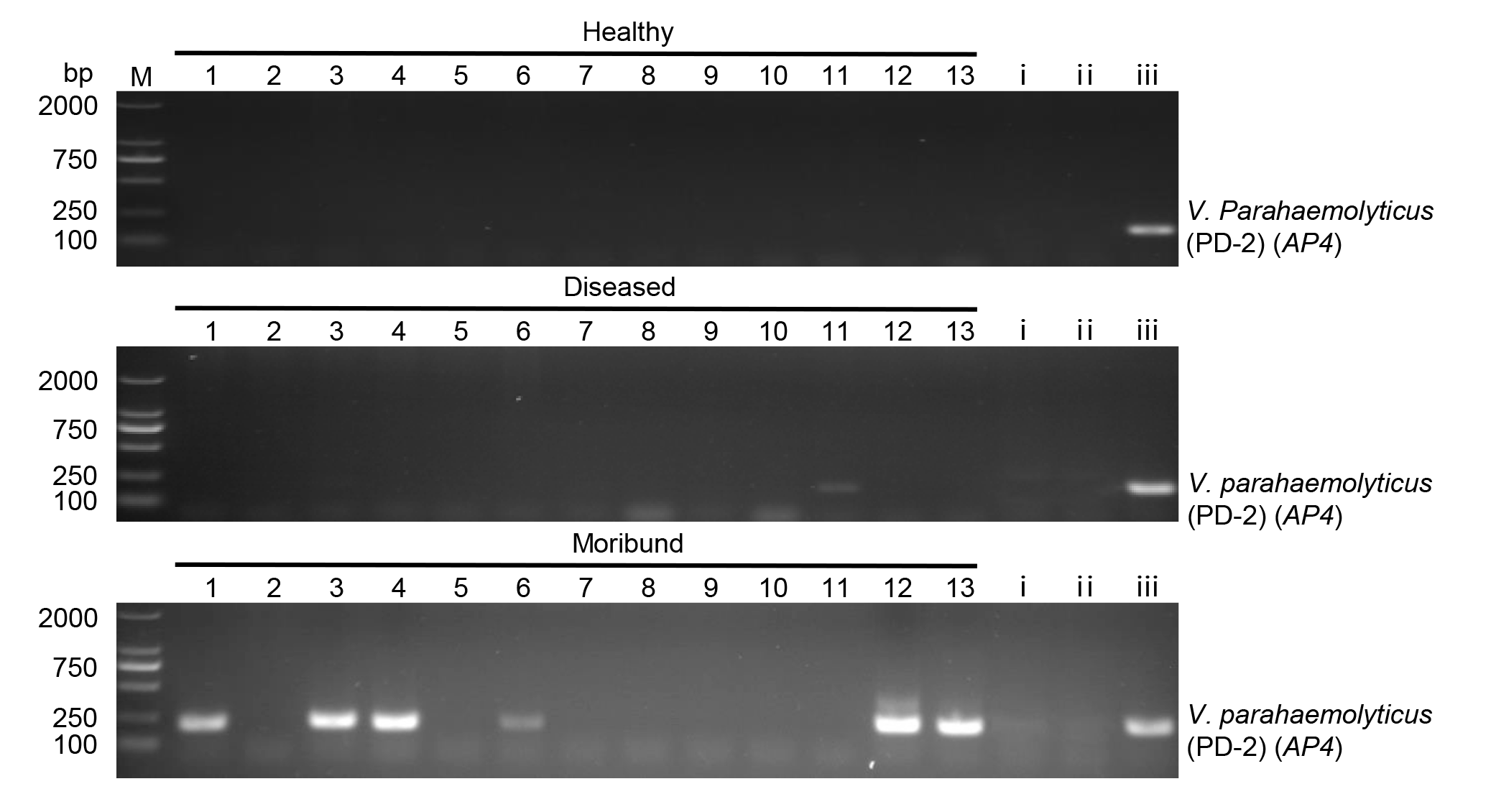


Fi**gure S1.** PCR screening of 16S rRNA gene for identification of pathogenic bacteria in shrimp. PCR analysis of (A) total bacteria (16S rRNA gene), (B) *Vibrio*-specific (16S rRNA gene, (C) *V. harveyi* (*vhh* gene), (D) *V. parahaemolyticus* (*tlh* gene), (E) *V. parahaemolyticus* (pirB gene), and (F) *V. parahaemolyticus* (AP4 gene) expressed in shrimp hepatopancreas of *P. vannamei*. Numbers 1 – 13: individual shrimp (*P. vannamei*), i: *Streptococcus iniae*, ii: *Vibrio harveyi*, iii: *Vibrio parahaemolyticus* (isolate PD-2).

**A.** **B.**


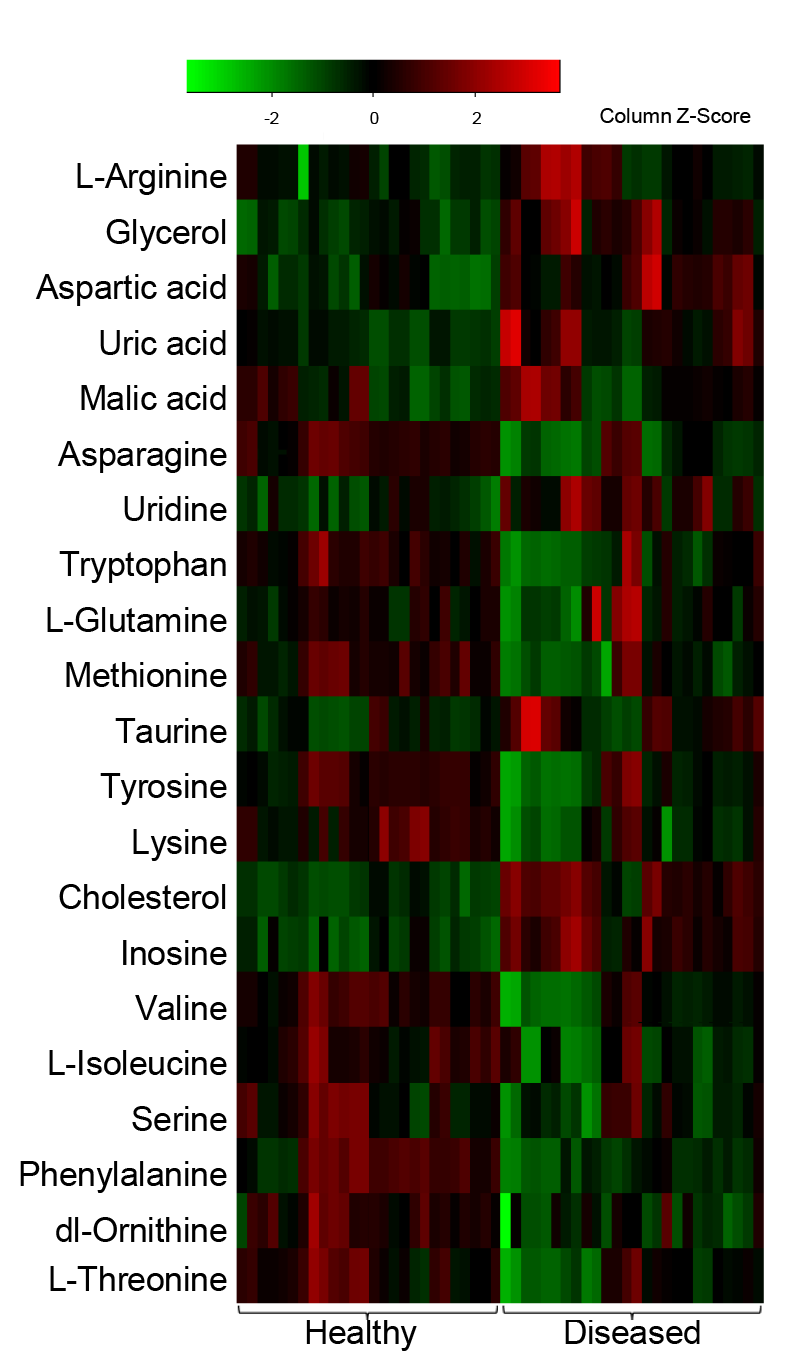

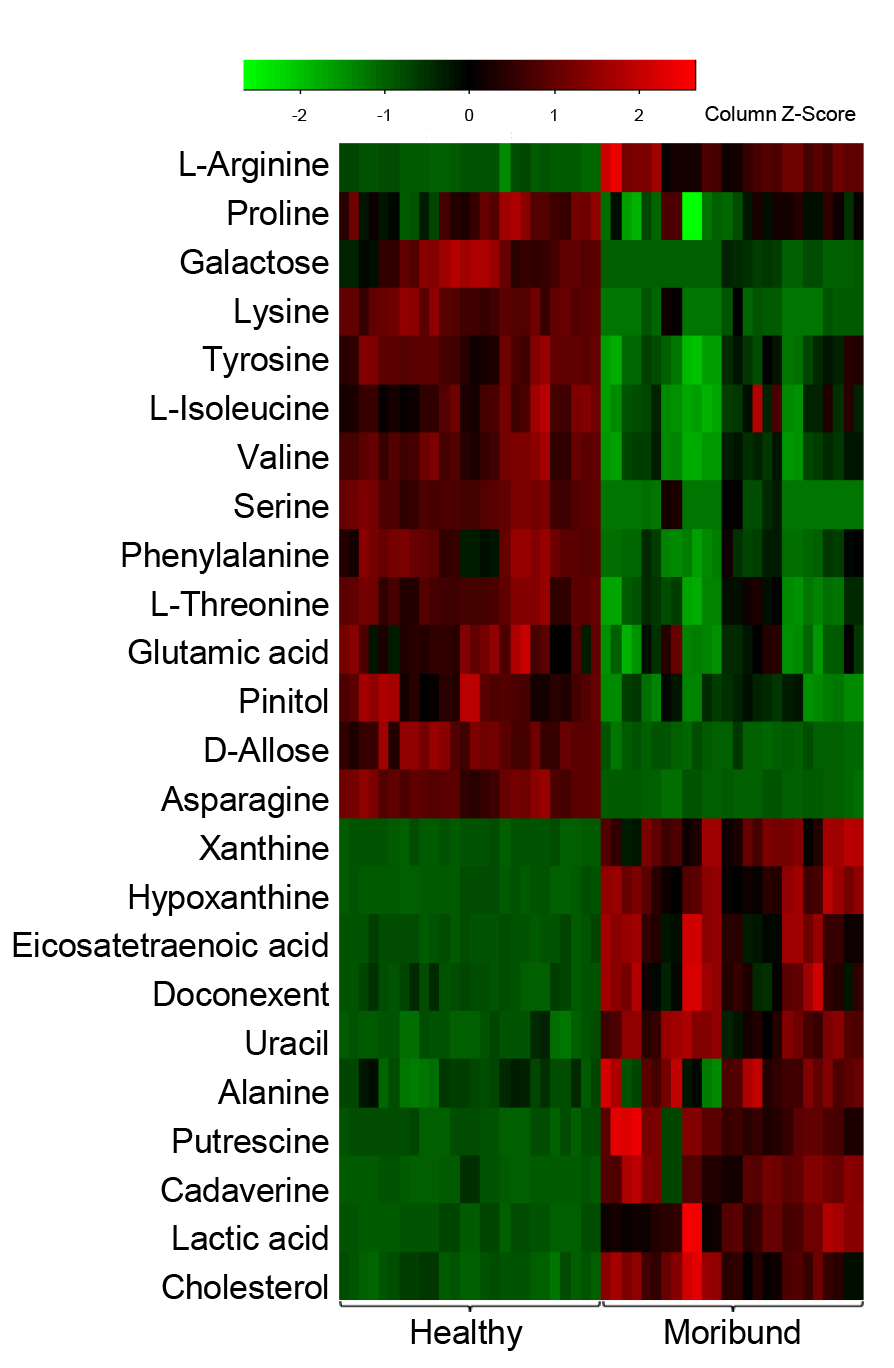


**C.**


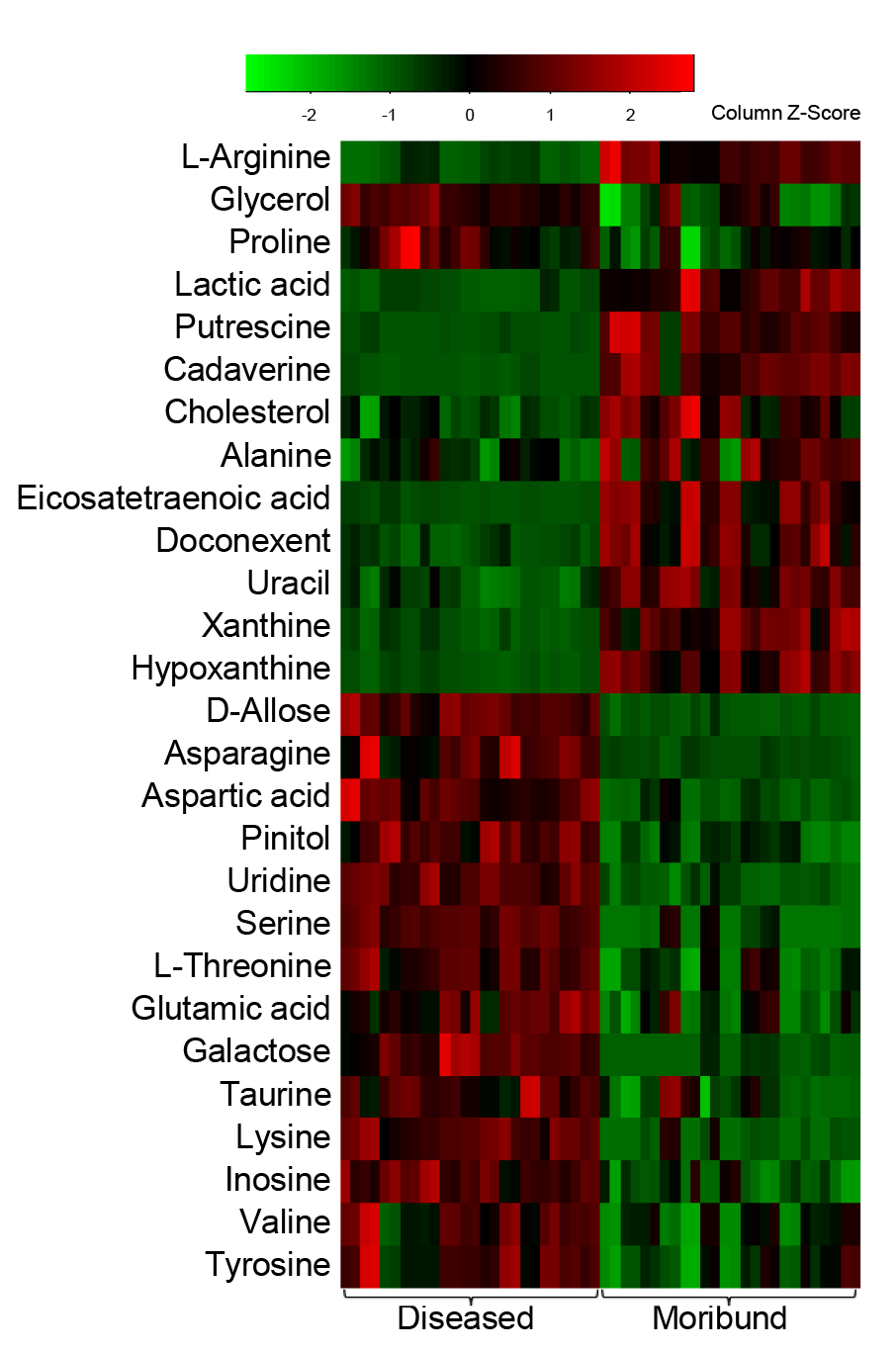


**D.**


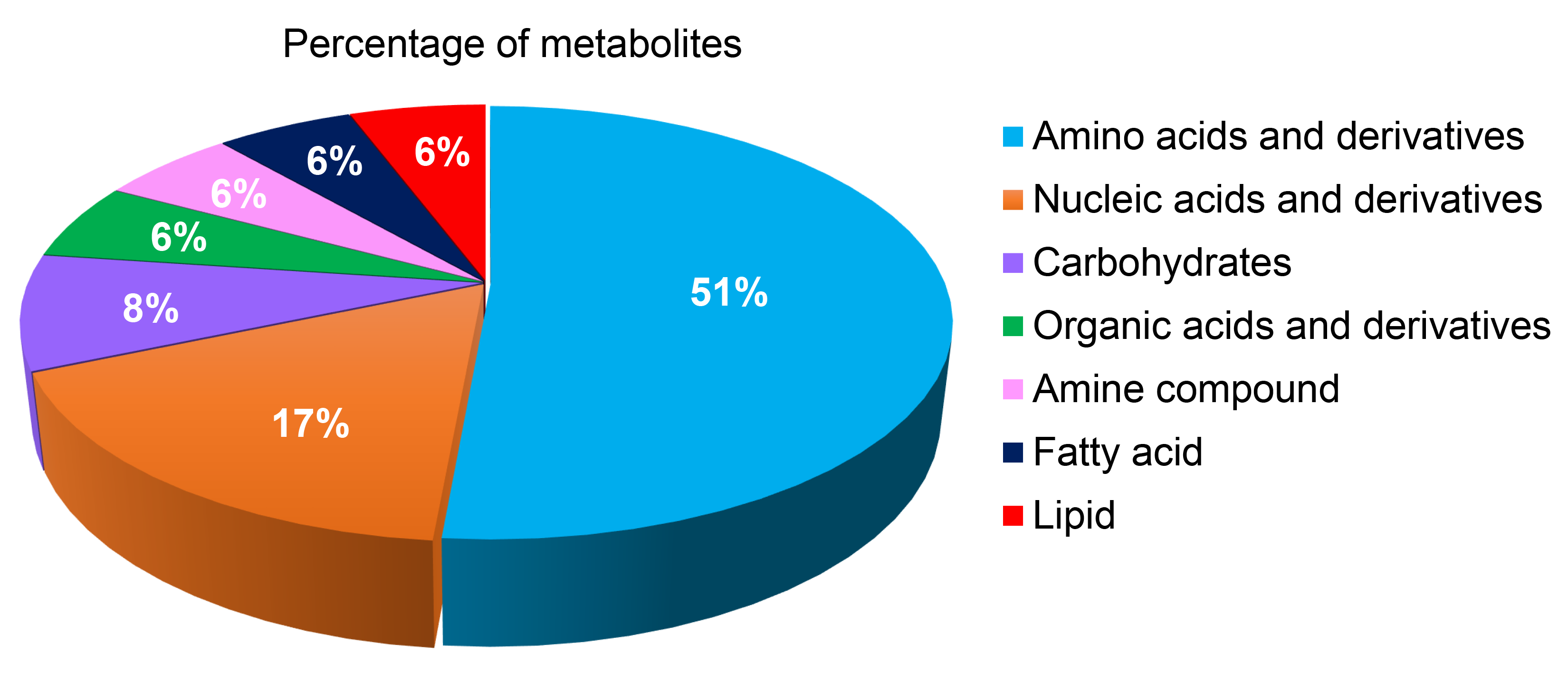


**E.**


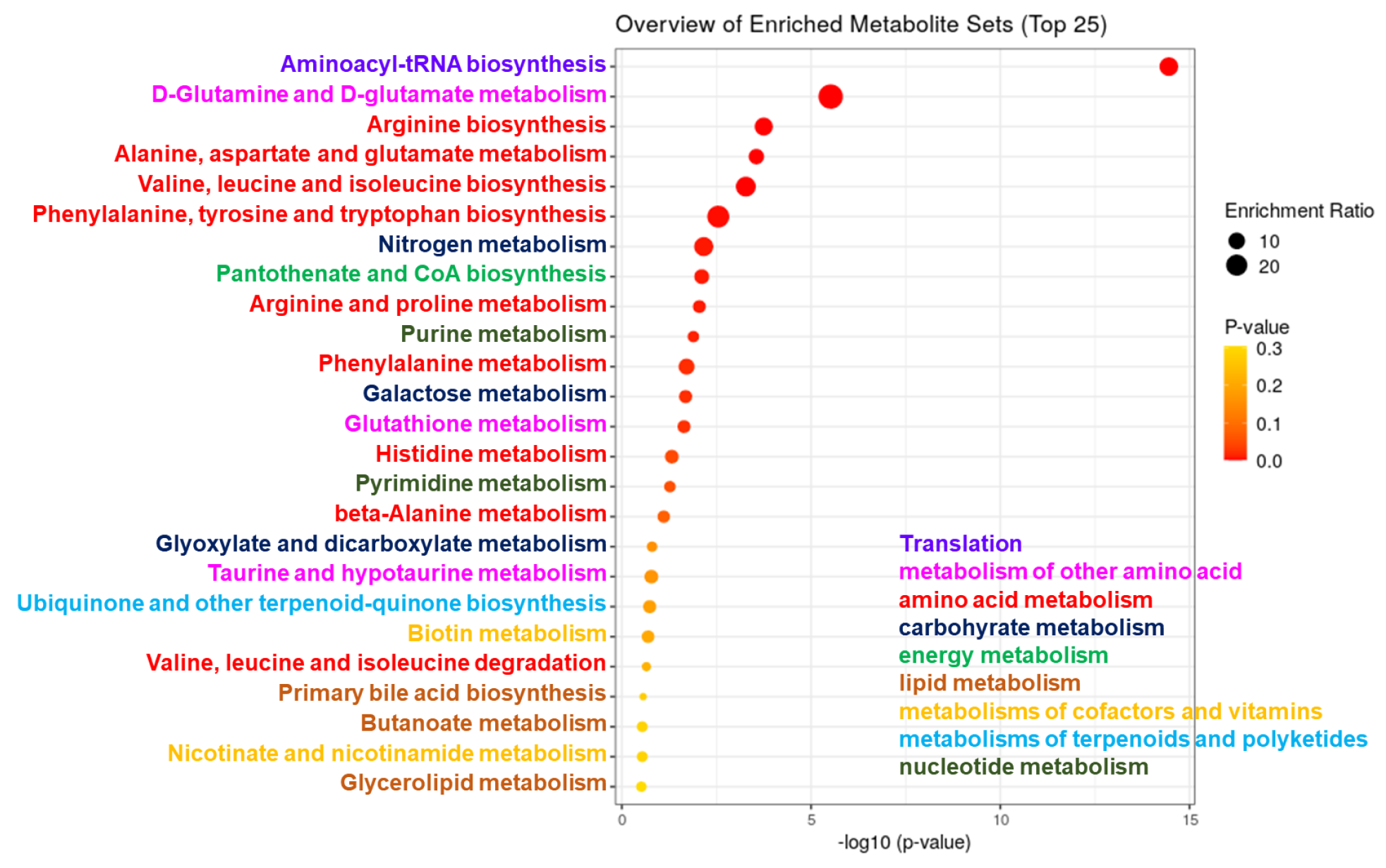


**Figure S2. Global metabolic profiles of healthy and diseased shrimp.** Heat maps showing significantly dysregulated metabolites in the hepatopancreas of (A) Healthy vs diseased shrimp, (B) Healthy vs moribund shrimp, and (C) Diseased vs moribund shrimp. The heat map scale shows green to red, representing low to high abundance. (n=26). (D) Proportion of metabolites categories significantly dysregulated among healthy, diseased, and moribund shrimp. (E) Top 25 KEGG pathway enriched differentially expressed metabolites associated with survival of *P. vanname*i.

**A.**


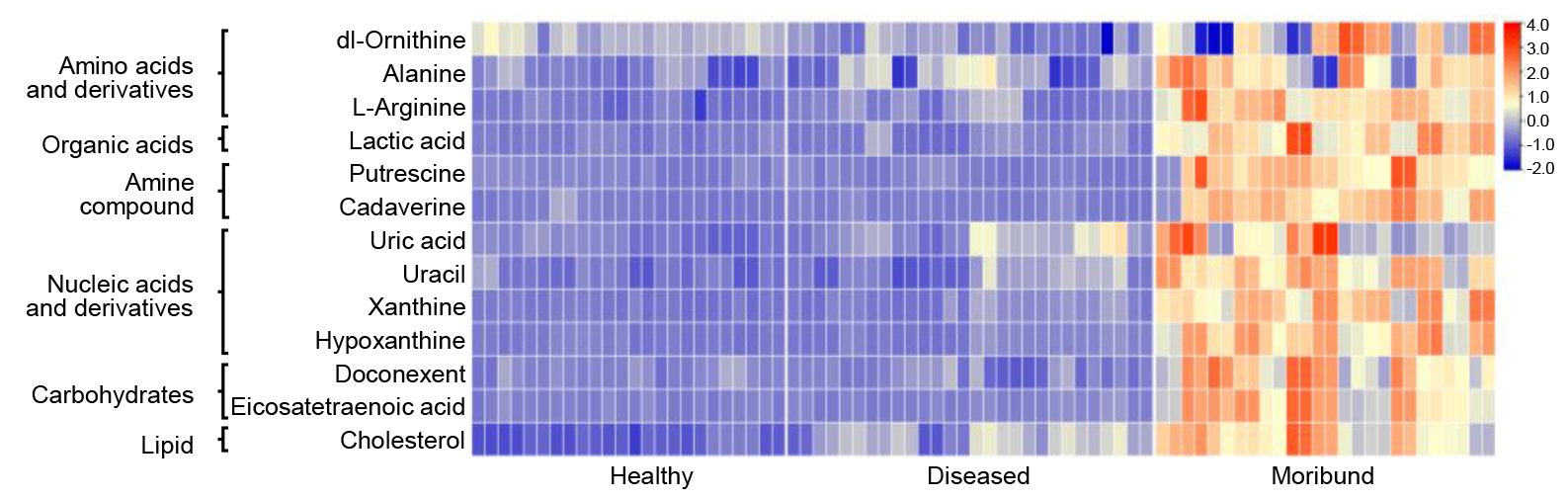


**B.**


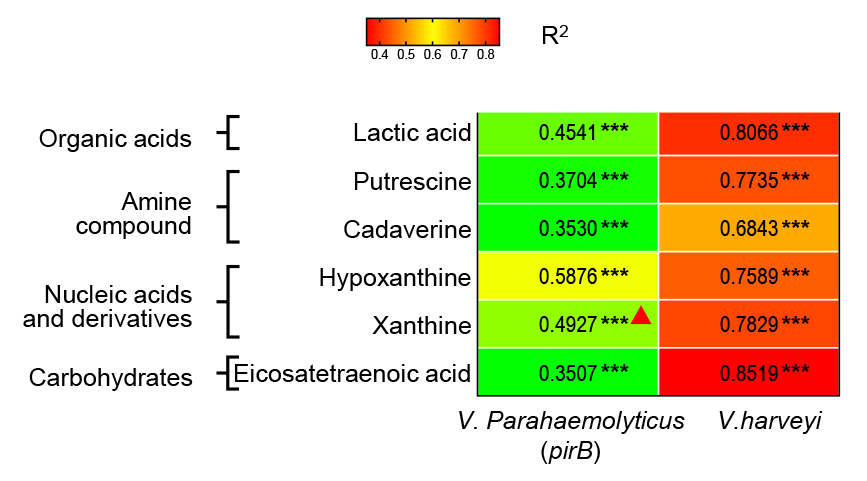


**Figure S3. Distribution of metabolites essential for shrimp survival.** (A) Immune-related metabolites upregulated in the hepatopancreas of moribund compared with healthy or diseased shrimp. The heat map scale shows green to red, representing low to high abundance. (n=26). (B) Correlation between significantly dysregulated metabolites and the expression of pathogen-specific genes (pirB of *V. parahaemolyticus* and vhh of *V. harveyi*). The heat map scale shows green to red, representing low to high abundance.
