# Supplementary material for: Taurine metabolism is modulated in Vibrio-infected Penaeus vannamei to shape shrimp antibacterial response and survival
Source: Microbiome. 2022 Dec 5;10:213. doi: 10.1186/s40168-022-01414-9 (PMC9721036; doi:10.1186/s40168-022-01414-9)
Supplement: Supplementary file 3 — Additional file 2: Supplementary Table 1. List of primers used for molecular screening of pathogen in the infected shrimp samples along with its product size. [file 40168_2022_1414_MOESM2_ESM.docx]

**Supplementary Table 1**. List of primers used for molecular screening of pathogen in the infected shrimp samples along with its product size

| **Genes** | **Primers name** | **Sequence (5′-3′)** | **Target species** | **Amplicon**  **Size (bp)** |
| --- | --- | --- | --- | --- |
| For gene expression | | | | |
| Bacterial 16S rRNA | 16s-338 F | ACTCCTACGGGAGGCAGCAG | Total bacteria | 468 |
|  | 16s-806 R | GGACTACHVGGGTWTCTAAT |  |  |
| *Vibrio*-specific 16S rRNA gene fragment | qV-F | GGCGTAAAGCGCATGCAGGT | *Vibrio* | 114 |
|  | qV-R | GAAATTCTACCCCCCTCTACAG |  |  |
| *V. harveyi* (*vhh*) | qVhh-F | CTTCACGCTTGATGGCTACTG | *V. harveyi* | 253 |
|  | qVhh-R | GTCACCCAATGCTACGACCT |  |  |
| *V. parahaemolyticus* (*tlh*) | VP-tlh-F | GATTTGGCGAACGAGAAC | *V. parahaemolyticus* | 695 |
|  | VP-tlh-R | CGTCTCGAACAAGGCG |  |  |
| *V. parahaemolyticus* (p*irB*) | qPirB-F | GTGGGCTGATAACGACTC | *V. parahaemolyticus* (AHPND) | 187 |
|  | qPirB-R | ACCAACAGCAGGTGAATA |  |  |
| *V. parahaemolyticus*  (AP4) | AP4-F1 | ATGAGTAACAATATAAAACATGAAAC | *V. parahaemolyticus* (AHPND) | 1269 |
|  | AP4-R1 | ACGATTTCGACGTTCCCCAA |  |  |
|  | AP4-F2 | TTGAGAATACGGGACGTGGG |  | 230 |
|  | AP4-R2 | GTTAGTCATGTGAGCACCTTC |  |  |
| Real-time RT-PCR |  |  |  |  |
| Bacterial 16S rRNA | q16S-891F | TGGAGCATGTGGTTTAATTCGA | Total bacteria | 113 |
|  | q16S-1003R | TGCGGGACTTAACCCAACA |  |  |
| *Vibrio*-specific 16S rRNA gene fragment | qVibrio-F | GGCGTAAAGCGCATGCAGGT | *Vibrio* | 114 |
|  | qVibrio-R | GAAATTCTACCCCCCTCTACAG |  |  |
| *V. harveyi* (*vhh*) | qVh-F | CTTCACGCTTGATGGCTACTG | *V. harveyi* |  |
|  | qVh-R | GTCACCCAATGCTACGACCT |  |  |
| *V. parahaemolyticus* (*tdh*) | qVp-F | GTAAAGGTCTCTGACTTTTGGAC | V. parahaemolyticus |  |
|  | qVp-R | TGGAATATGAACCTTCATCTTCACC |  |  |
| V. parahaemolyticus (*pirB*) | qPirB-F | GTGGGCTGATAACGACTC | V. parahaemolyticus (AHPND) | 187 |
|  | qPirB-R | ACCAACAGCAGGTGAATA |  |  |
