# Supplementary material for: Taurine metabolism is modulated in Vibrio-infected Penaeus vannamei to shape shrimp antibacterial response and survival
Source: Microbiome. 2022 Dec 5;10:213. doi: 10.1186/s40168-022-01414-9 (PMC9721036; doi:10.1186/s40168-022-01414-9)
Supplement: Supplementary file 4 — Additional file 3: Supplementary Table 2. Normalized area of metabolites in P. vannamei hepatopancreas. [file 40168_2022_1414_MOESM3_ESM.docx]

**Supplementary Table 2**. Normalized area of metabolites in *P. vannamei* hepatopancreas

| **No.** | **Category of metabolites** | **Metabolite name** | **Healthy** | **Diseased** | **Moribund** |
| --- | --- | --- | --- | --- | --- |
| 1 | amino acids and derivatives | Valine | 32931782.25 | 24629628.47 | 15797176.66 |
| 2 | amino acids and derivatives | Histamine | 765187.24 | 657814.30 | 162689.44 |
| 3 | amino acids and derivatives | Alanine | 37452863.54 | 41030896.89 | 52007298.09 |
| 4 | amino acids and derivatives | Glycine | 13490921.02 | 13624614.53 | 14209006.23 |
| 5 | amino acids and derivatives | Leucine | 1375552.80 | 840649.44 | 276659.74 |
| 6 | amino acids and derivatives | Proline | 80294671.34 | 82080029.03 | 68964036.21 |
| 7 | amino acids and derivatives | L-Isoleucine | 25269669.48 | 19997182.38 | 15391440.03 |
| 8 | amino acids and derivatives | Serine | 25107323.60 | 22610739.65 | 3659404.41 |
| 9 | amino acids and derivatives | Hydroxyproline | 54220578.05 | 61785619.19 | 43807710.12 |
| 10 | amino acids and derivatives | β-Alanine | 4268456.92 | 5617739.52 | 8333719.38 |
| 11 | amino acids and derivatives | L-Threonine | 21521754.03 | 18201340.59 | 9288875.43 |
| 12 | amino acids and derivatives | 5-hydroxy-Tryptophan | 59652.72 | 81220.35 | 56682.51 |
| 13 | amino acids and derivatives | Asparagine | 18388018.21 | 11316151.64 | 1606143.87 |
| 14 | amino acids and derivatives | Methionine | 10805878.31 | 7883954.61 | 4557106.58 |
| 15 | amino acids and derivatives | Aspartic acid | 5176202.50 | 9245214.97 | 1574699.51 |
| 16 | amino acids and derivatives | Cysteine | 4534203.92 | 5777284.28 | 683565.36 |
| 17 | amino acids and derivatives | L-Glutamine | 9099268.47 | 8578195.05 | 3039181.41 |
| 18 | amino acids and derivatives | Hypotaurine | 3380522.25 | 2749803.07 | 1468652.14 |
| 19 | amino acids and derivatives | Alanyl-alanine | 730276.76 | 849253.94 | 451107.14 |
| 20 | amino acids and derivatives | dl-Ornithine | 22231654.54 | 17049929.66 | 28335900.92 |
| 21 | amino acids and derivatives | Glutamic acid | 19959231.09 | 18298512.81 | 11073111.95 |
| 22 | amino acids and derivatives | Phenylalanine | 24802743.22 | 14988712.84 | 9421990.99 |
| 23 | amino acids and derivatives | Taurine | 35561168.64 | 44916324.09 | 24949510.73 |
| 24 | amino acids and derivatives | Lysine | 46544710.84 | 37116259.79 | 5583645.56 |
| 25 | amino acids and derivatives | N-Glycylglycine | 1845457.25 | 1396394.41 | 1822509.56 |
| 26 | amino acids and derivatives | Histidine | 8882232.44 | 7138273.75 | 4166820.15 |
| 27 | amino acids and derivatives | Tyrosine | 40288561.04 | 32674517.88 | 19342160.58 |
| 28 | amino acids and derivatives | Glycyl-proline | 1529992.55 | 882829.50 | 449592.41 |
| 29 | amino acids and derivatives | Tryptophan | 13498259.71 | 9562763.19 | 5448096.12 |
| 30 | amino acids and derivatives | Cystine | 6784582.95 | 5070190.58 | 540284.94 |
| 31 | amino acids and derivatives | Glycyl-phenylalanine | 526191.72 | 611289.13 | 682001.06 |
| 32 | amino acids and derivatives | L-Arginine | 52326477.79 | 63916626.92 | 158661750.53 |
| 33 | nucleic acids and derivatives | Uracil | 7107196.33 | 8215987.68 | 16997745.71 |
| 34 | nucleic acids and derivatives | Thymine | 4750972.85 | 5656141.07 | 8326196.74 |
| 35 | nucleic acids and derivatives | Hypoxanthine | 990136.85 | 1514720.74 | 14112642.55 |
| 36 | nucleic acids and derivatives | Xanthine | 1511496.17 | 2449768.11 | 13001320.97 |
| 37 | nucleic acids and derivatives | Uric acid | 2389539.44 | 5054184.53 | 9826788.86 |
| 38 | nucleic acids and derivatives | Uridine | 10594249.81 | 13458319.85 | 3722493.29 |
| 39 | nucleic acids and derivatives | Inosine | 21644389.69 | 30910043.05 | 17919803.88 |
| 40 | nucleic acids and derivatives | Guanosine | 3631984.12 | 4490950.42 | 2098589.60 |
| 41 | nucleic acids and derivatives | Deoxyguanosine | 3657251.54 | 4311828.32 | 2153594.61 |
| 42 | nucleic acids and derivatives | Guanine | 53858.04 | 111649.10 | 581376.98 |
| 43 | nucleic acids and derivatives | Ribose | 2143712.26 | 1551635.92 | 705921.72 |
| 44 | nucleic acids and derivatives | Valproic acid | 784886.73 | 706314.04 | 3388153.90 |
| 45 | nucleic acids and derivatives | Pyrimidine | 5221059.99 | 3654531.83 | 873479.32 |
| 46 | nucleic acids and derivatives | Pyrazine | 3878640.62 | 2775085.37 | 3033011.72 |
| 47 | nucleic acids and derivatives | Adenosine | 1570916.90 | 1131711.75 | 1540435.71 |
| 48 | nucleic acids and derivatives | Uridine-5'-monophosphate | 1390062.48 | 1705327.17 | 162102.42 |
| 49 | nucleic acids and derivatives | Adenosine-5'-monophosphate | 1775137.66 | 1879694.29 | 1026525.73 |
| 50 | fatty acids | Butanoic acid | 1170286.83 | 3025059.57 | 2182407.31 |
| 51 | fatty acids | Tetradecanoic acid | 3501539.64 | 2931105.08 | 1423512.80 |
| 52 | fatty acids | Hexadecanoic acid | 19091418.09 | 12644254.10 | 8725959.60 |
| 53 | fatty acids | Oleic Acid | 85985196.14 | 82081329.41 | 96305302.94 |
| 54 | fatty acids | Heptadecanoic acid | 1291437.51 | 1378445.36 | 2221652.32 |
| 55 | fatty acids | Eicosatetraenoic acid | 1138846.46 | 1369493.71 | 14619959.84 |
| 56 | fatty acids | Doconexent | 6732386.75 | 6448551.25 | 15404037.48 |
| 57 | fatty acids | Pentadecanoic acid | 526547.42 | 365603.18 | 588343.63 |
| 58 | fatty acids | Nonadecanoic acid | 216130.76 | 162674.59 | 199423.30 |
| 59 | fatty acids | Eicosan-1-ol | 135056.32 | 86830.39 | 122764.98 |
| 60 | fatty acids | Propanoic acid | 887653.26 | 874410.31 | 482570.79 |
| 61 | fatty acids | n-Dodecanoic acid | 444661.39 | 865035.27 | 1157429.55 |
| 62 | Organic acids and derivatives | Pyruvic acid | 3926751.44 | 5040914.97 | 8414345.21 |
| 63 | Organic acids and derivatives | Lactic acid | 7107521.56 | 8244675.53 | 51047997.35 |
| 64 | Organic acids and derivatives | Benzoic acid | 206380.91 | 203494.03 | 370014.40 |
| 65 | Organic acids and derivatives | Glyceric acid | 713891.14 | 522313.67 | 170441.05 |
| 66 | Organic acids and derivatives | Fumaric acid | 566202.14 | 435846.47 | 61621.64 |
| 67 | Organic acids and derivatives | Glyoxylic acid | 46478.01 | 123155.91 | 0.00 |
| 68 | Organic acids and derivatives | Malic acid | 3582570.48 | 4666355.88 | 1325773.15 |
| 69 | Organic acids and derivatives | Adipic acid | 1123048.61 | 1113983.18 | 1200901.21 |
| 70 | Organic acids and derivatives | Gluconic acid | 785016.38 | 950635.70 | 0.00 |
| 71 | Organic acids and derivatives | Citric acid | 1399182.74 | 787872.94 | 928749.11 |
| 72 | Organic acids and derivatives | Aminomalonic acid | 131148.81 | 108278.95 | 45171.83 |
| 73 | Organic acids and derivatives | 2-hydroxy-Glutaric acid | 1435358.19 | 625779.49 | 4967615.94 |
| 74 | carbohydrates | Pinitol | 11892875.49 | 12011024.38 | 3302606.53 |
| 75 | carbohydrates | Fructose | 1448468.82 | 1069656.06 | 738375.64 |
| 76 | carbohydrates | Galactose | 61744153.99 | 56978701.27 | 4445924.37 |
| 77 | carbohydrates | D-Allose | 17466117.38 | 15717212.61 | 2415358.87 |
| 78 | carbohydrates | Mannitol | 1025937.14 | 805002.50 | 216107.32 |
| 79 | carbohydrates | Viburnitol | 698252.77 | 807628.08 | 611533.52 |
| 80 | carbohydrates | Sequoyitol | 572388.46 | 1028581.66 | 628210.20 |
| 81 | carbohydrates | Ononitol | 330122.43 | 1125053.69 | 1000991.74 |
| 82 | carbohydrates | Cellobiose | 4065053.41 | 4112391.96 | 22860.39 |
| 83 | carbohydrates | D-Sucrose | 1750011.98 | 1654609.43 | 39744.59 |
| 84 | carbohydrates | Xylose | 2126358.74 | 1790080.02 | 825538.32 |
| 85 | carbohydrates | D-Glucose | 420687.26 | 0.00 | 459260.16 |
| 86 | hormone and others | Phenethylamine | 3134342.26 | 4282143.37 | 7859996.95 |
| 87 | hormone and others | cystathione | 2203630.98 | 1906465.89 | 823433.20 |
| 88 | hormone and others | Glycerophosphoglycerol | 582837.58 | 1475615.69 | 329378.73 |
| 89 | hormone and others | Neuraminic acid | 455758.40 | 434980.46 | 0.00 |
| 90 | hormone and others | Xylulose-5-phosphate | 300688.00 | 949929.96 | 3343288.16 |
| 91 | hormone and others | Ribulose-5-phosphate | 576536.79 | 947165.95 | 2377320.14 |
| 92 | hormone and others | Lyxonic acid | 719354.53 | 539613.77 | 257625.90 |
| 93 | hormone and others | Acetylneuraminic acid | 184400.26 | 164860.29 | 82484.48 |
| 94 | amine compound | Putrescine | 2769302.14 | 1542087.60 | 39591963.78 |
| 95 | amine compound | Ethanolaminephosphate | 2184543.60 | 3424067.51 | 1423000.41 |
| 96 | amine compound | Cadaverine | 2474161.02 | 2292605.35 | 40732275.40 |
| 97 | amine compound | Tryptamine | 127633.25 | 75385.25 | 228479.37 |
| 98 | amine compound | N-Acetyl-glucosamine | 4489256.42 | 6208646.02 | 1816050.14 |
| 99 | amine compound | Urea | 995244.93 | 1278029.14 | 35384.03 |
| 100 | coenzymes and vitamins | Niacin | 458079.72 | 777947.44 | 471049.64 |
| 101 | coenzymes and vitamins | Myo-Inositol | 7259503.44 | 7457169.93 | 13016875.35 |
| 102 | coenzymes and vitamins | γ-Tocopherol | 76916.32 | 112968.28 | 266633.35 |
| 103 | coenzymes and vitamins | α-Tocopherol | 114488.52 | 183980.84 | 367184.07 |
| 104 | coenzymes and vitamins | α-Tocopherolacetate | 99570.08 | 87413.00 | 16929.35 |
| 105 | lipid | Glycerol | 58428346.65 | 69089029.59 | 45122312.57 |
| 106 | lipid | Cholesterol | 16557731.58 | 29456611.55 | 47426520.84 |
| 107 | lipid | Campesterol | 478134.49 | 861374.60 | 536204.62 |
| 108 | lipid | β-Sitosterol | 2122854.94 | 2766011.66 | 1148382.38 |
