# Supplementary material for: Taurine metabolism is modulated in Vibrio-infected Penaeus vannamei to shape shrimp antibacterial response and survival
Source: Microbiome. 2022 Dec 5;10:213. doi: 10.1186/s40168-022-01414-9 (PMC9721036; doi:10.1186/s40168-022-01414-9)
Supplement: Supplementary file 5 — Additional file 4: Supplementary Table 3. Normalized area of metabolites in P. vannamei hepatopancreas. [file 40168_2022_1414_MOESM4_ESM.docx]

**Supplementary Table 3**. Normalized area of metabolites in *P. vannamei* hepatopancreas

| No. | Gene ID | saline | V. parahaemolyticus (AHPND) | taurine + V. parahaemolyticus (AHPND) | Transcripts | Pvalue (saline *VS* V. parahaemolyticus (AHPND)) | Pvalue(saline-vs-taurine + V. parahaemolyticus (AHPND)) | Pvalue(V. parahaemolyticus (AHPND)-vs-taurine + V. parahaemolyticus (AHPND)) |
| --- | --- | --- | --- | --- | --- | --- | --- | --- |
| 1 | LOC113800199 | 1.91 | 1.25 | 2.23 | XM_027350923.1 | 3.91E-02 | 6.46E-01 | 1.68E-01 |
| 2 | LOC113801935 | 6.06 | 2.89 | 4.55 | XM_027352358.1 | 2.71E-02 | 3.74E-01 | 4.89E-01 |
| 3 | LOC113804731 | 12.02 | 5.33 | 7.81 | XM_027355623.1 | 3.39E-02 | 1.18E-01 | 7.16E-01 |
| 4 | LOC113816554 | 0.10 | 0.04 | 0.29 | XM_027368593.1 | 2.89E-01 | 1.85E-01 | 1.86E-02 |
| 5 | LOC113823690 | 145.72 | 58.14 | 100.11 | XM_027376369.1 | 3.13E-05 | 4.33E-04 | 1.93E-01 |
| 6 | LOC113808398 | 23.85 | 18.58 | 20.52 | XM_027359812.1, BGI_novel_T000255, XM_027359818.1 | 3.77E-02 | 3.27E-02 | 9.07E-01 |
| 7 | LOC113814344 | 311.78 | 44.58 | 66.14 | BGI_novel_T004650, XM_027366397.1 | 3.81E-08 | 3.82E-06 | 3.93E-01 |
| 8 | LOC113812177 | 610.83 | 302.27 | 463.29 | XM_027364020.1, BGI_novel_T000367 | 1.36E-13 | 9.11E-03 | 2.59E-01 |
| 9 | LOC113805993 | 20.91 | 14.91 | 16.97 | XM_027357081.1 | 9.69E-02 | 4.36E-02 | 8.52E-01 |
| 10 | LOC113812913 | 37.68 | 14.54 | 24.14 | XM_027364835.1 | 4.95E-04 | 2.94E-03 | 3.43E-01 |
| 11 | LOC113819961 | 8.75 | 3.53 | 5.85 | XM_027372208.1 | 3.28E-03 | 9.59E-02 | 2.73E-01 |
| 12 | LOC113802766 | 147.96 | 71.27 | 71.77 | XM_027353383.1, BGI_novel_T001026, BGI_novel_T001025, XM_027353391.1 | 8.97E-02 | 2.56E-03 | 7.72E-01 |
| 13 | LOC113810046 | 82.85 | 54.59 | 75.24 | XM_027361730.1, BGI_novel_T003348 | 1.12E-02 | 7.50E-02 | 6.15E-01 |
| 14 | LOC113820410 | 199.28 | 171.63 | 191.69 | XM_027372740.1 | 4.45E-02 | 3.39E-02 | 8.01E-01 |
| 15 | LOC113806196 | 22.62 | 13.71 | 15.68 | BGI_novel_T002060, XM_027357319.1, XM_027357320.1 | 4.74E-02 | 5.97E-02 | 9.31E-01 |
| 16 | LOC113815316 | 109.92 | 30.94 | 79.43 | BGI_novel_T004894, XM_027367407.1 | 1.10E-05 | 8.79E-02 | 1.68E-02 |
| 17 | LOC113819808 | 54.11 | 42.28 | 47.91 | XM_027372007.1 | 2.30E-01 | 1.37E-03 | 6.67E-01 |
| 18 | LOC113811513 | 83.72 | 49.59 | 64.00 | XM_027363278.1, XM_027363280.1, BGI_novel_T003786, XM_027363281.1, XM_027363279.1 | 3.83E-03 | 1.26E-02 | 5.95E-01 |
| 19 | LOC113807596 | 2.36 | 0.48 | 1.43 | XM_027358891.1 | 7.48E-03 | 7.56E-02 | 1.60E-01 |
| 20 | LOC113816330 | 91.26 | 18.62 | 49.37 | XM_027368376.1 | 1.31E-08 | 3.15E-05 | 1.02E-02 |
| 21 | LOC113816325 | 6.91 | 2.34 | 3.09 | BGI_novel_T005201, XM_027368368.1 | 1.92E-02 | 5.99E-04 | 7.83E-01 |
| 22 | LOC113800171 | 105.67 | 56.62 | 102.81 | XM_027350896.1, BGI_novel_T009347 | 1.65E-02 | 3.24E-01 | 1.85E-01 |
| 23 | LOC113803075 | 10.96 | 7.57 | 9.71 | XM_027353775.1 | 8.86E-02 | 1.29E-02 | 6.89E-01 |
| 24 | LOC113826632 | 87.97 | 39.47 | 57.29 | XM_027379523.1, BGI_novel_T008187 | 3.59E-02 | 4.67E-03 | 5.79E-01 |
| 25 | LOC113808752 | 88.59 | 59.15 | 89.68 | XM_027360234.1 | 3.47E-02 | 2.16E-01 | 2.55E-01 |
| 26 | LOC113826872 | 8.96 | 6.00 | 9.41 | XM_027379772.1 | 4.86E-03 | 7.13E-02 | 9.63E-02 |
| 27 | LOC113818451 | 146.61 | 53.06 | 104.16 | XM_027370623.1, XM_027370622.1 | 3.47E-04 | 4.53E-02 | 4.32E-02 |
| 28 | LOC113829127 | 0.14 | 0.08 | 0.37 | XM_027382224.1, XM_027382223.1 | 2.91E-01 | 2.65E-01 | 4.24E-02 |
| 29 | LOC113829792 | 23.31 | 17.89 | 24.30 | XM_027382964.1 | 2.12E-03 | 1.24E-01 | 3.87E-01 |
| 30 | LOC113802667 | 29.37 | 22.39 | 30.35 | XM_027353272.1 | 1.64E-02 | 7.61E-02 | 5.34E-01 |
| 31 | LOC113823545 | 18.51 | 7.31 | 17.30 | XM_027376212.1 | 5.50E-05 | 1.98E-01 | 3.78E-02 |
| 32 | LOC113827362 | 8.76 | 4.96 | 6.31 | BGI_novel_T008339, XM_027380243.1 | 9.00E-02 | 2.76E-02 | 8.26E-01 |
| 33 | LOC113820096 | 34.95 | 18.46 | 47.02 | XM_027372358.1 | 8.54E-03 | 9.89E-01 | 2.45E-02 |
| 34 | LOC113804944 | 47.74 | 17.37 | 28.56 | XM_027355860.1, XM_027355859.1 | 4.30E-04 | 2.15E-02 | 2.84E-01 |
| 35 | LOC113817880 | 114.99 | 62.20 | 127.53 | BGI_novel_T005611, XM_027369993.1 | 2.73E-02 | 4.11E-01 | 1.51E-01 |
| 36 | LOC113824411 | 1.07 | 0.62 | 1.30 | XM_027377150.1 | 3.29E-02 | 5.67E-01 | 7.41E-02 |
| 37 | LOC113817851 | 7.83 | 5.32 | 5.32 | XM_027369954.1, BGI_novel_T005601, BGI_novel_T005600 | 1.62E-01 | 4.24E-02 | 7.20E-01 |
| 38 | LOC113827514 | 112.74 | 66.02 | 89.53 | XM_027380421.1, BGI_novel_T008409, BGI_novel_T008410 | 1.06E-02 | 8.99E-02 | 5.19E-01 |
| 39 | LOC113808565 | 33.21 | 23.65 | 31.48 | XM_027359996.1 | 3.02E-03 | 6.81E-02 | 4.46E-01 |
| 40 | LOC113817447 | 65.16 | 38.44 | 49.72 | XM_027369517.1, BGI_novel_T005496, BGI_novel_T005492, BGI_novel_T005497, BGI_novel_T005494, BGI_novel_T005495, BGI_novel_T005490, BGI_novel_T005491, BGI_novel_T005493 | 1.09E-03 | 1.01E-01 | 7.26E-01 |
| 41 | BGI_novel_G001018 | 0.08 | 0.00 | 1.02 | BGI_novel_T006109 | 4.07E-01 | 9.97E-03 | 4.58E-06 |
| 42 | LOC113814949 | 1.02 | 0.48 | 0.57 | XM_027367020.1 | 9.86E-02 | 4.11E-02 | 9.95E-01 |
| 43 | LOC113809327 | 79.30 | 51.92 | 69.64 | XM_027360887.1, XM_027360885.1, XM_027360886.1, XM_027360888.1 | 1.38E-03 | 1.51E-03 | 4.73E-01 |
| 44 | LOC113807601 | 10.38 | 8.13 | 8.23 | BGI_novel_T002524, XM_027358901.1 | 1.29E-01 | 1.28E-02 | 3.82E-01 |
| 45 | LOC113815070 | 6.16 | 5.26 | 6.04 | XM_027367166.1 | 1.42E-01 | 4.18E-02 | 6.85E-01 |
| 46 | LOC113825378 | 15.01 | 8.82 | 11.12 | XM_027378299.1, XM_027378229.1 | 2.77E-02 | 8.61E-02 | 6.92E-01 |
| 47 | LOC113819894 | 101.28 | 61.93 | 95.33 | XM_027372127.1 | 2.76E-02 | 1.99E-01 | 2.95E-01 |
| 48 | LOC113816684 | 3.73 | 0.82 | 3.06 | XM_027368745.1 | 8.44E-04 | 4.99E-02 | 5.59E-02 |
| 49 | LOC113819893 | 63.03 | 14.36 | 37.67 | XM_027372126.1 | 5.05E-04 | 1.03E-02 | 1.11E-01 |
| 50 | LOC113826359 | 187.90 | 50.75 | 131.63 | BGI_novel_T008077, XM_027379230.1 | 2.39E-02 | 6.84E-02 | 2.15E-01 |
| 51 | LOC113816912 | 0.88 | 0.53 | 1.93 | XM_027368901.1 | 1.01E-01 | 1.80E-01 | 1.46E-03 |
| 52 | LOC113815679 | 74.26 | 45.70 | 66.04 | BGI_novel_T004970, BGI_novel_T004971, BGI_novel_T004967, XM_027367707.1, BGI_novel_T004968, BGI_novel_T004969 | 4.51E-02 | 7.88E-02 | 4.23E-01 |
| 53 | LOC113815686 | 0.26 | 0.11 | 1.50 | XM_027367712.1 | 8.09E-02 | 1.70E-02 | 6.35E-04 |
| 54 | LOC113802453 | 80.78 | 25.40 | 70.51 | XM_027353045.1 | 4.05E-03 | 2.85E-01 | 8.41E-02 |
| 55 | LOC113802460 | 15.05 | 6.13 | 12.16 | BGI_novel_T009936, XM_027353056.1 | 2.54E-02 | 1.94E-01 | 2.87E-01 |
| 56 | LOC113827416 | 44.68 | 14.35 | 14.51 | XM_027380298.1 | 2.45E-05 | 7.94E-05 | 6.85E-01 |
| 57 | LOC113800316 | 272.82 | 213.35 | 894.11 | XM_027351067.1, BGI_novel_T009419 | 3.57E-01 | 9.03E-02 | 1.91E-02 |
| 58 | LOC113805335 | 18.15 | 10.76 | 16.61 | XM_027356323.1, XM_027356325.1 | 3.67E-06 | 1.34E-02 | 1.81E-01 |
| 59 | LOC113807231 | 31.73 | 10.85 | 11.52 | BGI_novel_T002402, XM_027358445.1 | 5.37E-02 | 2.90E-05 | 7.32E-01 |
| 60 | LOC113819630 | 31.89 | 8.67 | 27.06 | XM_027371841.1 | 1.89E-02 | 3.85E-01 | 1.31E-01 |
| 61 | BGI_novel_G000461 | 323.58 | 85.76 | 103.90 | BGI_novel_T002762 | 1.08E-02 | 1.82E-03 | 8.74E-01 |
| 62 | LOC113812271 | 6.59 | 1.59 | 2.57 | XM_027364132.1 | 7.21E-07 | 3.94E-08 | 2.91E-01 |
| 63 | LOC113822744 | 330.51 | 199.88 | 454.17 | BGI_novel_T006935, BGI_novel_T006937, XM_027375281.1, BGI_novel_T006936, XM_027375283.1, XM_027375280.1, XM_027375282.1, XM_027375284.1 | 1.93E-02 | 9.50E-01 | 3.82E-02 |
| 64 | LOC113828400 | 3.89 | 1.72 | 5.87 | BGI_novel_T008655, BGI_novel_T008656, XM_027381367.1, BGI_novel_T008654 | 1.88E-01 | 6.11E-01 | 3.38E-02 |
| 65 | LOC113804272 | 6.52 | 5.08 | 7.51 | XM_027355123.1, XM_027355124.1, XM_027355128.1, XM_027355126.1, XM_027355127.1, XM_027355122.1, XM_027355125.1 | 6.78E-03 | 1.39E-01 | 7.76E-02 |
| 66 | LOC113814355 | 24.87 | 13.83 | 31.27 | XM_027366408.1 | 4.21E-03 | 6.45E-01 | 1.15E-01 |
| 67 | BGI_novel_G000822 | 4.11 | 0.80 | 1.10 | BGI_novel_T004988 | 1.15E-02 | 8.68E-05 | 9.60E-01 |
| 68 | LOC113819352 | 42.43 | 26.53 | 43.11 | XM_027371590.1 | 8.58E-03 | 2.71E-01 | 3.11E-01 |
| 69 | LOC113821646 | 81.65 | 44.94 | 74.60 | XM_027374156.1 | 1.28E-02 | 4.14E-02 | 2.39E-01 |
| 70 | LOC113826787 | 38.12 | 24.16 | 38.81 | XM_027379675.1 | 3.42E-03 | 1.11E-01 | 1.27E-01 |
| 71 | LOC113827332 | 137.79 | 107.46 | 156.75 | BGI_novel_T008330, XM_027380217.1 | 4.42E-02 | 2.81E-01 | 2.44E-01 |
| 72 | LOC113813430 | 56.57 | 49.51 | 73.85 | XM_027365408.1 | 2.09E-02 | 6.77E-01 | 3.20E-01 |
| 73 | LOC113818487 | 11.97 | 8.59 | 9.73 | XM_027370678.1, XM_027370679.1 | 5.20E-02 | 1.36E-02 | 9.11E-01 |
| 74 | LOC113826365 | 9.43 | 8.82 | 9.09 | XM_027379241.1 | 3.14E-01 | 4.97E-02 | 5.41E-01 |
| 75 | LOC113829568 | 1.17 | 0.57 | 0.65 | XM_027382760.1 | 4.92E-02 | 8.80E-03 | 8.36E-01 |
| 76 | LOC113815957 | 56.77 | 34.81 | 51.65 | XM_027367971.1, XM_027367973.1, XM_027367968.1, BGI_novel_T005060, BGI_novel_T005063, BGI_novel_T005067, BGI_novel_T005064, BGI_novel_T005065, BGI_novel_T005061, BGI_novel_T005066, XM_027367974.1, XM_027367972.1, XM_027367969.1, BGI_novel_T005062, XM_027367970.1 | 4.10E-02 | 1.54E-01 | 5.35E-01 |
| 77 | LOC113807917 | 4.77 | 3.69 | 5.02 | XM_027359253.1, XM_027359245.1 | 4.06E-02 | 9.90E-02 | 5.18E-01 |
| 78 | LOC113815940 | 0.43 | 0.17 | 1.32 | XM_027367955.1 | 2.03E-02 | 3.05E-01 | 4.34E-02 |
| 79 | LOC113802710 | 8.00 | 2.65 | 3.72 | BGI_novel_T010005, BGI_novel_T010004, XM_027353324.1 | 5.79E-04 | 1.75E-04 | 5.09E-01 |
| 80 | LOC113802711 | 63.31 | 17.93 | 34.37 | BGI_novel_T010006, XM_027353325.1, BGI_novel_T010007 | 2.39E-03 | 3.92E-06 | 2.66E-01 |
| 81 | LOC113809384 | 0.95 | 0.59 | 0.79 | XM_027360959.1 | 3.57E-02 | 8.26E-02 | 7.07E-01 |
| 82 | LOC113809672 | 160.42 | 80.68 | 99.18 | XM_027361330.1, XM_027361332.1, BGI_novel_T003263, BGI_novel_T003268, BGI_novel_T003267, BGI_novel_T003266, BGI_novel_T003265, BGI_novel_T003264, BGI_novel_T003262 | 2.79E-02 | 2.21E-03 | 9.42E-01 |
| 83 | LOC113812215 | 10.73 | 5.99 | 8.97 | XM_027364069.1 | 2.99E-02 | 9.13E-02 | 4.24E-01 |
| 84 | LOC113815550 | 68.10 | 22.28 | 39.08 | BGI_novel_T000445, BGI_novel_T000446, BGI_novel_T000442, BGI_novel_T000439, BGI_novel_T000447, BGI_novel_T000440, XM_027367611.1, BGI_novel_T000441 | 3.25E-03 | 8.09E-02 | 3.37E-01 |
| 85 | LOC113815570 | 37.52 | 11.41 | 19.75 | XM_027367633.1, BGI_novel_T000443, BGI_novel_T000444, BGI_novel_T000449, BGI_novel_T000448, BGI_novel_T000450 | 2.17E-05 | 4.59E-05 | 2.49E-01 |
| 86 | LOC113821379 | 29.02 | 9.63 | 18.92 | BGI_novel_T006609, BGI_novel_T006610, XM_027373856.1 | 2.04E-03 | 6.11E-02 | 2.36E-01 |
| 87 | LOC113821820 | 2.95 | 2.75 | 2.83 | BGI_novel_T000648, XM_027374344.1 | 4.53E-01 | 3.63E-02 | 5.76E-01 |
| 88 | BGI_novel_G001167 | 0.06 | 0.00 | 0.24 | BGI_novel_T007081 | 2.18E-01 | 3.46E-01 | 9.58E-05 |
| 89 | LOC113822324 | 1.16 | 0.78 | 1.83 | XM_027374858.1, BGI_novel_T006839, BGI_novel_T006840 | 5.26E-02 | 5.29E-01 | 1.14E-02 |
| 90 | BGI_novel_G000643 | 1.34 | 0.05 | 0.97 | BGI_novel_T003887 | 7.05E-03 | 5.06E-01 | 5.39E-03 |
| 91 | LOC113800264 | 0.78 | 0.04 | 3.52 | BGI_novel_T009364, XM_027351008.1, BGI_novel_T009365 | 3.49E-02 | 3.98E-01 | 1.14E-01 |
| 92 | LOC113800245 | 1.06 | 0.05 | 2.16 | XM_027350991.1, XM_027350990.1, XM_027350989.1 | 2.57E-07 | 3.90E-01 | 1.35E-07 |
| 93 | LOC113810063 | 1.15 | 0.59 | 1.20 | XM_027361751.1 | 2.00E-03 | 2.57E-01 | 1.90E-02 |
| 94 | LOC113806687 | 1.79 | 1.34 | 3.47 | XM_027357851.1, BGI_novel_T002198 | 6.64E-02 | 4.22E-01 | 4.50E-02 |
| 95 | LOC113813176 | 2.95 | 1.36 | 2.09 | XM_027365131.1 | 4.73E-04 | 9.51E-02 | 3.69E-01 |
| 96 | LOC113816128 | 7.61 | 5.18 | 8.20 | XM_027368330.1, XM_027368260.1, XM_027368191.1, XM_027368398.1 | 2.60E-02 | 2.54E-01 | 2.46E-01 |
| 97 | LOC113803079 | 6.08 | 2.35 | 4.86 | BGI_novel_T001075, XM_027353780.1, XM_027353781.1 | 1.20E-06 | 1.43E-02 | 4.91E-02 |
| 98 | LOC113809969 | 0.75 | 0.14 | 1.04 | XM_027361650.1 | 4.74E-02 | 9.04E-01 | 2.29E-02 |
| 99 | LOC113801166 | 350.98 | 155.47 | 205.01 | XM_027351980.1 | 8.35E-04 | 7.39E-04 | 8.27E-01 |
| 100 | LOC113821236 | 161.71 | 20.04 | 25.63 | XM_027373724.1 | 4.23E-06 | 5.22E-07 | 9.40E-01 |
| 101 | LOC113800662 | 1.86 | 0.00 | 4.19 | XM_027351470.1 | 4.62E-02 | 6.75E-01 | 1.85E-03 |
| 102 | LOC113823540 | 12.73 | 9.59 | 10.59 | XM_027376208.1 | 3.28E-02 | 9.31E-03 | 5.76E-01 |
| 103 | LOC113821223 | 37.56 | 14.64 | 21.57 | XM_027373705.1, XM_027373713.1 | 1.68E-02 | 4.96E-02 | 5.31E-01 |
| 104 | LOC113812552 | 82.70 | 21.03 | 65.17 | BGI_novel_T004130, BGI_novel_T004127, BGI_novel_T004129, BGI_novel_T004131, XM_027364457.1, BGI_novel_T004128, BGI_novel_T004132 | 4.10E-06 | 4.77E-01 | 7.13E-02 |
| 105 | LOC113817634 | 540.17 | 292.02 | 357.88 | BGI_novel_T005533, XM_027369688.1, BGI_novel_T005534 | 2.56E-04 | 6.34E-03 | 8.23E-01 |
| 106 | LOC113821256 | 163.45 | 76.56 | 101.80 | BGI_novel_T000623, BGI_novel_T000625, BGI_novel_T000624, XM_027373748.1 | 3.74E-02 | 1.02E-01 | 6.45E-01 |
| 107 | LOC113822637 | 169.30 | 94.76 | 130.89 | XM_027375170.1, XM_027375169.1 | 9.44E-03 | 5.25E-03 | 5.71E-01 |
| 108 | LOC113828161 | 2.07 | 1.58 | 5.46 | BGI_novel_T000908, BGI_novel_T000909, XM_027381105.1, XM_027381098.1 | 2.68E-01 | 1.07E-01 | 1.90E-02 |
| 109 | LOC113829084 | 297.08 | 144.18 | 182.59 | XM_027382177.1, XM_027382186.1 | 2.11E-02 | 1.11E-03 | 7.87E-01 |
| 110 | LOC113817770 | 176.66 | 59.66 | 120.21 | BGI_novel_T005580, XM_027369863.1 | 1.22E-06 | 1.80E-02 | 7.39E-02 |
| 111 | LOC113825151 | 29.26 | 17.97 | 24.68 | XM_027377948.1 | 3.68E-03 | 4.32E-03 | 4.15E-01 |
| 112 | LOC113812171 | 0.67 | 0.19 | 0.37 | XM_027364010.1 | 2.56E-02 | 1.31E-01 | 4.57E-01 |
| 113 | LOC113823461 | 1.41 | 0.83 | 0.93 | XM_027376116.1 | 7.56E-04 | 8.24E-03 | 9.39E-01 |
| 114 | LOC113800812 | 87.81 | 51.22 | 83.89 | XM_027351601.1, BGI_novel_T009560 | 1.29E-04 | 1.73E-01 | 1.81E-01 |
| 115 | LOC113824173 | 0.61 | 0.09 | 0.43 | XM_027376921.1, BGI_novel_T007423 | 6.09E-05 | 1.42E-01 | 2.25E-02 |
| 116 | LOC113819666 | 121.63 | 33.06 | 76.52 | XM_027371865.1 | 4.85E-04 | 5.50E-04 | 8.92E-02 |
| 117 | LOC113825393 | 68.89 | 36.74 | 55.79 | XM_027378214.1, BGI_novel_T007769 | 5.58E-04 | 4.05E-02 | 2.08E-01 |
| 118 | LOC113814736 | 31.16 | 16.02 | 20.78 | XM_027366793.1 | 1.11E-03 | 2.07E-02 | 6.94E-01 |
| 119 | LOC113806188 | 53.62 | 13.08 | 28.61 | XM_027357310.1, BGI_novel_T002059, BGI_novel_T002058 | 1.29E-08 | 1.12E-05 | 2.82E-02 |
| 120 | LOC113800373 | 300.57 | 228.89 | 346.50 | BGI_novel_T009435, XM_027351140.1, BGI_novel_T009436, BGI_novel_T009434, BGI_novel_T009437 | 4.72E-02 | 8.88E-01 | 3.95E-01 |
| 121 | LOC113829448 | 1.73 | 1.11 | 1.76 | XM_027382621.1 | 4.54E-02 | 9.85E-02 | 3.79E-01 |
| 122 | LOC113819701 | 2.87 | 1.68 | 1.85 | BGI_novel_T006111, XM_027371899.1 | 4.97E-02 | 3.50E-03 | 8.81E-01 |
| 123 | LOC113811831 | 6.57 | 4.48 | 6.15 | XM_027363650.1 | 1.79E-02 | 4.33E-02 | 6.10E-01 |
| 124 | LOC113805442 | 11.00 | 9.81 | 11.54 | XM_027356429.1, XM_027356428.1, BGI_novel_T001766 | 7.90E-02 | 3.23E-02 | 8.43E-01 |
| 125 | LOC113806422 | 1.54 | 1.07 | 1.47 | XM_027357534.1 | 1.44E-02 | 3.86E-02 | 5.44E-01 |
| 126 | LOC113808559 | 2.40 | 1.93 | 2.18 | XM_027359988.1 | 4.97E-02 | 5.34E-02 | 8.83E-01 |
| 127 | LOC113811764 | 3.97 | 2.59 | 3.81 | XM_027363576.1, XM_027363578.1, XM_027363580.1, XM_027363579.1, XM_027363577.1 | 4.13E-02 | 1.09E-01 | 4.59E-01 |
| 128 | LOC113828991 | 3.90 | 2.74 | 7.49 | XM_027382061.1 | 7.83E-02 | 2.26E-01 | 2.37E-02 |
| 129 | BGI_novel_G001438 | 0.25 | 0.00 | 0.12 | BGI_novel_T008708 | 4.81E-02 | 5.56E-01 | 3.13E-01 |
| 130 | LOC113802883 | 1.86 | 1.18 | 1.22 | XM_027353534.1, BGI_novel_T001032 | 3.52E-01 | 2.56E-02 | 7.79E-01 |
| 131 | LOC113830427 | 21.40 | 5.06 | 8.82 | XM_027383641.1 | 1.62E-03 | 9.29E-03 | 2.75E-01 |
| 132 | LOC113802181 | 134.31 | 94.60 | 119.68 | XM_027352708.1 | 9.73E-04 | 5.49E-02 | 7.03E-01 |
| 133 | LOC113814203 | 56.61 | 40.10 | 51.56 | XM_027366235.1 | 2.60E-02 | 3.90E-02 | 6.87E-01 |
| 134 | LOC113828551 | 47.01 | 24.76 | 31.27 | BGI_novel_T008692, XM_027381553.1 | 7.22E-05 | 2.87E-03 | 9.02E-01 |
| 135 | BGI_novel_G000740 | 153.57 | 116.20 | 132.38 | BGI_novel_T004596 | 2.70E-02 | 4.76E-02 | 8.99E-01 |
| 136 | LOC113804783 | 30.06 | 14.31 | 23.31 | XM_027355684.1, XM_027355692.1 | 5.24E-04 | 3.00E-01 | 4.11E-01 |
| 137 | LOC113825596 | 5.71 | 2.93 | 6.37 | XM_027378434.1, BGI_novel_T007860 | 3.02E-02 | 5.76E-01 | 5.52E-02 |
| 138 | LOC113827846 | 302.26 | 139.61 | 161.37 | XM_027380780.1, XM_027380781.1 | 5.18E-02 | 6.35E-03 | 9.84E-01 |
| 139 | LOC113813749 | 0.82 | 0.23 | 0.38 | XM_027365808.1 | 2.96E-02 | 1.12E-01 | 7.13E-01 |
| 140 | BGI_novel_G000725 | 1.47 | 0.40 | 0.54 | BGI_novel_T004444 | 1.68E-02 | 1.40E-01 | 9.37E-01 |
| 141 | LOC113813745 | 7.77 | 3.37 | 12.54 | XM_027365802.1, XM_027365801.1, BGI_novel_T004443, BGI_novel_T004445 | 7.62E-02 | 6.98E-01 | 1.77E-02 |
| 142 | LOC113813746 | 21.94 | 12.19 | 15.72 | BGI_novel_T004449, XM_027365805.1, BGI_novel_T004450, BGI_novel_T004447, XM_027365804.1, XM_027365803.1 | 8.96E-04 | 1.06E-03 | 7.35E-01 |
| 143 | LOC113808076 | 11.51 | 5.53 | 7.72 | XM_027359395.1, BGI_novel_T002730 | 9.97E-03 | 6.72E-03 | 5.53E-01 |
| 144 | LOC113808603 | 10.74 | 5.22 | 8.30 | XM_027360041.1, BGI_novel_T002866, XM_027360042.1, BGI_novel_T002865 | 2.21E-04 | 8.55E-02 | 1.88E-01 |
| 145 | LOC113813750 | 2.34 | 0.09 | 1.37 | BGI_novel_T004451, BGI_novel_T004448, XM_027365809.1 | 5.83E-04 | 2.43E-01 | 8.91E-02 |
| 146 | LOC113815045 | 14.28 | 10.01 | 15.43 | XM_027367134.1 | 3.41E-02 | 2.64E-01 | 2.12E-01 |
| 147 | LOC113819867 | 249.83 | 93.84 | 184.98 | XM_027372089.1 | 5.50E-03 | 7.04E-03 | 1.66E-01 |
| 148 | LOC113818808 | 0.64 | 0.33 | 0.62 | XM_027371000.1 | 7.36E-03 | 1.81E-01 | 2.37E-01 |
| 149 | LOC113820851 | 5.83 | 2.06 | 6.23 | BGI_novel_T006442, BGI_novel_T006441, BGI_novel_T006440, XM_027373228.1 | 6.36E-03 | 3.05E-01 | 4.50E-02 |
| 150 | LOC113819977 | 19.58 | 2.10 | 2.19 | XM_027372225.1, XM_027372224.1 | 1.01E-03 | 1.37E-04 | 9.02E-01 |
| 151 | LOC113812397 | 148.02 | 77.32 | 101.73 | XM_027364268.1 | 2.24E-02 | 4.45E-03 | 6.47E-01 |
| 152 | LOC113807175 | 5.07 | 0.47 | 2.25 | XM_027358390.1, BGI_novel_T002388 | 1.25E-02 | 4.32E-01 | 1.63E-01 |
| 153 | LOC113823443 | 2.57 | 1.08 | 2.93 | XM_027376084.1 | 2.71E-03 | 6.41E-01 | 3.58E-02 |
| 154 | LOC113813342 | 144.55 | 74.65 | 100.98 | BGI_novel_T004325, XM_027365317.1 | 3.50E-02 | 2.41E-02 | 7.31E-01 |
| 155 | LOC113826969 | 213.98 | 6.16 | 16.86 | XM_027379865.1 | 5.97E-07 | 5.01E-05 | 5.19E-02 |
| 156 | BGI_novel_G001382 | 148.09 | 2.04 | 9.53 | BGI_novel_T008257 | 2.47E-09 | 2.33E-05 | 4.44E-04 |
| 157 | LOC113816062 | 145.91 | 97.42 | 104.41 | BGI_novel_T005094, XM_027368084.1, BGI_novel_T005095 | 4.48E-02 | 4.76E-04 | 6.24E-01 |
| 158 | LOC113819684 | 0.17 | 0.01 | 0.87 | XM_027371879.1 | 7.82E-02 | 4.85E-01 | 3.02E-02 |
| 159 | LOC113820860 | 8.17 | 4.65 | 8.31 | XM_027373239.1, XM_027373240.1 | 2.04E-02 | 5.84E-01 | 1.42E-01 |
| 160 | LOC113810336 | 114.60 | 92.13 | 115.47 | BGI_novel_T003433, XM_027362017.1, XM_027362018.1, XM_027362016.1 | 3.31E-02 | 5.03E-02 | 7.88E-01 |
| 161 | LOC113813421 | 55.36 | 21.56 | 30.55 | XM_027365392.1, BGI_novel_T004355 | 5.39E-03 | 2.74E-02 | 5.24E-01 |
| 162 | BGI_novel_G000159 | 0.08 | 0.03 | 0.75 | BGI_novel_T001101 | 4.60E-01 | 6.43E-02 | 1.34E-02 |
| 163 | LOC113816682 | 6.35 | 1.24 | 2.21 | XM_027368744.1 | 1.01E-03 | 1.46E-02 | 5.39E-01 |
| 164 | LOC113817487 | 167.56 | 28.18 | 81.34 | XM_027369556.1 | 5.25E-07 | 2.93E-02 | 4.86E-02 |
| 165 | LOC113824334 | 1.06 | 0.09 | 0.52 | XM_027377083.1 | 8.99E-03 | 3.01E-01 | 4.52E-03 |
| 166 | LOC113824803 | 0.25 | 0.08 | 0.35 | XM_027377596.1 | 2.48E-02 | 8.90E-01 | 1.09E-01 |
| 167 | BGI_novel_G000427 | 11.14 | 5.65 | 9.99 | BGI_novel_T002506 | 1.55E-02 | 1.10E-01 | 1.64E-01 |
| 168 | BGI_novel_G000428 | 3.58 | 1.16 | 2.07 | BGI_novel_T002507 | 3.02E-02 | 1.44E-01 | 6.62E-01 |
| 169 | LOC113824571 | 25.74 | 11.98 | 20.58 | XM_027377315.1, BGI_novel_T007516 | 1.54E-06 | 1.94E-03 | 1.33E-01 |
| 170 | LOC113810627 | 4.03 | 3.01 | 4.45 | XM_027362252.1, XM_027362253.1, BGI_novel_T003523, XM_027362251.1, BGI_novel_T003526, XM_027362254.1, BGI_novel_T003525, XM_027362248.1, BGI_novel_T003527, XM_027362255.1, XM_027362250.1, BGI_novel_T003524 | 1.35E-02 | 2.16E-01 | 4.81E-01 |
| 171 | LOC113809619 | 691.30 | 186.32 | 329.75 | BGI_novel_T008211, XM_027361261.1, BGI_novel_T008210 | 3.97E-02 | 1.41E-01 | 5.23E-01 |
| 172 | LOC113813369 | 23.82 | 2.03 | 7.14 | XM_027365344.1 | 1.95E-06 | 1.48E-02 | 2.18E-01 |
| 173 | LOC113811921 | 5.09 | 0.18 | 3.81 | XM_027363776.1 | 5.84E-05 | 4.12E-01 | 2.40E-02 |
| 174 | LOC113824913 | 0.10 | 0.09 | 0.43 | XM_027377681.1 | 7.99E-01 | 3.07E-02 | 2.80E-02 |
| 175 | BGI_novel_G000261 | 33.18 | 4.45 | 6.57 | BGI_novel_T001629 | 5.23E-04 | 4.15E-04 | 7.84E-01 |
| 176 | BGI_novel_G000906 | 0.47 | 0.25 | 2.53 | BGI_novel_T005433 | 3.80E-01 | 2.17E-01 | 4.14E-02 |
| 177 | LOC113800111 | 141.14 | 52.93 | 171.19 | BGI_novel_T009329, XM_027350839.1 | 4.25E-02 | 9.43E-01 | 1.73E-04 |
| 178 | LOC113822241 | 4.25 | 3.87 | 12.67 | BGI_novel_T006825, BGI_novel_T006826, BGI_novel_T006827, XM_027374780.1 | 8.10E-01 | 9.48E-05 | 9.09E-04 |
| 179 | LOC113815446 | 71.75 | 1.48 | 79.12 | BGI_novel_T004914, BGI_novel_T004916, XM_027367514.1, BGI_novel_T004915 | 7.86E-03 | 7.23E-01 | 1.08E-01 |
| 180 | LOC113810786 | 156.74 | 113.86 | 173.39 | BGI_novel_T003594, BGI_novel_T003596, XM_027362429.1, BGI_novel_T003595, XM_027362430.1 | 7.46E-03 | 4.58E-01 | 1.73E-01 |
| 181 | LOC113809383 | 22.63 | 13.85 | 21.62 | XM_027360978.1, XM_027360970.1, XM_027360962.1 | 3.01E-03 | 8.20E-02 | 2.10E-02 |
| 182 | LOC113828353 | 41.89 | 26.99 | 31.50 | BGI_novel_T008638, BGI_novel_T008635, BGI_novel_T008634, BGI_novel_T008636, BGI_novel_T008637, BGI_novel_T008633, XM_027381298.1 | 4.71E-03 | 7.34E-03 | 9.23E-01 |
| 183 | LOC113821065 | 11.08 | 8.63 | 11.42 | XM_027373492.1, BGI_novel_T006495 | 4.43E-02 | 8.11E-02 | 4.18E-01 |
| 184 | LOC113827051 | 18.50 | 11.45 | 13.99 | BGI_novel_T008261, XM_027379946.1 | 1.83E-02 | 2.40E-02 | 8.28E-01 |
| 185 | LOC113812444 | 1.59 | 0.55 | 1.16 | XM_027364331.1, XM_027364330.1 | 1.00E-02 | 1.84E-01 | 2.38E-01 |
| 186 | LOC113825543 | 80.13 | 23.01 | 38.16 | BGI_novel_T007845, XM_027378383.1, BGI_novel_T007849 | 3.37E-03 | 3.93E-03 | 6.78E-01 |
| 187 | LOC113825544 | 17.69 | 8.49 | 9.03 | BGI_novel_T007830, BGI_novel_T007832, XM_027378385.1, XM_027378384.1 | 5.28E-02 | 5.00E-02 | 7.37E-01 |
| 188 | LOC113825546 | 185.22 | 54.51 | 71.20 | BGI_novel_T007840, BGI_novel_T007833, BGI_novel_T007846, BGI_novel_T007828, BGI_novel_T007844, BGI_novel_T007838, BGI_novel_T007842, BGI_novel_T007850, BGI_novel_T007831, BGI_novel_T007836, BGI_novel_T007834, BGI_novel_T007837, BGI_novel_T007843, BGI_novel_T007829, BGI_novel_T007827, BGI_novel_T007847, BGI_novel_T007835, BGI_novel_T007848, BGI_novel_T007841, BGI_novel_T007839, BGI_novel_T007826, XM_027378389.1 | 2.01E-04 | 2.83E-04 | 8.17E-01 |
| 189 | LOC113814676 | 133.35 | 66.37 | 94.37 | XM_027366729.1 | 3.27E-03 | 1.41E-02 | 5.27E-01 |
| 190 | LOC113806992 | 3.92 | 2.88 | 3.76 | XM_027358123.1 | 4.26E-02 | 2.50E-02 | 8.23E-01 |
| 191 | LOC113827616 | 26.81 | 9.94 | 22.21 | XM_027380508.1 | 1.88E-04 | 2.00E-01 | 3.02E-02 |
| 192 | LOC113827622 | 345.16 | 196.57 | 266.72 | XM_027380521.1 | 5.37E-03 | 3.31E-01 | 4.71E-01 |
| 193 | LOC113827638 | 538.93 | 352.07 | 447.05 | XM_027380539.1 | 4.46E-03 | 9.99E-02 | 6.25E-01 |
| 194 | BGI_novel_G000591 | 3.15 | 1.33 | 2.74 | BGI_novel_T003598 | 8.47E-03 | 1.10E-01 | 2.20E-01 |
| 195 | LOC113816135 | 21.61 | 12.71 | 24.47 | BGI_novel_T005127, BGI_novel_T005129, XM_027368168.1, BGI_novel_T005128 | 2.25E-03 | 1.73E-01 | 1.76E-02 |
| 196 | BGI_novel_G001317 | 520.53 | 278.21 | 392.16 | BGI_novel_T007920 | 1.70E-02 | 1.82E-01 | 5.18E-01 |
| 197 | LOC113815555 | 4428.75 | 2058.05 | 2263.33 | BGI_novel_T000713, BGI_novel_T000714, XM_027367615.1 | 3.32E-02 | 1.06E-01 | 9.14E-01 |
| 198 | LOC113815565 | 20688.58 | 9955.24 | 18122.44 | BGI_novel_T004958, BGI_novel_T004950, BGI_novel_T004946, XM_027367624.1, BGI_novel_T004956, BGI_novel_T004957, BGI_novel_T004955, BGI_novel_T004951 | 9.34E-03 | 6.57E-01 | 1.79E-01 |
| 199 | LOC113815556 | 142.80 | 71.84 | 103.42 | BGI_novel_T004937, BGI_novel_T004952, BGI_novel_T004954, BGI_novel_T004953, XM_027367616.1 | 2.12E-02 | 2.55E-01 | 4.74E-01 |
| 200 | LOC113815561 | 59769.36 | 23252.66 | 44036.00 | XM_027367621.1, BGI_novel_T004949, BGI_novel_T004948, BGI_novel_T004938, BGI_novel_T004947 | 3.64E-03 | 3.09E-01 | 2.36E-01 |
| 201 | LOC113815562 | 472.94 | 74.56 | 182.76 | BGI_novel_T004944, XM_027367622.1, BGI_novel_T004941, BGI_novel_T004940, BGI_novel_T004945, BGI_novel_T004939, BGI_novel_T004943, BGI_novel_T004942 | 3.26E-05 | 7.39E-02 | 1.16E-01 |
| 202 | LOC113823738 | 41336.35 | 16174.76 | 25633.36 | XM_027376457.1, XM_027376443.1, XM_027376450.1, BGI_novel_T000719, BGI_novel_T000716, BGI_novel_T000721, BGI_novel_T000717, BGI_novel_T000715, XM_027376436.1, XM_027376430.1, BGI_novel_T000720, BGI_novel_T000718 | 1.95E-02 | 1.17E-01 | 4.92E-01 |
| 203 | LOC113815560 | 467.34 | 71.59 | 382.89 | XM_027367620.1 | 8.76E-05 | 4.90E-01 | 1.03E-02 |
| 204 | LOC113805758 | 135.66 | 0.20 | 117.73 | XM_027356816.1 | 2.00E-02 | 8.99E-01 | 3.61E-02 |
| 205 | LOC113823795 | 1064.53 | 45.78 | 1251.14 | XM_027376486.1 | 1.10E-11 | 8.99E-01 | 1.93E-02 |
| 206 | LOC113811621 | 2.31 | 1.20 | 1.81 | XM_027363402.1 | 1.57E-02 | 4.31E-02 | 6.07E-01 |
| 207 | LOC113805736 | 19624.27 | 12691.97 | 17205.27 | BGI_novel_T001884, BGI_novel_T001902, BGI_novel_T001895, BGI_novel_T001887, BGI_novel_T001880, BGI_novel_T001883, BGI_novel_T001894, BGI_novel_T001885, BGI_novel_T001889, BGI_novel_T001901, XM_027356789.1, BGI_novel_T001892, BGI_novel_T001899, BGI_novel_T001903, BGI_novel_T001886, BGI_novel_T001893, BGI_novel_T001905, BGI_novel_T001904, BGI_novel_T001897, BGI_novel_T001900, BGI_novel_T001906 | 4.69E-02 | 3.97E-01 | 5.45E-01 |
| 208 | LOC113817089 | 1028.86 | 446.31 | 746.26 | XM_027369089.1, BGI_novel_T005369 | 2.87E-03 | 2.76E-01 | 3.19E-01 |
| 209 | LOC113808173 | 105.68 | 73.84 | 85.54 | XM_027359505.1 | 9.76E-02 | 1.48E-02 | 7.65E-01 |
| 210 | LOC113800294 | 0.74 | 0.55 | 0.91 | XM_027351043.1, XM_027351044.1, XM_027351042.1 | 4.77E-02 | 4.84E-01 | 2.75E-01 |
| 211 | LOC113802673 | 5.87 | 2.70 | 4.88 | XM_027353278.1, BGI_novel_T009966 | 2.44E-02 | 1.63E-01 | 2.65E-01 |
| 212 | LOC113812241 | 159.34 | 64.00 | 115.29 | XM_027364097.1 | 2.15E-03 | 1.31E-01 | 1.58E-01 |
| 213 | LOC113814629 | 7.97 | 4.00 | 8.46 | XM_027366678.1 | 4.35E-05 | 3.72E-01 | 2.31E-02 |
| 214 | LOC113803630 | 2.23 | 0.71 | 2.18 | XM_027354430.1 | 1.42E-02 | 1.38E-01 | 9.19E-02 |
| 215 | LOC113807492 | 8.95 | 3.60 | 3.86 | XM_027358756.1 | 6.52E-03 | 9.24E-03 | 9.37E-01 |
| 216 | LOC113807491 | 0.49 | 0.36 | 0.80 | XM_027358754.1, XM_027358753.1, XM_027358755.1 | 1.51E-01 | 5.50E-01 | 1.52E-02 |
| 217 | LOC113823150 | 0.63 | 0.51 | 1.18 | XM_027375767.1 | 5.62E-01 | 3.85E-01 | 3.73E-02 |
| 218 | LOC113803114 | 8.50 | 6.82 | 7.31 | BGI_novel_T001082, XM_027353829.1 | 1.16E-01 | 4.45E-02 | 5.61E-01 |
| 219 | LOC113813365 | 4.44 | 1.34 | 2.33 | XM_027365343.1 | 1.31E-08 | 7.19E-07 | 1.31E-01 |
| 220 | LOC113805315 | 4.18 | 2.62 | 4.02 | XM_027356309.1 | 3.90E-02 | 7.55E-02 | 4.23E-01 |
| 221 | LOC113829271 | 33.99 | 20.19 | 29.68 | XM_027382388.1 | 3.94E-04 | 5.67E-02 | 4.36E-01 |
| 222 | LOC113815479 | 0.03 | 0.00 | 0.18 | XM_027367543.1 | 3.81E-01 | 1.71E-01 | 3.46E-02 |
| 223 | BGI_novel_G001234 | 95.95 | 32.46 | 52.00 | BGI_novel_T007436 | 2.43E-02 | 1.43E-01 | 4.92E-01 |
| 224 | BGI_novel_G001235 | 28.89 | 13.87 | 66.03 | BGI_novel_T007437 | 4.12E-02 | 5.29E-01 | 3.67E-02 |
| 225 | LOC113829842 | 10.10 | 5.00 | 8.10 | XM_027383019.1, BGI_novel_T009095, BGI_novel_T009096 | 9.76E-03 | 2.37E-02 | 4.82E-01 |
| 226 | LOC113808891 | 7.01 | 2.69 | 2.98 | XM_027360399.1, XM_027360407.1 | 5.07E-02 | 2.17E-02 | 5.63E-01 |
| 227 | LOC113821498 | 84.72 | 68.53 | 85.55 | XM_027374006.1 | 2.32E-02 | 6.19E-02 | 8.25E-01 |
| 228 | LOC113820796 | 9.29 | 5.88 | 8.43 | XM_027373157.1 | 1.12E-02 | 9.22E-02 | 3.48E-01 |
| 229 | LOC113825617 | 1.21 | 0.61 | 0.68 | XM_027378460.1 | 3.06E-02 | 2.81E-02 | 8.73E-01 |
| 230 | LOC113829804 | 42.28 | 33.08 | 39.35 | XM_027382977.1 | 1.79E-01 | 2.51E-02 | 9.38E-01 |
| 231 | BGI_novel_G001483 | 15.36 | 8.56 | 13.89 | BGI_novel_T009079 | 1.06E-03 | 9.76E-02 | 1.36E-01 |
| 232 | LOC113800280 | 45.78 | 12.08 | 24.17 | XM_027351026.1, BGI_novel_T009383 | 8.36E-12 | 3.19E-04 | 1.61E-02 |
| 233 | LOC113804808 | 37.38 | 26.93 | 38.78 | XM_027355716.1 | 8.61E-03 | 1.88E-01 | 3.12E-01 |
| 234 | LOC113815228 | 129.03 | 84.66 | 103.48 | XM_027367292.1, XM_027367291.1 | 6.14E-03 | 2.96E-03 | 8.21E-01 |
| 235 | LOC113810130 | 21.29 | 14.42 | 19.31 | XM_027361834.1 | 1.95E-02 | 2.11E-03 | 5.38E-01 |
| 236 | LOC113828392 | 683.36 | 404.24 | 727.38 | XM_027381357.1, XM_027381358.1, BGI_novel_T008650, XM_027381354.1, BGI_novel_T008651, XM_027381356.1, XM_027381355.1 | 3.81E-02 | 5.16E-01 | 2.20E-01 |
| 237 | LOC113813301 | 3.18 | 1.57 | 2.77 | XM_027365280.1 | 1.30E-02 | 8.22E-03 | 2.13E-01 |
| 238 | LOC113802806 | 0.24 | 0.18 | 0.87 | XM_027353433.1 | 4.48E-01 | 3.39E-02 | 4.81E-04 |
| 239 | LOC113802801 | 75.09 | 55.02 | 59.21 | XM_027353423.1, XM_027353420.1, XM_027353424.1, BGI_novel_T010035, BGI_novel_T010039, BGI_novel_T010031, BGI_novel_T010034, XM_027353418.1, BGI_novel_T010038, XM_027353425.1, BGI_novel_T010033, BGI_novel_T010037, XM_027353421.1, XM_027353426.1, BGI_novel_T010032, BGI_novel_T010036, XM_027353422.1, BGI_novel_T010030 | 1.20E-04 | 2.85E-03 | 6.13E-01 |
| 240 | LOC113809365 | 219.15 | 92.51 | 153.47 | XM_027360936.1 | 2.50E-06 | 2.15E-02 | 3.32E-01 |
| 241 | LOC113813895 | 25.95 | 10.24 | 31.14 | XM_027365949.1 | 7.13E-04 | 8.16E-01 | 1.43E-02 |
| 242 | LOC113819304 | 16.54 | 13.67 | 18.85 | BGI_novel_T005991, XM_027371540.1 | 3.07E-02 | 9.42E-02 | 5.81E-01 |
| 243 | BGI_novel_G001216 | 5.72 | 3.38 | 4.09 | BGI_novel_T007344 | 3.37E-03 | 4.23E-01 | 9.79E-01 |
| 244 | BGI_novel_G001217 | 5.72 | 3.38 | 5.81 | BGI_novel_T007345 | 3.37E-03 | 1.00E-01 | 7.21E-02 |
| 245 | LOC113804690 | 27.59 | 14.21 | 31.86 | XM_027355574.1 | 7.03E-03 | 7.84E-01 | 1.50E-01 |
| 246 | LOC113810111 | 30.73 | 16.44 | 25.23 | XM_027361794.1 | 1.01E-02 | 1.10E-02 | 3.19E-01 |
| 247 | LOC113826393 | 4.05 | 1.76 | 2.60 | XM_027379277.1 | 3.32E-04 | 2.66E-02 | 3.78E-01 |
| 248 | LOC113804700 | 36.33 | 23.87 | 42.53 | BGI_novel_T000156, BGI_novel_T000158, XM_027355591.1, BGI_novel_T000157, BGI_novel_T000160, BGI_novel_T000159, BGI_novel_T000155 | 7.77E-03 | 8.30E-01 | 1.19E-01 |
| 249 | LOC113811726 | 54.13 | 8.11 | 32.79 | XM_027363520.1, XM_027363519.1 | 1.34E-06 | 1.61E-05 | 2.29E-03 |
| 250 | LOC113823144 | 519.82 | 405.36 | 538.98 | XM_027375761.1 | 3.48E-03 | 4.00E-01 | 5.64E-01 |
| 251 | LOC113815268 | 26.48 | 4.17 | 20.01 | XM_027367356.1 | 6.71E-10 | 2.11E-01 | 4.00E-03 |
| 252 | LOC113802814 | 11.51 | 8.07 | 8.84 | BGI_novel_T010048, BGI_novel_T010047, XM_027353441.1 | 1.75E-01 | 1.22E-03 | 7.99E-01 |
| 253 | LOC113807294 | 3.85 | 2.89 | 4.08 | XM_027358528.1 | 2.50E-02 | 1.54E-01 | 2.92E-01 |
| 254 | LOC113824940 | 15.34 | 10.78 | 13.05 | XM_027377708.1 | 8.89E-03 | 2.77E-02 | 8.30E-01 |
| 255 | LOC113803489 | 76.30 | 60.83 | 67.66 | XM_027354275.1 | 2.53E-02 | 2.12E-02 | 7.15E-01 |
| 256 | LOC113801067 | 187.06 | 146.20 | 154.60 | XM_027351876.1 | 2.44E-02 | 1.81E-02 | 6.19E-01 |
| 257 | LOC113821387 | 152.96 | 118.69 | 139.20 | XM_027373863.1, XM_027373862.1 | 2.56E-02 | 1.00E-01 | 9.17E-01 |
| 258 | LOC113823756 | 24.43 | 17.04 | 24.06 | XM_027376444.1 | 2.33E-03 | 1.31E-01 | 3.62E-01 |
| 259 | LOC113814574 | 0.61 | 0.51 | 1.02 | XM_027366624.1, BGI_novel_T004712 | 2.63E-01 | 3.07E-01 | 1.14E-02 |
| 260 | LOC113820973 | 28.60 | 8.72 | 16.90 | BGI_novel_T006474, BGI_novel_T006476, BGI_novel_T006471, BGI_novel_T006477, BGI_novel_T006470, BGI_novel_T006472, BGI_novel_T006480, BGI_novel_T006479, XM_027373388.1, BGI_novel_T006475, BGI_novel_T006473, BGI_novel_T006478 | 1.41E-04 | 1.52E-02 | 1.34E-01 |
| 261 | LOC113813536 | 0.13 | 0.00 | 0.34 | XM_027365537.1, XM_027365536.1 | 4.98E-02 | 5.28E-01 | 4.58E-02 |
| 262 | LOC113815986 | 50.33 | 39.45 | 54.16 | XM_027368002.1, XM_027368003.1, XM_027368008.1, XM_027368009.1, XM_027368005.1, XM_027368011.1, XM_027368010.1, XM_027368006.1, XM_027368007.1, XM_027368004.1 | 1.89E-02 | 2.97E-01 | 4.15E-01 |
| 263 | LOC113814959 | 1.84 | 1.16 | 3.82 | XM_027367039.1 | 2.07E-01 | 2.23E-01 | 4.30E-02 |
| 264 | LOC113801070 | 17.13 | 8.04 | 15.10 | BGI_novel_T009621, BGI_novel_T009620, XM_027351885.1 | 1.71E-04 | 2.17E-03 | 4.00E-02 |
| 265 | LOC113810753 | 90.04 | 68.74 | 76.25 | XM_027362400.1 | 5.80E-02 | 1.34E-02 | 6.23E-01 |
| 266 | LOC113809218 | 1.97 | 1.27 | 3.23 | XM_027360747.1 | 1.00E-01 | 6.14E-01 | 4.58E-02 |
| 267 | LOC113825773 | 0.65 | 0.11 | 0.18 | XM_027378605.1 | 2.14E-02 | 5.86E-02 | 5.69E-01 |
| 268 | BGI_novel_G001242 | 0.78 | 0.12 | 0.45 | BGI_novel_T007483 | 2.36E-03 | 1.41E-01 | 1.85E-01 |
| 269 | LOC113803563 | 2.37 | 2.31 | 4.13 | XM_027354354.1 | 5.52E-01 | 1.96E-01 | 4.26E-02 |
| 270 | LOC113802681 | 2.48 | 1.69 | 2.27 | XM_027353288.1 | 6.14E-03 | 1.98E-04 | 5.03E-01 |
| 271 | LOC113819177 | 0.30 | 0.08 | 0.24 | XM_027371417.1 | 3.77E-03 | 2.36E-01 | 2.39E-01 |
| 272 | LOC113813502 | 6.81 | 3.90 | 6.63 | XM_027365499.1 | 8.03E-04 | 2.18E-01 | 1.88E-01 |
| 273 | LOC113827080 | 6.44 | 5.47 | 6.38 | BGI_novel_T008264, XM_027379974.1 | 2.26E-01 | 1.91E-02 | 7.76E-01 |
| 274 | LOC113806193 | 0.05 | 0.02 | 0.14 | XM_027357314.1 | 4.01E-01 | 3.02E-01 | 2.46E-02 |
| 275 | LOC113814979 | 0.73 | 0.00 | 0.90 | XM_027367054.1 | 1.54E-03 | 8.54E-01 | 3.58E-02 |
| 276 | LOC113826304 | 3.00 | 0.58 | 1.13 | XM_027379168.1 | 6.02E-05 | 5.94E-05 | 2.61E-01 |
| 277 | LOC113819856 | 1.00 | 0.48 | 1.40 | XM_027372069.1, BGI_novel_T006139, XM_027372067.1, XM_027372070.1 | 1.55E-02 | 8.47E-01 | 1.05E-02 |
| 278 | LOC113808718 | 15.55 | 6.89 | 8.60 | XM_027360191.1, BGI_novel_T002905, BGI_novel_T002904, BGI_novel_T002903, BGI_novel_T002906 | 4.51E-04 | 3.30E-02 | 5.76E-01 |
| 279 | LOC113818571 | 1.45 | 0.98 | 1.86 | XM_027370771.1, XM_027370770.1, XM_027370769.1 | 5.60E-02 | 6.60E-01 | 4.89E-02 |
| 280 | LOC113825153 | 1.69 | 0.46 | 0.54 | BGI_novel_T007707, XM_027377951.1 | 9.35E-03 | 4.38E-03 | 7.62E-01 |
| 281 | LOC113820496 | 2.75 | 2.12 | 3.46 | XM_027372877.1, BGI_novel_T000600, XM_027372855.1, XM_027372872.1, XM_027372859.1, XM_027372866.1 | 1.54E-02 | 4.94E-01 | 1.28E-01 |
| 282 | LOC113813817 | 64.99 | 18.65 | 72.31 | XM_027365869.1 | 1.22E-07 | 9.41E-01 | 2.88E-02 |
| 283 | LOC113810219 | 633.19 | 344.43 | 384.58 | XM_027361933.1 | 7.43E-03 | 5.58E-02 | 9.70E-01 |
| 284 | BGI_novel_G001611 | 1.10 | 0.09 | 0.76 | BGI_novel_T009678 | 4.79E-02 | 4.06E-01 | 1.89E-01 |
| 285 | LOC113824544 | 0.47 | 0.30 | 0.47 | XM_027377284.1, BGI_novel_T007505 | 4.51E-02 | 1.73E-01 | 3.80E-01 |
| 286 | LOC113802220 | 1206.28 | 905.52 | 1278.92 | XM_027352755.1 | 2.38E-02 | 2.97E-01 | 3.94E-01 |
| 287 | LOC113802776 | 1.35 | 0.09 | 0.64 | XM_027353392.1 | 2.73E-04 | 1.58E-01 | 8.96E-02 |
| 288 | BGI_novel_G000220 | 32.40 | 28.93 | 31.47 | BGI_novel_T001358 | 4.12E-01 | 4.14E-02 | 8.19E-01 |
| 289 | LOC113803289 | 61.75 | 45.04 | 89.68 | XM_027354037.1, XM_027354039.1, XM_027354038.1 | 9.00E-05 | 7.68E-01 | 7.50E-03 |
| 290 | LOC113813626 | 3.88 | 1.91 | 3.71 | XM_027365652.1 | 8.02E-07 | 1.51E-01 | 1.35E-01 |
| 291 | BGI_novel_G001343 | 226.86 | 165.82 | 379.90 | BGI_novel_T008010 | 2.64E-01 | 4.94E-01 | 6.48E-03 |
| 292 | BGI_novel_G001636 | 0.18 | 0.07 | 1.35 | BGI_novel_T009877 | 3.85E-02 | 4.74E-03 | 3.08E-05 |
| 293 | LOC113821685 | 4.44 | 3.19 | 4.93 | XM_027374199.1, XM_027374198.1, BGI_novel_T006679 | 1.12E-02 | 2.18E-01 | 1.61E-01 |
| 294 | LOC113805477 | 39.75 | 19.20 | 32.17 | XM_027356481.1 | 4.03E-04 | 4.68E-02 | 2.51E-01 |
| 295 | LOC113812272 | 0.94 | 0.65 | 1.38 | XM_027364135.1, XM_027364133.1, XM_027364134.1 | 2.35E-02 | 9.80E-01 | 2.47E-02 |
| 296 | LOC113803760 | 6.72 | 4.58 | 5.96 | XM_027354584.1, BGI_novel_T001262 | 4.12E-02 | 7.08E-02 | 6.04E-01 |
| 297 | LOC113824601 | 4.63 | 2.02 | 2.73 | XM_027377357.1 | 4.00E-02 | 6.38E-02 | 6.84E-01 |
| 298 | LOC113829896 | 301.76 | 104.83 | 210.08 | BGI_novel_T000960, XM_027383071.1, BGI_novel_T000959, XM_027383088.1, BGI_novel_T000961, XM_027383079.1, BGI_novel_T000962 | 1.23E-02 | 7.11E-02 | 2.51E-01 |
| 299 | LOC113812901 | 38.59 | 18.51 | 22.56 | XM_027364827.1 | 7.84E-02 | 4.72E-03 | 9.59E-01 |
| 300 | BGI_novel_G001013 | 0.78 | 0.00 | 0.35 | BGI_novel_T006062 | 1.44E-02 | 4.43E-01 | 2.09E-01 |
| 301 | LOC113829072 | 0.19 | 0.14 | 1.68 | XM_027382161.1 | 5.86E-01 | 7.64E-03 | 4.73E-03 |
| 302 | BGI_novel_G001378 | 0.21 | 0.14 | 1.57 | BGI_novel_T008246 | 5.27E-01 | 1.25E-01 | 3.50E-02 |
| 303 | LOC113822757 | 0.58 | 0.29 | 6.43 | BGI_novel_T006939, BGI_novel_T006940, BGI_novel_T006941, XM_027375296.1 | 3.02E-01 | 5.67E-03 | 3.67E-04 |
| 304 | LOC113802559 | 56.66 | 14.84 | 120.12 | XM_027353163.1 | 3.85E-02 | 6.24E-01 | 2.46E-02 |
| 305 | LOC113823959 | 0.12 | 0.04 | 0.34 | XM_027376695.1 | 2.74E-01 | 2.88E-01 | 4.66E-02 |
| 306 | LOC113809249 | 78.90 | 34.78 | 237.61 | XM_027360791.1, BGI_novel_T003132, BGI_novel_T003133 | 7.15E-02 | 5.57E-01 | 2.29E-02 |
| 307 | LOC113825517 | 44.25 | 35.47 | 38.10 | XM_027378358.1 | 6.62E-02 | 2.45E-02 | 6.04E-01 |
| 308 | LOC113823596 | 25.68 | 14.46 | 25.88 | XM_027376276.1 | 6.98E-03 | 1.04E-01 | 1.90E-01 |
| 309 | LOC113812879 | 1.00 | 0.80 | 1.45 | XM_027364798.1, XM_027364797.1 | 8.37E-02 | 7.94E-01 | 2.93E-02 |
| 310 | LOC113826613 | 0.09 | 0.00 | 0.07 | XM_027379503.1 | 2.75E-02 | 4.85E-01 | 2.94E-01 |
| 311 | BGI_novel_G001266 | 2.69 | 1.02 | 1.84 | BGI_novel_T007677 | 2.20E-02 | 1.28E-01 | 8.80E-02 |
| 312 | LOC113811454 | 0.64 | 0.37 | 0.48 | XM_027363192.1 | 1.74E-03 | 2.34E-02 | 8.34E-01 |
| 313 | LOC113805950 | 35.54 | 27.99 | 50.58 | XM_027357034.1, BGI_novel_T001963, XM_027357032.1, XM_027357035.1, XM_027357033.1 | 4.16E-03 | 9.50E-01 | 4.14E-02 |
| 314 | LOC113800559 | 0.40 | 0.17 | 0.39 | XM_027351349.1 | 4.45E-02 | 4.31E-01 | 3.10E-01 |
| 315 | LOC113821018 | 4.31 | 2.93 | 6.01 | XM_027373450.1, XM_027373451.1, XM_027373449.1 | 2.81E-01 | 8.04E-01 | 4.68E-02 |
| 316 | LOC113827272 | 141.91 | 117.39 | 133.81 | XM_027380170.1 | 7.72E-02 | 1.94E-03 | 7.42E-01 |
| 317 | LOC113812472 | 2.24 | 1.77 | 2.76 | XM_027364375.1, BGI_novel_T001105 | 1.41E-02 | 8.85E-01 | 2.24E-02 |
| 318 | LOC113826837 | 6.64 | 5.55 | 7.86 | XM_027379733.1 | 3.08E-02 | 3.63E-01 | 2.44E-01 |
| 319 | LOC113804256 | 0.98 | 0.49 | 0.83 | XM_027355102.1, BGI_novel_T001402 | 3.58E-02 | 2.17E-01 | 2.81E-01 |
| 320 | LOC113821366 | 3.88 | 2.48 | 4.46 | XM_027373841.1 | 2.78E-03 | 2.25E-01 | 3.48E-02 |
| 321 | LOC113828262 | 1.80 | 0.97 | 2.14 | XM_027381195.1 | 3.61E-02 | 6.32E-01 | 1.31E-01 |
| 322 | LOC113813777 | 39.93 | 17.82 | 29.56 | XM_027365837.1 | 1.06E-05 | 2.87E-02 | 1.66E-01 |
| 323 | LOC113821002 | 25.15 | 9.62 | 21.23 | XM_027373426.1, XM_027373425.1 | 2.70E-02 | 1.77E-02 | 2.33E-01 |
| 324 | LOC113810419 | 62.37 | 45.62 | 47.73 | BGI_novel_T003472, XM_027362107.1, XM_027362106.1, BGI_novel_T003473 | 7.72E-02 | 2.56E-04 | 4.35E-01 |
| 325 | LOC113813776 | 14.48 | 5.62 | 7.96 | XM_027365836.1 | 4.49E-02 | 1.77E-02 | 5.85E-01 |
| 326 | LOC113824450 | 1.02 | 0.78 | 0.97 | XM_027377189.1 | 2.62E-02 | 1.23E-01 | 8.06E-01 |
| 327 | LOC113829697 | 0.77 | 0.09 | 1.87 | XM_027382893.1 | 2.38E-02 | 4.95E-01 | 1.64E-02 |
| 328 | LOC113823550 | 433.05 | 279.55 | 325.97 | BGI_novel_T007204, XM_027376216.1, BGI_novel_T007205, XM_027376215.1 | 9.18E-05 | 2.70E-03 | 9.84E-01 |
| 329 | LOC113821846 | 2.15 | 1.38 | 2.15 | XM_027374367.1, XM_027374368.1, XM_027374371.1, BGI_novel_T006703, XM_027374372.1, BGI_novel_T006702, XM_027374369.1 | 4.71E-02 | 4.49E-01 | 2.71E-01 |
| 330 | LOC113807514 | 1.55 | 1.10 | 1.31 | XM_027358784.1 | 1.60E-01 | 4.98E-03 | 8.37E-01 |
| 331 | LOC113808650 | 0.33 | 0.06 | 0.08 | BGI_novel_T002883, XM_027360110.1 | 5.28E-02 | 1.69E-02 | 8.95E-01 |
| 332 | LOC113814279 | 0.06 | 0.00 | 0.22 | XM_027366320.1 | 4.28E-02 | 5.23E-01 | 7.36E-02 |
| 333 | LOC113810750 | 0.10 | 0.04 | 0.85 | XM_027362397.1, XM_027362396.1 | 5.24E-01 | 5.79E-02 | 6.24E-03 |
| 334 | LOC113810749 | 0.14 | 0.00 | 0.07 | XM_027362395.1 | 1.88E-02 | 2.90E-01 | 2.59E-01 |
| 335 | LOC113827140 | 158.87 | 42.66 | 63.03 | BGI_novel_T006726, XM_027380038.1 | 8.31E-03 | 8.88E-05 | 5.46E-01 |
| 336 | LOC113812820 | 1.68 | 0.55 | 0.82 | XM_027364739.1 | 3.17E-02 | 1.28E-01 | 5.43E-01 |
| 337 | BGI_novel_G000694 | 1.42 | 0.35 | 0.63 | BGI_novel_T004193 | 6.41E-06 | 5.55E-03 | 4.61E-01 |
| 338 | LOC113805112 | 0.18 | 0.05 | 0.15 | XM_027356036.1 | 1.58E-02 | 6.07E-01 | 5.68E-02 |
| 339 | LOC113815969 | 0.02 | 0.00 | 0.08 | XM_027367986.1 | 2.42E-01 | 3.00E-01 | 1.06E-02 |
| 340 | LOC113807409 | 1.29 | 0.64 | 1.62 | BGI_novel_T002439, BGI_novel_T002440, XM_027358655.1 | 4.10E-03 | 5.49E-01 | 9.38E-03 |
| 341 | LOC113808875 | 0.06 | 0.02 | 1.15 | XM_027360377.1 | 2.87E-01 | 4.76E-02 | 7.63E-03 |
| 342 | LOC113821258 | 0.13 | 0.13 | 0.41 | XM_027373747.1, XM_027373749.1 | 7.44E-01 | 2.93E-02 | 3.66E-02 |
| 343 | BGI_novel_G001291 | 0.38 | 0.00 | 0.08 | BGI_novel_T007796 | 4.76E-02 | 1.32E-01 | 6.67E-01 |
| 344 | LOC113818538 | 37.37 | 1.44 | 47.27 | XM_027370724.1 | 1.64E-04 | 7.80E-01 | 1.02E-03 |
| 345 | BGI_novel_G000719 | 3.87 | 1.43 | 2.71 | BGI_novel_T004402 | 4.35E-02 | 1.39E-01 | 3.95E-01 |
| 346 | LOC113822400 | 4.50 | 3.33 | 3.90 | XM_027374933.1, XM_027374937.1, BGI_novel_T006857, XM_027374936.1, XM_027374932.1, XM_027374931.1, BGI_novel_T006858, XM_027374935.1 | 5.55E-03 | 9.35E-04 | 8.15E-01 |
| 347 | LOC113816986 | 97.58 | 61.13 | 71.06 | BGI_novel_T005337, BGI_novel_T005338, BGI_novel_T005339, XM_027368979.1 | 7.27E-02 | 2.04E-02 | 8.07E-01 |
| 348 | LOC113808859 | 0.30 | 0.17 | 0.56 | XM_027360356.1 | 9.56E-02 | 3.97E-01 | 1.48E-02 |
| 349 | LOC113802652 | 0.15 | 0.01 | 0.19 | XM_027353254.1 | 4.61E-02 | 8.35E-01 | 8.70E-02 |
| 350 | LOC113805171 | 0.25 | 0.03 | 0.32 | XM_027356122.1 | 3.58E-03 | 8.03E-01 | 4.61E-02 |
| 351 | LOC113826078 | 19.85 | 4.83 | 110.74 | XM_027378956.1 | 3.24E-02 | 8.94E-02 | 9.99E-04 |
| 352 | LOC113808604 | 1.15 | 0.67 | 1.54 | BGI_novel_T002869, BGI_novel_T002868, BGI_novel_T002867, XM_027360043.1 | 2.93E-02 | 7.05E-01 | 1.46E-01 |
| 353 | LOC113799907 | 0.11 | 0.09 | 0.66 | XM_027350610.1 | 9.02E-01 | 3.43E-02 | 5.87E-02 |
| 354 | LOC113814637 | 0.03 | 0.01 | 0.26 | XM_027366685.1 | 5.24E-01 | 1.58E-01 | 2.13E-02 |
| 355 | LOC113811739 | 7.13 | 4.27 | 5.05 | XM_027363534.1, XM_027363535.1, XM_027363536.1, XM_027363539.1, XM_027363537.1, XM_027363533.1 | 2.28E-02 | 1.65E-04 | 7.92E-01 |
| 356 | LOC113816960 | 1.69 | 1.20 | 4.95 | XM_027368952.1 | 5.63E-01 | 2.59E-02 | 1.10E-01 |
| 357 | LOC113800443 | 0.29 | 0.08 | 0.31 | XM_027351216.1 | 4.21E-04 | 4.07E-01 | 5.98E-03 |
| 358 | LOC113802658 | 32.40 | 25.47 | 34.42 | XM_027353262.1, XM_027353260.1, BGI_novel_T009964, XM_027353261.1 | 4.48E-02 | 1.95E-01 | 5.44E-01 |
| 359 | LOC113828230 | 2.95 | 0.81 | 4.06 | BGI_novel_T008612, XM_027381161.1, XM_027381159.1, XM_027381160.1, BGI_novel_T008613, XM_027381162.1 | 6.08E-05 | 7.41E-01 | 2.67E-02 |
| 360 | LOC113823945 | 0.23 | 0.10 | 1.01 | XM_027376677.1 | 3.90E-01 | 3.44E-02 | 1.32E-03 |
| 361 | LOC113805826 | 15.05 | 12.10 | 15.53 | XM_027356876.1, XM_027356877.1, XM_027356880.1, BGI_novel_T001930, BGI_novel_T001927, XM_027356875.1, XM_027356878.1, BGI_novel_T001931, BGI_novel_T001928, BGI_novel_T001929, XM_027356881.1 | 4.17E-02 | 9.19E-02 | 5.01E-01 |
| 362 | LOC113814443 | 0.99 | 0.61 | 1.84 | XM_027366473.1, XM_027366475.1 | 6.50E-02 | 2.83E-01 | 7.69E-03 |
| 363 | LOC113808921 | 0.85 | 0.79 | 2.01 | BGI_novel_T002977, XM_027360425.1 | 4.78E-01 | 8.18E-02 | 4.09E-02 |
| 364 | LOC113801903 | 7.96 | 5.77 | 6.73 | XM_027352316.1, BGI_novel_T009707, XM_027352315.1, XM_027352317.1 | 9.60E-02 | 1.26E-02 | 9.07E-01 |
| 365 | LOC113821011 | 11.21 | 7.05 | 10.36 | BGI_novel_T006485, XM_027373438.1 | 2.72E-02 | 3.78E-02 | 4.98E-01 |
| 366 | LOC113807417 | 13.95 | 7.63 | 11.00 | BGI_novel_T002442, BGI_novel_T002443, XM_027358665.1 | 1.23E-03 | 1.37E-06 | 6.06E-01 |
| 367 | LOC113802455 | 1.87 | 0.52 | 2.32 | XM_027353048.1 | 2.68E-08 | 6.99E-01 | 3.55E-07 |
| 368 | LOC113806678 | 15.49 | 10.11 | 15.45 | BGI_novel_T002193, XM_027357843.1, BGI_novel_T002194 | 5.62E-03 | 4.42E-02 | 3.82E-01 |
| 369 | LOC113812264 | 2.52 | 1.96 | 2.71 | BGI_novel_T004047, XM_027364125.1, BGI_novel_T004048 | 3.66E-03 | 1.27E-01 | 5.50E-01 |
| 370 | LOC113810712 | 13.51 | 12.91 | 14.65 | XM_027362345.1 | 1.86E-01 | 4.22E-02 | 4.61E-01 |
| 371 | LOC113814567 | 0.32 | 0.15 | 0.68 | XM_027366615.1 | 1.02E-01 | 5.25E-01 | 1.93E-02 |
| 372 | LOC113829394 | 25.62 | 18.86 | 22.66 | XM_027382566.1, XM_027382565.1, BGI_novel_T009012, XM_027382567.1, XM_027382564.1, XM_027382563.1 | 4.05E-04 | 1.08E-04 | 9.67E-01 |
| 373 | LOC113820408 | 0.04 | 0.00 | 0.05 | XM_027372738.1 | 7.78E-02 | 9.63E-01 | 4.58E-02 |
| 374 | LOC113814824 | 8.43 | 6.18 | 11.27 | XM_027366882.1 | 2.69E-02 | 9.82E-01 | 4.14E-02 |
| 375 | LOC113806371 | 0.25 | 0.00 | 0.16 | XM_027357497.1 | 4.05E-02 | 6.53E-01 | 9.37E-02 |
| 376 | LOC113811330 | 2.92 | 2.11 | 4.89 | BGI_novel_T003751, BGI_novel_T003752, XM_027363059.1 | 1.39E-01 | 4.77E-01 | 2.92E-02 |
| 377 | LOC113818823 | 38.05 | 34.87 | 46.34 | XM_027371016.1, BGI_novel_T005847, XM_027371014.1, XM_027371015.1 | 3.44E-02 | 1.47E-01 | 2.39E-01 |
| 378 | LOC113801825 | 17.83 | 7.00 | 34.63 | BGI_novel_T009701, BGI_novel_T009700, XM_027352254.1, BGI_novel_T009699 | 3.06E-02 | 8.05E-01 | 5.51E-02 |
| 379 | LOC113817300 | 0.42 | 0.27 | 0.43 | XM_027369325.1 | 2.66E-02 | 1.54E-01 | 3.38E-01 |
| 380 | LOC113810910 | 0.94 | 0.13 | 2.02 | BGI_novel_T003662, BGI_novel_T003665, XM_027362562.1, XM_027362565.1, BGI_novel_T003663, XM_027362563.1, BGI_novel_T003661, XM_027362564.1, BGI_novel_T003664 | 4.67E-03 | 8.39E-01 | 2.41E-02 |
| 381 | LOC113809208 | 78.44 | 71.03 | 76.17 | XM_027360739.1, BGI_novel_T003090 | 2.09E-01 | 1.04E-02 | 6.43E-01 |
| 382 | LOC113802189 | 0.11 | 0.02 | 0.02 | BGI_novel_T001015, XM_027352721.1 | 1.28E-02 | 3.92E-02 | 8.16E-01 |
| 383 | LOC113819960 | 77.25 | 33.57 | 42.20 | XM_027372206.1, XM_027372207.1 | 3.82E-02 | 1.21E-01 | 7.23E-01 |
| 384 | LOC113809021 | 98.95 | 33.65 | 46.84 | XM_027360508.1, XM_027360507.1 | 4.39E-06 | 3.97E-08 | 5.62E-01 |
| 385 | LOC113804153 | 8.59 | 6.01 | 6.26 | XM_027354991.1 | 5.97E-02 | 2.23E-02 | 7.94E-01 |
| 386 | LOC113824808 | 0.71 | 0.48 | 0.53 | XM_027377600.1 | 1.45E-01 | 1.96E-02 | 9.79E-01 |
| 387 | LOC113828122 | 0.67 | 0.49 | 1.19 | XM_027381061.1, BGI_novel_T008579 | 1.06E-01 | 4.09E-01 | 2.88E-02 |
| 388 | LOC113808628 | 0.53 | 0.12 | 1.81 | XM_027360091.1 | 4.85E-02 | 2.06E-01 | 5.61E-04 |
| 389 | LOC113818750 | 4.11 | 3.27 | 6.43 | XM_027370941.1 | 1.69E-01 | 4.51E-01 | 3.94E-02 |
| 390 | LOC113822182 | 4.91 | 2.54 | 3.32 | BGI_novel_T006795, XM_027374720.1 | 1.66E-02 | 1.83E-02 | 6.83E-01 |
| 391 | LOC113800422 | 0.03 | 0.00 | 0.13 | BGI_novel_T002876, XM_027351188.1, BGI_novel_T002877 | 6.36E-01 | 1.74E-01 | 3.89E-02 |
| 392 | LOC113828649 | 13.85 | 11.12 | 12.35 | XM_027381655.1 | 1.85E-01 | 3.02E-02 | 7.35E-01 |
| 393 | LOC113812628 | 12.26 | 3.72 | 9.52 | BGI_novel_T004149, XM_027364544.1 | 2.09E-04 | 1.78E-01 | 1.59E-01 |
| 394 | LOC113828507 | 6.68 | 4.38 | 7.06 | XM_027381490.1 | 4.68E-03 | 1.78E-01 | 1.55E-01 |
| 395 | LOC113812904 | 0.16 | 0.08 | 0.37 | XM_027364826.1 | 3.58E-01 | 4.58E-01 | 1.31E-02 |
| 396 | LOC113811738 | 0.73 | 0.65 | 1.37 | XM_027363538.1, BGI_novel_T000357 | 3.74E-01 | 3.01E-01 | 9.92E-03 |
| 397 | LOC113814314 | 2.03 | 1.02 | 1.77 | XM_027366364.1 | 5.48E-03 | 8.53E-02 | 1.88E-01 |
| 398 | LOC113804095 | 20.29 | 14.19 | 25.46 | XM_027354925.1, BGI_novel_T001538, BGI_novel_T001539 | 6.47E-03 | 5.39E-01 | 4.96E-02 |
| 399 | LOC113803300 | 2.90 | 0.52 | 3.82 | BGI_novel_T000114, BGI_novel_T000113, XM_027354066.1, BGI_novel_T000112 | 2.58E-04 | 1.95E-01 | 2.67E-03 |
| 400 | LOC113803875 | 0.31 | 0.00 | 0.50 | XM_027354685.1, XM_027354684.1, XM_027354686.1 | 1.45E-02 | 9.64E-01 | 1.18E-01 |
| 401 | LOC113802071 | 20.27 | 19.73 | 21.18 | XM_027352531.1, XM_027352530.1, XM_027352533.1, XM_027352529.1, XM_027352532.1 | 3.57E-01 | 1.72E-02 | 5.60E-01 |
| 402 | LOC113825727 | 35.62 | 29.35 | 30.00 | XM_027378562.1, BGI_novel_T007884 | 4.64E-01 | 2.09E-02 | 4.62E-01 |
| 403 | LOC113828342 | 4.53 | 1.71 | 3.83 | BGI_novel_T008626, BGI_novel_T008627, XM_027381282.1, XM_027381280.1, XM_027381281.1, XM_027381283.1 | 1.91E-04 | 1.34E-01 | 2.90E-02 |
| 404 | LOC113805361 | 6.06 | 4.46 | 9.85 | XM_027356349.1 | 1.24E-01 | 3.49E-01 | 1.66E-03 |
| 405 | LOC113824457 | 0.07 | 0.01 | 0.11 | XM_027377198.1 | 4.22E-02 | 9.38E-01 | 4.51E-02 |
| 406 | LOC113830375 | 0.13 | 0.12 | 0.46 | XM_027383588.1 | 5.66E-01 | 4.11E-02 | 3.07E-02 |
| 407 | LOC113822861 | 31.31 | 19.07 | 22.42 | XM_027375393.1, BGI_novel_T006959 | 1.50E-01 | 2.13E-03 | 8.66E-01 |
| 408 | LOC113822862 | 47.41 | 28.89 | 29.25 | BGI_novel_T006961, XM_027375394.1, BGI_novel_T006960 | 3.59E-03 | 9.37E-04 | 7.98E-01 |
| 409 | LOC113830588 | 53.61 | 31.91 | 35.74 | BGI_novel_T009253, XM_027383790.1, XM_027383792.1, BGI_novel_T009251, BGI_novel_T009252 | 2.27E-02 | 5.98E-04 | 6.75E-01 |
| 410 | LOC113801252 | 0.28 | 0.13 | 0.52 | XM_027352072.1 | 6.28E-02 | 6.67E-01 | 4.20E-02 |
| 411 | LOC113820073 | 0.78 | 0.41 | 1.35 | XM_027372328.1, XM_027372330.1, XM_027372329.1, BGI_novel_T006212, BGI_novel_T006213, XM_027372332.1, XM_027372331.1 | 9.52E-02 | 4.92E-01 | 2.33E-02 |
| 412 | LOC113817270 | 8.28 | 5.80 | 7.92 | XM_027369296.1 | 3.52E-02 | 2.02E-02 | 6.05E-01 |
| 413 | LOC113819965 | 0.77 | 0.19 | 1.41 | BGI_novel_T006181, XM_027372209.1 | 3.66E-02 | 5.55E-01 | 2.19E-02 |
| 414 | LOC113822269 | 1.23 | 0.51 | 0.94 | BGI_novel_T006828, XM_027374813.1, XM_027374814.1, XM_027374815.1 | 6.16E-04 | 2.25E-02 | 1.47E-01 |
| 415 | LOC113808158 | 0.62 | 0.12 | 0.88 | XM_027359489.1 | 1.48E-02 | 9.07E-01 | 7.28E-03 |
| 416 | LOC113814917 | 0.79 | 0.40 | 0.93 | XM_027366989.1, XM_027366990.1, XM_027366991.1 | 4.84E-02 | 8.54E-01 | 7.34E-02 |
| 417 | LOC113803904 | 3.52 | 2.13 | 3.31 | XM_027354714.1 | 1.25E-02 | 1.06E-01 | 2.84E-01 |
| 418 | LOC113829801 | 41.62 | 21.75 | 74.84 | XM_027382974.1, XM_027382975.1 | 1.13E-02 | 4.44E-01 | 1.44E-02 |
| 419 | LOC113808017 | 5.07 | 4.07 | 6.21 | XM_027359343.1, XM_027359344.1, BGI_novel_T002711, BGI_novel_T002710, XM_027359342.1, BGI_novel_T002709, BGI_novel_T002712 | 5.98E-03 | 6.74E-01 | 1.14E-01 |
| 420 | LOC113827743 | 1.93 | 1.15 | 1.29 | BGI_novel_T008476, XM_027380641.1, XM_027380639.1, BGI_novel_T008478, BGI_novel_T008475, XM_027380643.1, XM_027380640.1, XM_027380645.1, BGI_novel_T008477, XM_027380644.1, XM_027380642.1, XM_027380638.1 | 4.35E-02 | 1.32E-01 | 8.58E-01 |
| 421 | LOC113830510 | 13.77 | 6.45 | 13.43 | XM_027383722.1 | 1.68E-02 | 1.29E-01 | 9.06E-02 |
| 422 | LOC113816389 | 57.54 | 33.70 | 52.71 | BGI_novel_T000480, XM_027368462.1, XM_027368477.1, XM_027368485.1, BGI_novel_T000481, XM_027368470.1 | 5.79E-04 | 4.96E-02 | 1.48E-01 |
| 423 | LOC113821188 | 6.02 | 1.97 | 3.30 | XM_027373659.1 | 1.92E-03 | 1.53E-02 | 6.66E-01 |
| 424 | LOC113825593 | 0.69 | 0.68 | 1.40 | XM_027378426.1 | 6.43E-01 | 1.77E-01 | 3.08E-02 |
| 425 | LOC113828297 | 1.48 | 0.76 | 4.04 | XM_027381232.1, BGI_novel_T008622 | 2.36E-01 | 2.51E-01 | 5.66E-03 |
| 426 | LOC113826673 | 0.56 | 0.18 | 0.65 | XM_027379564.1 | 3.02E-02 | 6.72E-01 | 9.34E-02 |
| 427 | LOC113826860 | 59.85 | 48.36 | 94.96 | XM_027379756.1 | 1.15E-01 | 4.56E-01 | 4.74E-04 |
| 428 | BGI_novel_G000090 | 2.40 | 0.23 | 0.85 | BGI_novel_T000528 | 2.95E-04 | 4.28E-02 | 2.75E-01 |
| 429 | LOC113807690 | 125.75 | 59.79 | 95.50 | BGI_novel_T002571, XM_027359007.1 | 5.43E-03 | 2.60E-01 | 3.73E-01 |
| 430 | LOC113807693 | 82.91 | 21.62 | 26.25 | BGI_novel_T002574, XM_027359012.1 | 1.62E-02 | 3.70E-05 | 8.30E-01 |
| 431 | LOC113824768 | 7.99 | 5.42 | 6.45 | BGI_novel_T007646, XM_027377555.1, XM_027377556.1 | 4.48E-02 | 1.86E-01 | 7.26E-01 |
| 432 | LOC113816543 | 1.72 | 0.46 | 1.77 | BGI_novel_T005276, XM_027368576.1 | 2.24E-02 | 7.01E-01 | 5.92E-03 |
| 433 | LOC113812971 | 19.57 | 10.08 | 13.01 | XM_027364909.1 | 2.02E-03 | 5.48E-03 | 6.90E-01 |
| 434 | LOC113823155 | 506.81 | 216.56 | 295.87 | XM_027375771.1 | 4.76E-02 | 1.23E-01 | 8.27E-01 |
| 435 | BGI_novel_G001487 | 5.34 | 1.76 | 3.33 | BGI_novel_T009109 | 6.64E-11 | 9.25E-05 | 7.10E-03 |
| 436 | LOC113811244 | 171.04 | 102.41 | 123.85 | XM_027362936.1 | 1.07E-03 | 2.25E-03 | 8.48E-01 |
| 437 | LOC113826551 | 4.24 | 2.37 | 5.42 | XM_027379452.1 | 9.64E-02 | 7.18E-01 | 2.08E-02 |
| 438 | LOC113808497 | 2.20 | 0.96 | 1.14 | BGI_novel_T002815, XM_027359914.1 | 2.43E-02 | 1.32E-02 | 9.23E-01 |
| 439 | LOC113810963 | 0.27 | 0.17 | 2.22 | BGI_novel_T003681, XM_027362632.1, BGI_novel_T003683, XM_027362631.1, XM_027362629.1, XM_027362630.1, BGI_novel_T003682 | 4.30E-01 | 4.90E-02 | 1.88E-02 |
| 440 | LOC113822379 | 4.90 | 2.41 | 5.92 | XM_027374913.1 | 2.27E-02 | 5.48E-01 | 2.94E-01 |
| 441 | LOC113821178 | 0.71 | 0.12 | 3.28 | XM_027373648.1, BGI_novel_T006527 | 4.10E-02 | 1.77E-03 | 6.44E-06 |
| 442 | LOC113822427 | 3.09 | 1.11 | 3.70 | BGI_novel_T006877, XM_027374965.1, BGI_novel_T006875, BGI_novel_T006874, BGI_novel_T006876 | 9.71E-05 | 8.93E-01 | 1.22E-02 |
| 443 | LOC113829827 | 0.45 | 0.45 | 1.00 | XM_027382998.1 | 7.28E-01 | 8.10E-02 | 1.53E-02 |
| 444 | LOC113812877 | 0.50 | 0.20 | 0.45 | XM_027364795.1 | 9.07E-03 | 2.14E-01 | 1.88E-01 |
| 445 | LOC113827196 | 1.60 | 0.78 | 1.55 | BGI_novel_T008301, XM_027380093.1 | 1.82E-03 | 7.01E-01 | 9.05E-02 |
| 446 | LOC113818188 | 10.69 | 6.89 | 9.88 | XM_027370359.1, XM_027370360.1, XM_027370361.1, XM_027370358.1 | 6.10E-03 | 3.90E-03 | 4.71E-01 |
| 447 | LOC113825594 | 79.59 | 33.60 | 59.17 | XM_027378431.1, BGI_novel_T007859, BGI_novel_T007857, XM_027378433.1, BGI_novel_T007858, XM_027378427.1, XM_027378430.1, XM_027378428.1, BGI_novel_T007856, XM_027378429.1, XM_027378432.1 | 1.72E-08 | 1.42E-02 | 8.27E-02 |
| 448 | LOC113819905 | 0.32 | 0.21 | 0.63 | XM_027372142.1 | 3.15E-01 | 3.42E-01 | 3.99E-02 |
| 449 | LOC113813239 | 546.52 | 54.00 | 253.82 | XM_027365200.1 | 5.81E-05 | 6.11E-02 | 1.68E-02 |
| 450 | LOC113829207 | 0.29 | 0.05 | 0.34 | BGI_novel_T008975, XM_027382321.1 | 3.80E-02 | 8.90E-01 | 3.31E-02 |
| 451 | LOC113830554 | 18.60 | 11.44 | 16.60 | XM_027383757.1, XM_027383760.1, XM_027383759.1 | 9.46E-03 | 2.71E-02 | 4.23E-01 |
| 452 | LOC113818094 | 0.24 | 0.04 | 0.15 | XM_027370230.1 | 2.17E-02 | 1.21E-01 | 1.90E-01 |
| 453 | LOC113813240 | 18.79 | 0.77 | 4.39 | XM_027365203.1, BGI_novel_T004283, XM_027365201.1, BGI_novel_T004282 | 1.77E-05 | 2.85E-02 | 5.97E-02 |
| 454 | LOC113827352 | 1.87 | 1.42 | 2.93 | XM_027380233.1 | 5.19E-01 | 6.68E-01 | 2.42E-02 |
| 455 | LOC113820228 | 0.84 | 0.22 | 1.28 | XM_027372577.1 | 2.31E-03 | 5.90E-01 | 1.96E-03 |
| 456 | LOC113820506 | 0.80 | 0.35 | 1.22 | XM_027372858.1 | 1.54E-02 | 6.87E-01 | 8.87E-03 |
| 457 | LOC113829599 | 0.04 | 0.00 | 0.13 | XM_027382796.1, XM_027382797.1 | 6.19E-02 | 3.98E-01 | 2.10E-02 |
| 458 | LOC113830044 | 15.64 | 10.85 | 15.31 | XM_027383235.1, XM_027383234.1 | 3.47E-02 | 2.22E-01 | 4.76E-01 |
| 459 | BGI_novel_G001364 | 0.38 | 0.05 | 0.11 | BGI_novel_T008175 | 1.54E-02 | 7.34E-02 | 7.01E-01 |
| 460 | LOC113807800 | 21.52 | 15.23 | 23.02 | BGI_novel_T002625, XM_027359089.1, BGI_novel_T002624 | 1.03E-02 | 1.07E-01 | 4.90E-01 |
| 461 | LOC113807350 | 1036.66 | 659.37 | 1798.56 | XM_027358583.1 | 3.66E-02 | 3.95E-01 | 2.58E-03 |
| 462 | LOC113805690 | 0.10 | 0.04 | 1.03 | XM_027356745.1 | 6.87E-01 | 4.32E-02 | 1.76E-02 |
| 463 | LOC113810982 | 0.03 | 0.00 | 0.08 | XM_027362654.1, XM_027362655.1 | 7.25E-02 | 4.58E-01 | 1.58E-02 |
| 464 | LOC113806267 | 2.49 | 1.90 | 3.25 | XM_027357382.1, XM_027357383.1 | 2.97E-02 | 9.50E-01 | 1.08E-01 |
| 465 | LOC113809514 | 0.75 | 0.30 | 0.65 | XM_027361142.1, XM_027361141.1 | 1.94E-04 | 6.24E-02 | 6.35E-02 |
| 466 | LOC113828640 | 3.63 | 2.65 | 6.67 | XM_027381646.1, BGI_novel_T008718 | 2.92E-02 | 2.57E-02 | 6.50E-06 |
| 467 | LOC113830152 | 4.33 | 0.39 | 0.92 | XM_027383357.1 | 3.89E-02 | 1.21E-01 | 4.04E-01 |
| 468 | LOC113812644 | 0.19 | 0.05 | 0.50 | XM_027364562.1 | 3.21E-02 | 5.41E-01 | 4.63E-02 |
| 469 | LOC113807037 | 0.44 | 0.23 | 0.67 | XM_027358197.1 | 2.73E-02 | 9.74E-01 | 1.01E-01 |
| 470 | LOC113813494 | 2.18 | 1.87 | 2.02 | XM_027365488.1 | 1.43E-01 | 3.07E-02 | 5.57E-01 |
| 471 | LOC113826481 | 1.06 | 0.58 | 1.74 | XM_027379371.1, XM_027379372.1 | 6.69E-02 | 4.91E-01 | 1.81E-02 |
| 472 | LOC113806231 | 2.26 | 1.57 | 2.52 | XM_027357359.1, BGI_novel_T000194 | 1.41E-04 | 1.11E-01 | 8.77E-02 |
| 473 | LOC113805233 | 0.21 | 0.01 | 0.25 | XM_027356197.1, BGI_novel_T001722 | 4.42E-05 | 9.62E-01 | 2.93E-05 |
| 474 | LOC113812522 | 41.17 | 18.15 | 47.85 | XM_027364428.1, BGI_novel_T004118, BGI_novel_T004119 | 3.70E-03 | 8.11E-01 | 3.64E-02 |
| 475 | LOC113825420 | 8.77 | 5.65 | 8.64 | XM_027378257.1, XM_027378262.1, XM_027378251.1 | 4.79E-02 | 7.89E-02 | 4.09E-01 |
| 476 | LOC113810183 | 3.52 | 2.51 | 2.95 | BGI_novel_T003389, BGI_novel_T003392, BGI_novel_T003390, XM_027361886.1, BGI_novel_T003391 | 3.92E-02 | 6.93E-02 | 8.12E-01 |
| 477 | LOC113809510 | 3.02 | 2.56 | 5.54 | XM_027361136.1, XM_027361137.1, BGI_novel_T003201, XM_027361131.1, XM_027361135.1, XM_027361133.1, BGI_novel_T003203, XM_027361134.1, BGI_novel_T003202, BGI_novel_T003204, XM_027361132.1 | 9.20E-02 | 2.02E-01 | 2.26E-02 |
| 478 | LOC113817500 | 2.46 | 0.98 | 2.40 | XM_027369572.1 | 4.87E-02 | 4.13E-01 | 3.99E-01 |
| 479 | LOC113809456 | 0.77 | 0.26 | 0.72 | XM_027361066.1 | 1.84E-02 | 3.51E-01 | 6.34E-02 |
| 480 | LOC113822019 | 2.96 | 1.28 | 2.57 | XM_027374530.1 | 1.47E-08 | 2.73E-02 | 1.94E-02 |
| 481 | BGI_novel_G000663 | 0.45 | 0.04 | 0.32 | BGI_novel_T003996 | 1.02E-02 | 5.02E-01 | 2.05E-01 |
| 482 | LOC113817630 | 0.58 | 0.41 | 0.57 | XM_027369684.1, XM_027369683.1 | 1.48E-02 | 2.24E-02 | 4.35E-01 |
| 483 | LOC113821777 | 1.71 | 1.11 | 2.41 | XM_027374303.1 | 2.59E-02 | 8.48E-01 | 1.35E-02 |
| 484 | LOC113825974 | 1.44 | 0.76 | 1.67 | XM_027378850.1, XM_027378856.1, BGI_novel_T000818 | 2.10E-02 | 5.88E-01 | 6.49E-02 |
| 485 | LOC113805768 | 0.06 | 0.02 | 0.09 | XM_027356825.1 | 3.41E-02 | 9.14E-01 | 6.18E-02 |
| 486 | LOC113809665 | 0.21 | 0.08 | 0.36 | XM_027361323.1, XM_027361322.1 | 4.17E-02 | 5.76E-01 | 4.09E-03 |
| 487 | LOC113814991 | 19.83 | 15.18 | 22.59 | BGI_novel_T004819, BGI_novel_T004817, XM_027367070.1, BGI_novel_T004818, XM_027367068.1, XM_027367069.1 | 4.86E-03 | 1.57E-01 | 4.12E-02 |
| 488 | LOC113815002 | 0.86 | 0.56 | 2.51 | XM_027367087.1 | 3.34E-01 | 4.47E-02 | 4.24E-03 |
| 489 | LOC113803985 | 3.63 | 1.68 | 2.29 | XM_027354795.1 | 3.37E-04 | 8.69E-04 | 6.02E-01 |
| 490 | LOC113805917 | 0.43 | 0.29 | 0.36 | XM_027356988.1 | 3.11E-02 | 3.80E-02 | 8.90E-01 |
| 491 | LOC113808278 | 0.65 | 0.11 | 0.91 | XM_027359628.1 | 5.21E-04 | 9.18E-01 | 4.52E-04 |
| 492 | LOC113819086 | 0.34 | 0.10 | 0.16 | XM_027371328.1 | 7.84E-03 | 7.25E-02 | 6.72E-01 |
| 493 | LOC113823896 | 0.16 | 0.00 | 0.21 | XM_027376620.1 | 2.46E-02 | 7.81E-01 | 1.48E-01 |
| 494 | LOC113808656 | 1.33 | 0.90 | 1.19 | XM_027360124.1, XM_027360123.1 | 4.39E-02 | 4.02E-02 | 8.24E-01 |
| 495 | LOC113808189 | 10.73 | 7.54 | 10.26 | BGI_novel_T002749, XM_027359526.1 | 1.13E-03 | 2.33E-02 | 4.02E-01 |
| 496 | LOC113809499 | 4.53 | 3.16 | 4.48 | XM_027361114.1, XM_027361113.1 | 2.15E-01 | 3.09E-02 | 6.57E-01 |
| 497 | LOC113818187 | 0.74 | 0.45 | 0.68 | XM_027370356.1, XM_027370355.1, XM_027370357.1 | 1.48E-02 | 6.34E-02 | 3.36E-01 |
| 498 | LOC113808280 | 1.50 | 1.04 | 1.60 | BGI_novel_T002763, BGI_novel_T002764, XM_027359630.1 | 4.51E-02 | 2.28E-01 | 2.39E-01 |
| 499 | LOC113829222 | 1.06 | 0.03 | 1.76 | XM_027382337.1 | 2.26E-01 | 9.28E-01 | 4.06E-03 |
| 500 | LOC113825738 | 726.76 | 651.11 | 743.22 | BGI_novel_T007899, XM_027378572.1 | 9.46E-03 | 1.16E-01 | 9.39E-01 |
| 501 | LOC113817075 | 61.54 | 36.64 | 55.08 | XM_027369072.1, BGI_novel_T005364 | 6.57E-03 | 1.37E-01 | 5.04E-01 |
| 502 | LOC113809073 | 1.99 | 1.16 | 6.21 | XM_027360572.1, XM_027360566.1, XM_027360579.1, XM_027360586.1 | 1.55E-01 | 2.80E-01 | 2.85E-02 |
| 503 | LOC113806435 | 0.20 | 0.00 | 0.38 | XM_027357557.1 | 4.51E-03 | 9.74E-01 | 3.10E-02 |
| 504 | LOC113819917 | 10.92 | 8.76 | 11.51 | XM_027372150.1 | 1.14E-02 | 3.69E-01 | 5.58E-01 |
| 505 | LOC113809804 | 2.89 | 2.34 | 3.87 | XM_027361484.1, BGI_novel_T003292, XM_027361482.1, BGI_novel_T003294, XM_027361483.1, XM_027361481.1, BGI_novel_T003293 | 2.55E-02 | 8.94E-01 | 2.84E-02 |
| 506 | LOC113821404 | 4.71 | 4.15 | 4.63 | XM_027373888.1 | 3.36E-01 | 2.74E-02 | 7.48E-01 |
| 507 | LOC113808919 | 0.52 | 0.13 | 0.84 | XM_027360429.1, BGI_novel_T000269, BGI_novel_T000268, XM_027360427.1, XM_027360428.1 | 3.85E-02 | 3.86E-01 | 1.92E-03 |
| 508 | LOC113827261 | 1.35 | 0.67 | 1.43 | XM_027380158.1, XM_027380159.1 | 4.27E-02 | 4.03E-01 | 2.70E-01 |
| 509 | LOC113817924 | 462.31 | 293.77 | 325.70 | XM_027370046.1 | 3.42E-02 | 4.86E-03 | 7.14E-01 |
| 510 | LOC113805590 | 42.42 | 13.04 | 22.52 | XM_027356614.1 | 2.45E-03 | 3.29E-03 | 3.98E-01 |
| 511 | LOC113804378 | 0.08 | 0.03 | 0.23 | XM_027355234.1 | 3.16E-01 | 4.85E-01 | 1.87E-02 |
| 512 | LOC113816703 | 0.12 | 0.03 | 0.28 | XM_027368760.1 | 2.81E-01 | 6.27E-01 | 4.00E-02 |
| 513 | LOC113823785 | 440.52 | 2.54 | 209.67 | XM_027376476.1 | 9.32E-21 | 5.36E-01 | 2.75E-04 |
| 514 | LOC113817090 | 711.10 | 192.43 | 419.48 | BGI_novel_T005370, XM_027369090.1 | 2.29E-03 | 1.74E-01 | 1.88E-01 |
| 515 | LOC113800094 | 9.06 | 6.98 | 10.93 | XM_027350818.1, XM_027350817.1 | 4.36E-02 | 4.61E-01 | 1.67E-01 |
| 516 | LOC113817742 | 1.45 | 0.25 | 2.90 | BGI_novel_T005567, BGI_novel_T005570, BGI_novel_T005568, XM_027369828.1, BGI_novel_T005569 | 1.19E-02 | 8.40E-01 | 4.43E-02 |
| 517 | LOC113807569 | 3.10 | 1.78 | 2.70 | XM_027358858.1 | 3.81E-02 | 9.57E-02 | 4.39E-01 |
| 518 | LOC113817761 | 1.22 | 0.17 | 1.74 | XM_027369857.1 | 2.71E-02 | 8.88E-01 | 7.88E-02 |
| 519 | LOC113819409 | 7.85 | 5.60 | 9.18 | BGI_novel_T006025, BGI_novel_T006024, XM_027371650.1 | 4.43E-02 | 3.63E-01 | 1.57E-01 |
| 520 | LOC113829432 | 1.11 | 0.56 | 1.37 | XM_027382607.1 | 3.43E-02 | 8.77E-01 | 8.23E-02 |
| 521 | LOC113820462 | 1.50 | 0.63 | 1.41 | XM_027372780.1, BGI_novel_T006316, BGI_novel_T006315, BGI_novel_T006317 | 2.54E-02 | 4.41E-01 | 1.26E-01 |
| 522 | LOC113823354 | 27.91 | 24.93 | 33.50 | XM_027375982.1, XM_027375983.1 | 4.02E-02 | 1.54E-01 | 3.24E-01 |
| 523 | LOC113822116 | 1.34 | 1.33 | 2.41 | XM_027374643.1 | 4.05E-01 | 2.21E-02 | 6.47E-03 |
| 524 | LOC113804210 | 28.80 | 10.59 | 18.65 | XM_027355042.1 | 3.96E-06 | 4.04E-02 | 3.62E-01 |
| 525 | BGI_novel_G000560 | 6.59 | 2.00 | 4.22 | BGI_novel_T003365 | 1.48E-05 | 4.28E-02 | 2.92E-01 |
| 526 | LOC113820876 | 1.55 | 0.87 | 1.45 | XM_027373262.1 | 2.55E-03 | 4.49E-02 | 3.07E-01 |
| 527 | LOC113809573 | 10.70 | 5.95 | 7.47 | XM_027361202.1, BGI_novel_T003236 | 1.96E-02 | 2.36E-02 | 7.96E-01 |
| 528 | LOC113822945 | 35.57 | 23.32 | 26.12 | XM_027375494.1 | 2.41E-02 | 2.38E-02 | 7.72E-01 |
| 529 | BGI_novel_G001284 | 15.14 | 10.68 | 15.38 | BGI_novel_T007752 | 8.60E-03 | 2.98E-01 | 3.69E-01 |
| 530 | LOC113816418 | 16.84 | 7.38 | 8.35 | BGI_novel_T000483, BGI_novel_T000482, XM_027368490.1 | 4.26E-02 | 1.31E-02 | 3.85E-01 |
| 531 | LOC113816547 | 8.31 | 3.85 | 3.86 | XM_027368585.1, BGI_novel_T000492, BGI_novel_T000493 | 3.42E-02 | 5.49E-03 | 3.99E-01 |
| 532 | LOC113829643 | 174.00 | 54.41 | 75.43 | XM_027382850.1, XM_027382852.1 | 2.72E-03 | 2.56E-03 | 6.36E-01 |
| 533 | LOC113802990 | 124.19 | 24.15 | 57.73 | XM_027353684.1 | 1.18E-09 | 6.06E-12 | 2.49E-02 |
| 534 | LOC113808051 | 0.17 | 0.12 | 0.40 | XM_027359374.1, XM_027359375.1 | 3.90E-01 | 2.98E-01 | 4.41E-02 |
| 535 | LOC113828010 | 17.21 | 9.30 | 18.83 | XM_027380950.1, BGI_novel_T008553 | 5.45E-03 | 2.74E-01 | 8.70E-02 |
| 536 | LOC113827474 | 0.78 | 0.13 | 0.58 | XM_027380364.1 | 1.57E-02 | 3.85E-01 | 1.92E-01 |
| 537 | LOC113819879 | 2.54 | 1.84 | 2.99 | XM_027372103.1 | 1.87E-02 | 3.75E-01 | 1.25E-01 |
| 538 | LOC113813316 | 5.42 | 5.18 | 5.40 | XM_027365297.1, XM_027365295.1, BGI_novel_T004315 | 2.59E-01 | 2.63E-02 | 3.16E-01 |
| 539 | LOC113802030 | 8.94 | 6.19 | 11.03 | BGI_novel_T001016, XM_027352514.1, XM_027352505.1 | 1.63E-02 | 8.08E-01 | 8.32E-02 |
| 540 | LOC113825379 | 0.55 | 0.47 | 1.23 | XM_027378202.1, BGI_novel_T000797, BGI_novel_T000796 | 4.33E-01 | 3.44E-02 | 1.01E-02 |
| 541 | LOC113809822 | 0.12 | 0.05 | 0.30 | XM_027361500.1 | 6.34E-02 | 5.52E-01 | 2.14E-02 |
| 542 | LOC113809316 | 2.99 | 2.33 | 4.80 | XM_027360861.1 | 3.02E-02 | 1.64E-01 | 2.74E-03 |
| 543 | LOC113829616 | 0.46 | 0.22 | 0.81 | XM_027382815.1 | 9.68E-02 | 6.62E-01 | 3.00E-02 |
| 544 | LOC113817069 | 20.32 | 14.94 | 22.17 | XM_027369068.1, BGI_novel_T005357, BGI_novel_T005358, BGI_novel_T005359 | 4.88E-02 | 3.15E-02 | 3.38E-01 |
| 545 | LOC113804735 | 0.06 | 0.02 | 0.19 | XM_027355627.1 | 1.42E-01 | 4.58E-01 | 3.31E-02 |
| 546 | LOC113811463 | 9.90 | 7.93 | 11.75 | XM_027363218.1, BGI_novel_T003776, XM_027363232.1, XM_027363228.1, XM_027363223.1, BGI_novel_T003778, XM_027363215.1, XM_027363209.1, XM_027363208.1, XM_027363217.1, XM_027363214.1, BGI_novel_T003779, BGI_novel_T003777, XM_027363220.1, XM_027363221.1, XM_027363226.1, XM_027363210.1, XM_027363216.1, XM_027363230.1, XM_027363229.1, XM_027363211.1, XM_027363224.1, XM_027363212.1, XM_027363225.1, XM_027363222.1, XM_027363227.1, XM_027363219.1, XM_027363213.1 | 2.68E-02 | 3.32E-01 | 2.02E-01 |
| 547 | LOC113802305 | 5.52 | 3.48 | 9.27 | BGI_novel_T005004, BGI_novel_T005001, BGI_novel_T004998, BGI_novel_T005002, BGI_novel_T004999, BGI_novel_T005003, BGI_novel_T005000, XM_027352856.1 | 8.36E-02 | 2.63E-01 | 2.56E-02 |
| 548 | LOC113804358 | 0.27 | 0.18 | 0.66 | BGI_novel_T001426, XM_027355214.1 | 2.23E-01 | 1.48E-01 | 1.83E-02 |
| 549 | LOC113827591 | 0.50 | 0.33 | 0.81 | XM_027380488.1, XM_027380487.1 | 1.46E-01 | 7.17E-01 | 4.28E-02 |
| 550 | LOC113820528 | 0.35 | 0.18 | 0.50 | XM_027372882.1, XM_027372881.1 | 4.61E-02 | 9.77E-01 | 7.39E-02 |
| 551 | LOC113822761 | 0.81 | 0.29 | 0.64 | XM_027375300.1 | 1.53E-03 | 1.89E-01 | 3.72E-01 |
| 552 | LOC113820810 | 0.85 | 0.69 | 0.92 | XM_027373174.1 | 3.21E-02 | 1.51E-01 | 5.88E-01 |
| 553 | LOC113809581 | 7.65 | 6.98 | 7.82 | BGI_novel_T003227, XM_027361208.1 | 5.44E-02 | 4.45E-02 | 6.83E-01 |
| 554 | LOC113820436 | 1.11 | 0.68 | 0.86 | XM_027372768.1 | 9.77E-03 | 3.26E-02 | 8.88E-01 |
| 555 | LOC113822911 | 7.37 | 4.65 | 9.73 | BGI_novel_T006991, XM_027375451.1, XM_027375450.1 | 3.47E-02 | 8.37E-01 | 4.34E-02 |
| 556 | LOC113815867 | 68.54 | 38.19 | 57.07 | XM_027367881.1 | 7.26E-02 | 1.34E-03 | 6.56E-01 |
| 557 | LOC113808685 | 1.70 | 1.19 | 1.35 | XM_027360158.1, BGI_novel_T002893 | 2.24E-01 | 3.78E-02 | 9.86E-01 |
| 558 | LOC113813246 | 1.61 | 0.88 | 1.03 | XM_027365214.1 | 2.25E-03 | 3.49E-03 | 9.93E-01 |
| 559 | LOC113802179 | 13.94 | 7.49 | 37.46 | XM_027352707.1 | 2.85E-02 | 3.64E-01 | 1.35E-02 |
| 560 | LOC113804867 | 1.31 | 0.93 | 1.29 | BGI_novel_T001579, XM_027355776.1, BGI_novel_T001578 | 4.00E-02 | 8.52E-02 | 5.49E-01 |
| 561 | LOC113820749 | 3.99 | 3.16 | 3.61 | XM_027373104.1, BGI_novel_T006387, BGI_novel_T006388 | 3.78E-01 | 1.87E-02 | 6.48E-01 |
| 562 | LOC113800274 | 80.35 | 53.06 | 62.13 | XM_027351017.1 | 4.39E-03 | 1.60E-02 | 8.83E-01 |
| 563 | LOC113806361 | 23.41 | 1.55 | 9.28 | BGI_novel_T002088, XM_027357486.1, BGI_novel_T002098, BGI_novel_T002097 | 3.52E-03 | 6.13E-01 | 2.25E-01 |
| 564 | LOC113815482 | 0.03 | 0.02 | 0.17 | XM_027367545.1 | 7.58E-01 | 9.65E-02 | 2.38E-02 |
| 565 | LOC113815953 | 0.31 | 0.13 | 0.39 | XM_027367965.1, XM_027367964.1 | 1.36E-02 | 8.44E-01 | 4.50E-02 |
| 566 | BGI_novel_G000497 | 0.11 | 0.00 | 0.03 | BGI_novel_T003041 | 4.38E-03 | 7.31E-02 | 3.43E-01 |
| 567 | LOC113817085 | 8.24 | 2.49 | 4.79 | XM_027369082.1, BGI_novel_T005368 | 4.71E-04 | 1.30E-04 | 3.66E-01 |
| 568 | LOC113826325 | 0.87 | 0.54 | 0.66 | XM_027379195.1 | 1.95E-01 | 4.40E-02 | 9.57E-01 |
| 569 | LOC113819444 | 0.83 | 0.00 | 2.63 | BGI_novel_T006034, XM_027371678.1 | 1.75E-01 | 7.37E-01 | 2.81E-03 |
| 570 | LOC113804789 | 0.01 | 0.00 | 0.11 | XM_027355688.1, XM_027355687.1 | 5.61E-01 | 5.07E-02 | 4.30E-03 |
| 571 | LOC113808626 | 19.61 | 9.20 | 20.92 | XM_027360088.1 | 9.41E-03 | 3.30E-01 | 3.88E-02 |
| 572 | LOC113817682 | 15.77 | 8.39 | 13.14 | XM_027369759.1 | 2.08E-02 | 2.26E-01 | 2.64E-01 |
| 573 | LOC113814483 | 22.11 | 6.38 | 9.92 | XM_027366512.1, BGI_novel_T004690 | 1.04E-02 | 1.54E-02 | 5.91E-01 |
| 574 | LOC113829016 | 48.83 | 38.44 | 47.17 | BGI_novel_T008896, XM_027382094.1 | 2.19E-01 | 4.69E-02 | 9.60E-01 |
| 575 | LOC113820580 | 0.28 | 0.02 | 0.05 | BGI_novel_T006346, XM_027372915.1 | 9.76E-03 | 2.73E-02 | 7.36E-01 |
| 576 | LOC113803125 | 8.13 | 6.69 | 6.86 | XM_027353844.1, XM_027353843.1, XM_027353845.1 | 3.09E-01 | 3.22E-02 | 4.00E-01 |
| 577 | LOC113825330 | 3.80 | 2.30 | 3.61 | XM_027378149.1 | 3.57E-06 | 1.25E-01 | 5.53E-01 |
| 578 | LOC113802161 | 9.58 | 7.45 | 8.88 | XM_027352683.1 | 2.41E-01 | 4.19E-02 | 9.24E-01 |
| 579 | LOC113800464 | 71.39 | 36.89 | 54.64 | XM_027351245.1 | 6.51E-03 | 1.73E-03 | 4.13E-01 |
| 580 | LOC113808717 | 10.59 | 4.94 | 11.51 | BGI_novel_T002902, XM_027360190.1, BGI_novel_T002901 | 2.19E-02 | 4.71E-01 | 1.04E-01 |
| 581 | BGI_novel_G001621 | 1.64 | 0.54 | 1.90 | BGI_novel_T009719 | 3.25E-02 | 5.43E-01 | 5.03E-02 |
| 582 | LOC113809427 | 8.42 | 3.64 | 7.36 | XM_027361022.1 | 6.13E-03 | 1.66E-01 | 2.94E-01 |
| 583 | LOC113807387 | 0.66 | 0.44 | 1.34 | XM_027358628.1, XM_027358630.1, XM_027358629.1, XM_027358631.1, XM_027358625.1, XM_027358627.1, XM_027358632.1 | 1.17E-01 | 2.03E-01 | 8.87E-03 |
| 584 | LOC113808874 | 2.88 | 2.24 | 2.91 | XM_027360376.1, BGI_novel_T002968 | 2.14E-02 | 1.31E-01 | 5.73E-01 |
| 585 | LOC113805887 | 42.04 | 18.85 | 25.71 | XM_027356947.1, XM_027356948.1, XM_027356949.1, BGI_novel_T001944 | 5.27E-03 | 2.12E-02 | 5.56E-01 |
| 586 | LOC113824838 | 5.08 | 1.39 | 2.43 | BGI_novel_T007662, BGI_novel_T007661, XM_027377624.1 | 5.61E-04 | 6.24E-02 | 5.86E-01 |
| 587 | LOC113809781 | 0.85 | 0.49 | 1.00 | XM_027361454.1 | 3.36E-02 | 4.95E-01 | 1.17E-01 |
| 588 | LOC113810390 | 1.49 | 1.02 | 2.25 | BGI_novel_T003465, XM_027362074.1 | 5.14E-02 | 8.06E-01 | 4.56E-02 |
| 589 | LOC113805136 | 2.18 | 1.27 | 2.37 | BGI_novel_T001686, XM_027356081.1 | 2.75E-02 | 5.13E-01 | 2.54E-01 |
| 590 | LOC113803937 | 4.40 | 3.66 | 5.09 | BGI_novel_T000132, BGI_novel_T000134, BGI_novel_T000133, XM_027354751.1 | 4.76E-02 | 7.28E-02 | 3.55E-01 |
| 591 | BGI_novel_G000004 | 0.39 | 0.00 | 2.12 | BGI_novel_T000005 | 2.60E-02 | 3.03E-01 | 5.75E-02 |
| 592 | LOC113816925 | 5.57 | 4.37 | 5.24 | XM_027368917.1 | 2.41E-02 | 2.17E-02 | 9.15E-01 |
| 593 | LOC113826386 | 3.16 | 1.82 | 2.68 | XM_027379269.1, XM_027379270.1 | 1.58E-02 | 1.73E-01 | 5.63E-01 |
| 594 | LOC113803545 | 3.14 | 2.63 | 4.78 | XM_027354330.1 | 7.75E-02 | 3.64E-01 | 1.02E-02 |
| 595 | BGI_novel_G001087 | 0.88 | 0.28 | 0.34 | BGI_novel_T006525 | 9.62E-02 | 1.01E-02 | 8.05E-01 |
| 596 | LOC113819822 | 0.72 | 0.51 | 1.05 | XM_027372017.1 | 8.67E-02 | 6.24E-01 | 3.91E-02 |
| 597 | BGI_novel_G000279 | 0.09 | 0.02 | 0.49 | BGI_novel_T001702 | 3.36E-01 | 9.82E-03 | 7.24E-05 |
| 598 | LOC113809799 | 24.06 | 17.11 | 22.45 | XM_027361474.1, XM_027361473.1 | 2.41E-05 | 6.01E-02 | 5.84E-01 |
| 599 | LOC113818277 | 0.71 | 0.23 | 0.60 | XM_027370461.1, BGI_novel_T005685 | 4.29E-02 | 2.50E-01 | 5.46E-01 |
| 600 | LOC113829578 | 1.05 | 0.43 | 0.72 | XM_027382774.1 | 3.23E-04 | 1.87E-02 | 3.67E-01 |
| 601 | LOC113809430 | 4.95 | 2.97 | 4.97 | XM_027361027.1, XM_027361029.1, XM_027361028.1, XM_027361030.1 | 9.22E-06 | 1.23E-01 | 9.54E-02 |
| 602 | LOC113824007 | 1.89 | 1.39 | 2.54 | BGI_novel_T007389, XM_027376748.1 | 4.52E-02 | 8.85E-01 | 1.32E-01 |
| 603 | LOC113812910 | 1.86 | 0.72 | 1.06 | XM_027364833.1 | 3.90E-05 | 8.96E-02 | 5.16E-01 |
| 604 | LOC113817416 | 5.03 | 2.21 | 3.92 | XM_027369480.1 | 2.15E-03 | 6.67E-02 | 2.83E-01 |
| 605 | LOC113822673 | 0.45 | 0.19 | 0.22 | XM_027375212.1 | 5.11E-03 | 1.67E-03 | 7.71E-01 |
| 606 | LOC113828059 | 303.67 | 128.52 | 184.15 | XM_027380998.1 | 5.47E-04 | 1.23E-02 | 4.54E-01 |
| 607 | LOC113829233 | 0.55 | 0.09 | 0.26 | XM_027382349.1 | 2.78E-03 | 6.08E-02 | 2.72E-01 |
| 608 | LOC113824002 | 0.16 | 0.12 | 0.41 | XM_027376742.1, XM_027376744.1 | 4.27E-01 | 2.10E-01 | 3.69E-02 |
| 609 | LOC113814947 | 23.03 | 6.94 | 13.59 | BGI_novel_T004802, XM_027367018.1, XM_027367019.1 | 1.34E-04 | 5.83E-02 | 2.49E-01 |
| 610 | LOC113818220 | 95.64 | 58.81 | 71.83 | XM_027370399.1, XM_027370398.1 | 9.25E-04 | 2.08E-03 | 8.42E-01 |
| 611 | LOC113814526 | 176.03 | 145.68 | 148.00 | XM_027366568.1 | 2.93E-01 | 4.35E-02 | 6.28E-01 |
| 612 | LOC113827618 | 103.97 | 66.45 | 67.05 | XM_027380510.1, XM_027380509.1, XM_027380507.1 | 6.21E-03 | 1.42E-03 | 5.38E-01 |
| 613 | LOC113808979 | 3.63 | 2.69 | 4.44 | XM_027360463.1 | 2.31E-02 | 7.24E-01 | 1.59E-01 |
| 614 | LOC113817767 | 2.96 | 2.23 | 4.29 | BGI_novel_T005579, BGI_novel_T005577, XM_027369861.1, BGI_novel_T005578 | 6.45E-02 | 8.75E-01 | 4.97E-02 |
| 615 | LOC113819214 | 65.65 | 37.35 | 76.83 | XM_027371463.1, XM_027371462.1 | 8.48E-05 | 7.69E-01 | 4.05E-02 |
| 616 | LOC113818799 | 1.26 | 0.66 | 1.22 | XM_027370989.1 | 4.73E-02 | 8.62E-02 | 3.53E-01 |
| 617 | LOC113811684 | 27.88 | 13.96 | 19.94 | XM_027363473.1 | 1.27E-02 | 2.73E-02 | 6.05E-01 |
| 618 | LOC113804791 | 41.29 | 20.20 | 24.19 | XM_027355690.1 | 2.53E-02 | 2.69E-03 | 9.16E-01 |
| 619 | LOC113810833 | 0.11 | 0.01 | 0.56 | XM_027362512.1 | 3.24E-02 | 2.50E-01 | 3.80E-02 |
| 620 | LOC113824355 | 11.53 | 5.18 | 7.37 | XM_027377101.1 | 4.84E-02 | 1.55E-01 | 3.56E-01 |
| 621 | LOC113820256 | 940.13 | 445.62 | 565.13 | BGI_novel_T006264, XM_027372572.1, BGI_novel_T006261, BGI_novel_T006267, BGI_novel_T006265, BGI_novel_T006259, BGI_novel_T006266, BGI_novel_T006260, BGI_novel_T006258, BGI_novel_T006263 | 9.19E-03 | 1.63E-02 | 7.01E-01 |
| 622 | LOC113806181 | 0.99 | 0.35 | 0.41 | XM_027357305.1 | 2.56E-02 | 4.51E-02 | 9.73E-01 |
| 623 | LOC113821687 | 1.85 | 0.47 | 0.88 | XM_027374205.1 | 3.20E-03 | 3.34E-03 | 4.24E-01 |
| 624 | LOC113830582 | 4.23 | 0.48 | 6.57 | XM_027383786.1 | 1.34E-03 | 9.42E-01 | 1.69E-02 |
| 625 | LOC113818099 | 21.23 | 6.82 | 9.80 | BGI_novel_T000303, BGI_novel_T000301, XM_027370240.1, XM_027370242.1, BGI_novel_T000302 | 1.14E-03 | 1.20E-03 | 4.89E-01 |
| 626 | BGI_novel_G000949 | 57.40 | 20.32 | 30.27 | BGI_novel_T005658 | 8.37E-03 | 5.08E-04 | 5.56E-01 |
| 627 | LOC113818801 | 0.25 | 0.21 | 0.73 | XM_027370991.1 | 6.67E-01 | 1.50E-01 | 4.86E-02 |
| 628 | LOC113809105 | 17.85 | 11.86 | 12.00 | XM_027360592.1 | 6.63E-03 | 1.76E-03 | 4.14E-01 |
| 629 | LOC113814719 | 19.94 | 16.02 | 17.88 | XM_027366769.1 | 1.27E-01 | 4.60E-02 | 9.11E-01 |
| 630 | LOC113823776 | 2.01 | 1.24 | 2.52 | XM_027376465.1 | 7.91E-03 | 6.27E-01 | 6.17E-02 |
| 631 | LOC113814963 | 4.96 | 3.33 | 10.17 | XM_027367043.1 | 1.80E-02 | 2.07E-01 | 2.40E-03 |
| 632 | LOC113805465 | 0.05 | 0.01 | 1.20 | XM_027356467.1 | 5.44E-01 | 1.07E-02 | 6.35E-03 |
| 633 | LOC113823552 | 0.55 | 0.13 | 2.51 | XM_027376217.1 | 7.22E-03 | 1.31E-01 | 1.95E-03 |
| 634 | LOC113806367 | 40.30 | 5.38 | 12.51 | XM_027357493.1, BGI_novel_T002104, BGI_novel_T002094, BGI_novel_T002105, BGI_novel_T002090, BGI_novel_T002093, BGI_novel_T002092, BGI_novel_T002099, BGI_novel_T002101, BGI_novel_T002095, BGI_novel_T002107, BGI_novel_T002100, BGI_novel_T002089, BGI_novel_T002091, BGI_novel_T002106, BGI_novel_T002103, BGI_novel_T002102, BGI_novel_T002096 | 3.12E-03 | 3.42E-01 | 8.21E-02 |
| 635 | BGI_novel_G001298 | 0.71 | 0.56 | 1.40 | BGI_novel_T007863 | 2.51E-01 | 2.91E-01 | 4.10E-02 |
| 636 | LOC113828003 | 18.18 | 8.69 | 14.26 | XM_027380944.1 | 3.31E-04 | 6.45E-02 | 2.37E-01 |
| 637 | LOC113802174 | 2.21 | 1.07 | 7.14 | XM_027352695.1 | 3.23E-03 | 2.08E-01 | 9.15E-03 |
| 638 | LOC113816267 | 5.74 | 2.81 | 19.69 | XM_027368306.1 | 4.02E-02 | 2.59E-01 | 2.31E-02 |
| 639 | BGI_novel_G001152 | 1.98 | 1.37 | 1.50 | BGI_novel_T007036 | 1.10E-01 | 4.63E-02 | 7.82E-01 |
| 640 | BGI_novel_G000763 | 2.07 | 0.47 | 0.93 | BGI_novel_T004713 | 3.01E-03 | 9.41E-02 | 6.11E-01 |
| 641 | LOC113810103 | 0.13 | 0.07 | 0.19 | BGI_novel_T003383, XM_027361786.1 | 4.84E-02 | 4.91E-01 | 3.79E-03 |
| 642 | LOC113823884 | 3.51 | 2.92 | 3.01 | BGI_novel_T007346, XM_027376606.1 | 1.73E-01 | 1.72E-02 | 5.74E-01 |
| 643 | LOC113818779 | 5.07 | 3.00 | 4.26 | XM_027370964.1 | 4.11E-03 | 6.50E-02 | 4.60E-01 |
| 644 | LOC113813188 | 1.32 | 0.62 | 1.23 | BGI_novel_T004273, XM_027365143.1, XM_027365144.1 | 9.37E-03 | 2.03E-01 | 4.94E-01 |
| 645 | LOC113826330 | 1635.94 | 535.14 | 991.40 | XM_027379199.1 | 6.04E-04 | 9.34E-03 | 1.55E-01 |
| 646 | LOC113828356 | 1932.23 | 997.87 | 1400.39 | XM_027381300.1 | 2.79E-03 | 2.40E-03 | 5.34E-01 |
| 647 | LOC113808708 | 2.97 | 2.02 | 2.75 | XM_027360183.1, XM_027360184.1 | 1.47E-02 | 9.81E-02 | 5.44E-01 |
| 648 | LOC113819368 | 31.99 | 10.28 | 27.62 | XM_027371604.1 | 3.72E-02 | 3.00E-01 | 3.27E-01 |
| 649 | LOC113818376 | 1.12 | 0.71 | 1.86 | XM_027370592.1, BGI_novel_T000559 | 4.96E-02 | 6.30E-01 | 1.15E-02 |
| 650 | LOC113810090 | 18.23 | 17.79 | 19.00 | XM_027361776.1 | 4.40E-01 | 3.06E-02 | 3.71E-01 |
| 651 | LOC113828966 | 2.52 | 2.20 | 2.63 | XM_027382039.1, BGI_novel_T008882 | 1.92E-01 | 3.99E-02 | 9.81E-01 |
| 652 | LOC113828750 | 94.30 | 31.88 | 36.86 | BGI_novel_T008741, XM_027381761.1, BGI_novel_T008740 | 1.28E-01 | 4.79E-02 | 7.07E-01 |
| 653 | LOC113828744 | 86.18 | 10.47 | 88.16 | XM_027381753.1 | 5.38E-04 | 8.42E-01 | 1.44E-03 |
| 654 | LOC113806100 | 2.01 | 0.72 | 2.75 | XM_027357208.1, BGI_novel_T002021 | 1.88E-06 | 9.64E-01 | 2.32E-05 |
| 655 | LOC113828747 | 9.07 | 0.00 | 17.27 | XM_027381756.1, BGI_novel_T008746, XM_027381757.1 | 5.50E-04 | 5.79E-01 | 9.75E-04 |
| 656 | LOC113826615 | 0.16 | 0.06 | 0.28 | XM_027379506.1 | 1.20E-01 | 5.26E-01 | 1.64E-02 |
| 657 | LOC113806821 | 47.30 | 24.84 | 33.18 | XM_027357979.1 | 2.34E-02 | 1.53E-02 | 7.67E-01 |
| 658 | LOC113813971 | 0.85 | 0.45 | 0.68 | XM_027366057.1 | 2.23E-03 | 3.83E-02 | 4.58E-01 |
| 659 | LOC113805073 | 0.21 | 0.16 | 0.97 | XM_027355985.1, BGI_novel_T000165 | 8.24E-01 | 2.55E-02 | 5.84E-02 |
| 660 | LOC113826598 | 67.49 | 46.23 | 80.68 | XM_027379493.1, BGI_novel_T000853, BGI_novel_T000842, BGI_novel_T000855, BGI_novel_T000852 | 1.98E-02 | 7.00E-01 | 1.04E-01 |
| 661 | LOC113825442 | 13.23 | 11.74 | 14.02 | XM_027378276.1, BGI_novel_T007802, XM_027378275.1, BGI_novel_T007801, BGI_novel_T007803 | 4.34E-02 | 2.08E-01 | 7.16E-01 |
| 662 | LOC113819420 | 0.71 | 0.04 | 0.42 | BGI_novel_T006029, XM_027371659.1 | 4.22E-02 | 4.93E-01 | 3.06E-01 |
| 663 | LOC113816958 | 301.34 | 189.09 | 228.26 | XM_027368950.1, XM_027368951.1 | 7.08E-02 | 4.89E-05 | 8.85E-01 |
| 664 | LOC113820593 | 78.54 | 33.38 | 48.93 | XM_027372936.1 | 2.28E-04 | 4.49E-04 | 4.11E-01 |
| 665 | LOC113811796 | 2.92 | 2.16 | 2.72 | BGI_novel_T003905, BGI_novel_T003907, XM_027363615.1, BGI_novel_T003904, BGI_novel_T003903, BGI_novel_T003906 | 4.76E-02 | 6.89E-02 | 8.67E-01 |
| 666 | LOC113828244 | 2.62 | 1.71 | 2.25 | BGI_novel_T008614, XM_027381181.1 | 3.04E-02 | 1.32E-04 | 7.90E-01 |
| 667 | LOC113800347 | 25.31 | 13.54 | 22.58 | BGI_novel_T009430, XM_027351102.1, XM_027351103.1, XM_027351101.1, XM_027351100.1, XM_027351104.1 | 1.89E-02 | 3.35E-02 | 2.38E-01 |
| 668 | LOC113817392 | 8.38 | 4.60 | 6.32 | XM_027369432.1 | 5.82E-07 | 4.98E-04 | 4.22E-01 |
| 669 | LOC113808562 | 26.30 | 21.43 | 38.28 | XM_027359992.1 | 4.13E-02 | 8.29E-01 | 3.39E-02 |
| 670 | LOC113823306 | 6.36 | 6.18 | 15.43 | XM_027375921.1 | 9.99E-01 | 8.13E-02 | 2.00E-02 |
| 671 | LOC113823307 | 98.97 | 43.85 | 84.70 | XM_027375922.1 | 6.01E-04 | 1.01E-01 | 1.05E-01 |
| 672 | LOC113812827 | 10.32 | 8.55 | 9.59 | XM_027364745.1 | 3.12E-01 | 7.55E-03 | 5.55E-01 |
| 673 | LOC113817472 | 69.98 | 42.75 | 79.77 | XM_027369540.1 | 3.11E-02 | 4.24E-01 | 3.84E-01 |
| 674 | LOC113827012 | 313.73 | 184.06 | 271.17 | XM_027379918.1 | 2.67E-02 | 4.15E-01 | 4.26E-01 |
| 675 | LOC113809487 | 0.81 | 0.00 | 0.38 | XM_027361101.1, BGI_novel_T003223 | 5.98E-03 | 3.76E-01 | 1.37E-01 |
| 676 | LOC113816640 | 0.10 | 0.09 | 0.30 | XM_027368695.1 | 5.84E-01 | 6.71E-02 | 1.74E-02 |
| 677 | LOC113820782 | 3.72 | 1.78 | 3.00 | XM_027373145.1 | 3.21E-02 | 5.96E-02 | 3.12E-01 |
| 678 | LOC113826162 | 21.97 | 9.83 | 20.66 | XM_027379034.1, XM_027379035.1 | 5.04E-03 | 1.55E-01 | 1.32E-01 |
| 679 | BGI_novel_G001425 | 1.29 | 0.70 | 2.16 | BGI_novel_T008602 | 4.77E-02 | 7.89E-01 | 5.39E-02 |
| 680 | LOC113817457 | 0.11 | 0.01 | 0.16 | XM_027369527.1 | 2.83E-02 | 9.33E-01 | 5.12E-02 |
| 681 | LOC113825509 | 8.40 | 3.46 | 3.91 | XM_027378350.1 | 1.19E-03 | 3.18E-03 | 8.02E-01 |
| 682 | LOC113828765 | 6.57 | 2.14 | 2.28 | XM_027381778.1, XM_027381779.1, XM_027381777.1, XM_027381780.1 | 4.72E-02 | 1.14E-04 | 7.20E-01 |
| 683 | BGI_novel_G000086 | 1.25 | 0.62 | 4.15 | BGI_novel_T000491 | 1.03E-01 | 1.16E-01 | 1.22E-02 |
| 684 | LOC113801069 | 156.36 | 96.92 | 126.24 | XM_027351881.1, XM_027351879.1, XM_027351882.1, XM_027351880.1, BGI_novel_T009618, XM_027351877.1, BGI_novel_T009619, XM_027351884.1, XM_027351883.1, BGI_novel_T009617 | 1.26E-01 | 2.17E-02 | 7.79E-01 |
| 685 | LOC113805179 | 1.20 | 0.36 | 0.74 | XM_027356133.1, XM_027356136.1, XM_027356135.1, XM_027356134.1 | 1.36E-02 | 1.10E-01 | 1.78E-01 |
| 686 | LOC113799965 | 0.60 | 0.27 | 0.87 | XM_027350674.1 | 4.59E-02 | 5.98E-01 | 1.38E-02 |
| 687 | LOC113813287 | 6.44 | 4.54 | 6.02 | XM_027365255.1 | 8.46E-03 | 5.35E-02 | 6.37E-01 |
| 688 | LOC113820467 | 0.14 | 0.08 | 0.46 | XM_027372783.1 | 8.90E-02 | 2.28E-02 | 2.40E-04 |
| 689 | LOC113816016 | 7.51 | 6.50 | 7.92 | XM_027368036.1, BGI_novel_T005075 | 3.99E-02 | 9.08E-03 | 8.73E-01 |
| 690 | LOC113828556 | 1.32 | 0.35 | 0.81 | XM_027381560.1 | 1.51E-02 | 1.27E-01 | 3.37E-01 |
| 691 | LOC113807661 | 74.00 | 40.80 | 46.24 | XM_027358966.1, BGI_novel_T002563 | 6.35E-05 | 7.10E-03 | 8.50E-01 |
| 692 | LOC113829236 | 0.29 | 0.11 | 0.50 | XM_027382354.1, XM_027382353.1, XM_027382352.1 | 1.12E-01 | 5.80E-01 | 6.77E-03 |
| 693 | LOC113820552 | 6.87 | 3.40 | 4.86 | BGI_novel_T000602, XM_027372904.1, BGI_novel_T000601, XM_027372908.1, XM_027372905.1, BGI_novel_T000603 | 4.15E-04 | 3.15E-03 | 2.92E-01 |
| 694 | BGI_novel_G000557 | 0.50 | 0.32 | 1.12 | BGI_novel_T003338 | 2.57E-01 | 3.79E-01 | 1.39E-02 |
| 695 | LOC113804121 | 12.36 | 8.87 | 15.55 | BGI_novel_T001370, BGI_novel_T001371, XM_027354958.1 | 1.57E-03 | 8.44E-01 | 3.85E-02 |
| 696 | LOC113816942 | 10.71 | 5.75 | 6.47 | XM_027368934.1 | 7.08E-02 | 3.44E-02 | 8.55E-01 |
| 697 | LOC113818204 | 0.22 | 0.18 | 0.56 | XM_027370381.1 | 3.77E-01 | 3.83E-02 | 1.43E-02 |
| 698 | LOC113805450 | 27.47 | 7.49 | 10.25 | XM_027356443.1 | 4.38E-03 | 2.65E-03 | 8.14E-01 |
| 699 | LOC113825640 | 1.88 | 0.89 | 2.82 | XM_027378486.1 | 3.24E-02 | 6.85E-01 | 1.62E-02 |
| 700 | LOC113813947 | 3.80 | 0.78 | 2.73 | XM_027366026.1 | 1.48E-03 | 9.65E-02 | 2.03E-01 |
| 701 | LOC113815488 | 0.17 | 0.02 | 0.31 | XM_027367552.1 | 9.45E-02 | 5.57E-01 | 7.86E-03 |
| 702 | LOC113823724 | 2.95 | 1.59 | 1.68 | XM_027376413.1, XM_027376414.1 | 3.74E-02 | 1.80E-02 | 8.79E-01 |
| 703 | LOC113828360 | 18.81 | 14.30 | 15.73 | XM_027381309.1, XM_027381308.1, XM_027381310.1, XM_027381311.1, BGI_novel_T008641 | 1.06E-02 | 1.80E-01 | 9.80E-01 |
| 704 | LOC113804714 | 123.92 | 55.11 | 105.00 | XM_027355601.1 | 1.36E-02 | 2.03E-01 | 1.70E-01 |
| 705 | LOC113819748 | 0.93 | 0.13 | 0.78 | XM_027371939.1, BGI_novel_T006119, XM_027371941.1 | 4.25E-03 | 5.43E-01 | 1.15E-01 |
| 706 | LOC113817568 | 10.19 | 8.90 | 10.70 | BGI_novel_T005516, XM_027369610.1 | 4.17E-02 | 9.44E-02 | 6.59E-01 |
| 707 | LOC113813933 | 54.18 | 30.95 | 52.89 | XM_027366019.1 | 2.10E-03 | 3.76E-01 | 1.49E-01 |
| 708 | LOC113825881 | 1.90 | 0.28 | 0.34 | XM_027378726.1 | 1.51E-04 | 9.70E-04 | 8.42E-01 |
| 709 | LOC113822192 | 19.13 | 6.78 | 10.16 | XM_027374733.1, BGI_novel_T006800 | 3.68E-02 | 1.09E-01 | 6.68E-01 |
| 710 | LOC113813209 | 0.58 | 0.34 | 0.67 | XM_027365169.1 | 2.99E-02 | 3.77E-01 | 1.48E-01 |
| 711 | LOC113812988 | 84.88 | 55.64 | 64.97 | XM_027364929.1 | 9.15E-04 | 9.13E-05 | 8.55E-01 |
| 712 | LOC113824685 | 47.25 | 17.20 | 34.81 | BGI_novel_T007560, XM_027377455.1, XM_027377454.1, XM_027377453.1 | 7.35E-03 | 4.22E-02 | 2.02E-01 |
| 713 | LOC113829845 | 2.95 | 2.43 | 2.87 | BGI_novel_T000954, XM_027383024.1 | 2.18E-01 | 4.42E-02 | 8.08E-01 |
| 714 | LOC113807020 | 7.24 | 6.50 | 7.52 | BGI_novel_T002289, BGI_novel_T002290, XM_027358175.1 | 1.63E-01 | 4.58E-03 | 8.82E-01 |
| 715 | BGI_novel_G000907 | 1.47 | 0.42 | 0.78 | BGI_novel_T005436 | 2.66E-03 | 2.52E-02 | 2.33E-01 |
| 716 | LOC113818640 | 2.50 | 0.77 | 3.78 | XM_027370829.1 | 7.39E-02 | 8.37E-01 | 4.74E-02 |
| 717 | LOC113818642 | 3.92 | 1.98 | 5.99 | BGI_novel_T005776, XM_027370830.1 | 1.21E-02 | 8.63E-01 | 2.63E-02 |
| 718 | LOC113827925 | 3.11 | 2.34 | 5.36 | BGI_novel_T002920, XM_027380878.1 | 3.12E-01 | 3.64E-01 | 3.20E-02 |
| 719 | LOC113810372 | 22.77 | 16.96 | 17.78 | BGI_novel_T003454, XM_027362065.1, BGI_novel_T003453, XM_027362064.1 | 7.52E-03 | 5.33E-02 | 7.78E-01 |
| 720 | LOC113824000 | 29.78 | 23.36 | 29.03 | XM_027376739.1, XM_027376740.1, XM_027376737.1, XM_027376741.1, XM_027376738.1, XM_027376736.1, BGI_novel_T007390 | 8.10E-04 | 6.23E-02 | 7.77E-01 |
| 721 | BGI_novel_G000677 | 7.08 | 1.30 | 4.19 | BGI_novel_T004061 | 7.03E-05 | 6.38E-02 | 1.28E-02 |
| 722 | LOC113812358 | 26.30 | 17.39 | 18.27 | XM_027364232.1, BGI_novel_T000664, BGI_novel_T000665 | 1.39E-02 | 1.95E-02 | 9.59E-01 |
| 723 | LOC113809157 | 38.89 | 14.23 | 29.06 | BGI_novel_T003081, BGI_novel_T003080, XM_027360656.1 | 2.23E-02 | 4.49E-02 | 2.52E-01 |
| 724 | LOC113805351 | 0.85 | 0.69 | 1.59 | XM_027356345.1 | 2.03E-01 | 2.48E-01 | 3.24E-02 |
| 725 | LOC113809654 | 0.64 | 0.58 | 1.43 | XM_027361347.1, BGI_novel_T000027, BGI_novel_T000028 | 4.39E-01 | 6.20E-02 | 3.78E-02 |
| 726 | LOC113806423 | 1.55 | 1.35 | 2.45 | XM_027357537.1, XM_027357536.1, XM_027357535.1 | 1.61E-01 | 2.38E-01 | 1.11E-02 |
| 727 | LOC113828909 | 114.26 | 48.65 | 109.44 | XM_027381969.1 | 2.71E-03 | 1.35E-01 | 3.39E-02 |
| 728 | LOC113822977 | 2.20 | 0.76 | 2.89 | BGI_novel_T007021, BGI_novel_T007020, XM_027375528.1 | 3.46E-03 | 5.60E-01 | 1.29E-02 |
| 729 | LOC113829621 | 2.88 | 2.76 | 2.90 | XM_027382819.1, XM_027382820.1, XM_027382821.1, BGI_novel_T009058 | 1.98E-01 | 1.05E-03 | 2.32E-01 |
| 730 | LOC113820850 | 0.11 | 0.04 | 0.04 | XM_027373227.1 | 6.62E-02 | 1.66E-02 | 7.87E-01 |
| 731 | LOC113828252 | 3.54 | 0.71 | 2.00 | BGI_novel_T008616, XM_027381190.1 | 3.37E-05 | 1.74E-03 | 7.69E-02 |
| 732 | LOC113814597 | 246.03 | 94.69 | 131.19 | BGI_novel_T004716, XM_027366643.1, BGI_novel_T004715 | 5.61E-03 | 4.18E-02 | 5.53E-01 |
| 733 | BGI_novel_G001079 | 37.26 | 9.65 | 18.27 | BGI_novel_T006469 | 2.43E-04 | 1.17E-02 | 2.22E-01 |
| 734 | LOC113820982 | 152.19 | 42.06 | 78.21 | XM_027373400.1, XM_027373397.1, XM_027373402.1, XM_027373399.1, XM_027373404.1, XM_027373401.1, XM_027373403.1, XM_027373398.1 | 2.62E-04 | 8.27E-03 | 2.05E-01 |
| 735 | LOC113810954 | 108.95 | 24.46 | 75.25 | BGI_novel_T003685, BGI_novel_T003684, XM_027362612.1 | 1.03E-07 | 3.77E-02 | 3.49E-04 |
| 736 | LOC113826882 | 38.51 | 21.50 | 35.26 | XM_027379785.1 | 2.60E-02 | 2.22E-01 | 3.81E-01 |
| 737 | LOC113823154 | 22.26 | 8.63 | 11.18 | BGI_novel_T000314, BGI_novel_T000315, XM_027375770.1 | 1.01E-03 | 6.27E-03 | 7.21E-01 |
| 738 | LOC113810566 | 43.93 | 21.05 | 26.97 | XM_027362205.1, XM_027362213.1, XM_027362191.1, XM_027362200.1 | 1.34E-03 | 3.57E-03 | 6.34E-01 |
| 739 | LOC113823152 | 276.98 | 88.98 | 100.53 | BGI_novel_T007091, BGI_novel_T007093, BGI_novel_T007096, BGI_novel_T007087, BGI_novel_T007089, BGI_novel_T007095, BGI_novel_T007092, BGI_novel_T007090, BGI_novel_T007088, XM_027375769.1, BGI_novel_T007086, BGI_novel_T007094, BGI_novel_T007097 | 1.52E-03 | 1.17E-02 | 5.28E-01 |
| 740 | LOC113820984 | 54.42 | 23.44 | 40.26 | XM_027373405.1 | 1.92E-02 | 8.05E-02 | 3.74E-01 |
| 741 | LOC113822606 | 136.47 | 27.89 | 79.55 | BGI_novel_T006901, XM_027375137.1, XM_027375136.1 | 1.73E-08 | 9.65E-03 | 2.49E-03 |
| 742 | LOC113806683 | 0.57 | 0.13 | 0.23 | XM_027357847.1 | 2.81E-02 | 7.50E-02 | 4.58E-01 |
| 743 | LOC113810209 | 3.67 | 0.56 | 1.27 | XM_027361921.1, BGI_novel_T003396 | 1.90E-07 | 7.50E-03 | 3.12E-01 |
| 744 | LOC113800965 | 38.88 | 13.17 | 49.12 | XM_027351776.1, XM_027351775.1 | 3.59E-03 | 7.73E-01 | 1.26E-02 |
| 745 | LOC113823251 | 16.37 | 10.40 | 13.38 | XM_027375903.1, BGI_novel_T000068 | 4.11E-03 | 1.16E-02 | 5.89E-01 |
| 746 | LOC113804724 | 388.85 | 298.95 | 314.64 | XM_027355617.1 | 2.36E-01 | 4.23E-02 | 1.23E-01 |
| 747 | LOC113800581 | 35.16 | 22.25 | 43.28 | XM_027351374.1 | 4.07E-03 | 8.55E-01 | 2.53E-02 |
| 748 | LOC113811192 | 1.56 | 1.22 | 1.37 | XM_027362873.1 | 2.00E-02 | 4.72E-02 | 9.17E-01 |
| 749 | LOC113820539 | 18.81 | 8.46 | 13.50 | XM_027372899.1 | 7.32E-04 | 3.89E-04 | 2.66E-01 |
| 750 | LOC113816678 | 6.38 | 3.36 | 5.29 | XM_027368737.1, BGI_novel_T005297, BGI_novel_T005298 | 1.46E-02 | 1.73E-01 | 5.41E-01 |
| 751 | LOC113809642 | 0.04 | 0.02 | 0.12 | XM_027361294.1 | 6.59E-01 | 3.43E-01 | 3.62E-02 |
| 752 | LOC113820109 | 0.01 | 0.00 | 0.09 | XM_027372376.1 | 6.72E-01 | 1.47E-01 | 3.85E-02 |
| 753 | LOC113819227 | 0.26 | 0.13 | 0.71 | XM_027371477.1, BGI_novel_T005982, BGI_novel_T005981 | 1.36E-01 | 9.85E-02 | 3.54E-03 |
| 754 | LOC113803235 | 8.90 | 5.28 | 7.29 | XM_027353957.1, XM_027353956.1 | 8.36E-03 | 4.07E-02 | 4.56E-01 |
| 755 | LOC113803798 | 32.74 | 23.67 | 36.00 | BGI_novel_T001276, XM_027354622.1 | 2.66E-02 | 1.72E-01 | 2.67E-01 |
| 756 | LOC113800807 | 4.08 | 2.95 | 4.91 | XM_027351596.1, XM_027351594.1, XM_027351595.1, XM_027351593.1 | 1.75E-02 | 5.16E-01 | 2.54E-02 |
| 757 | LOC113809191 | 2.52 | 1.58 | 4.10 | BGI_novel_T003089, XM_027360717.1 | 3.94E-02 | 6.93E-01 | 2.52E-02 |
| 758 | LOC113829288 | 8.00 | 3.66 | 7.23 | BGI_novel_T008994, XM_027382425.1 | 1.11E-03 | 1.17E-01 | 5.33E-02 |
| 759 | LOC113807426 | 19.83 | 11.21 | 18.98 | BGI_novel_T002447, XM_027358684.1, XM_027358683.1, XM_027358682.1, XM_027358681.1, XM_027358687.1, XM_027358679.1, XM_027358685.1, XM_027358686.1,XM_027358680.1 | 2.00E-02 | 1.66E-01 | 2.57E-01 |
| 760 | LOC113811646 | 2.77 | 2.56 | 2.71 | XM_027363428.1 | 2.70E-01 | 1.63E-02 | 3.13E-01 |
| 761 | LOC113812120 | 48.09 | 30.67 | 51.22 | BGI_novel_T003975, BGI_novel_T003984, BGI_novel_T003967, XM_027363951.1, BGI_novel_T003980, BGI_novel_T003972, BGI_novel_T003988, BGI_novel_T003976, BGI_novel_T003968, BGI_novel_T003985, BGI_novel_T003973, BGI_novel_T003982, BGI_novel_T003989, BGI_novel_T003977, BGI_novel_T003993, BGI_novel_T003986, BGI_novel_T003970, BGI_novel_T003969, BGI_novel_T003992, BGI_novel_T003995, BGI_novel_T003978, BGI_novel_T003987, BGI_novel_T003971, BGI_novel_T003981, BGI_novel_T003991, BGI_novel_T003979, BGI_novel_T003994, BGI_novel_T003990, BGI_novel_T003983, BGI_novel_T003974, BGI_novel_T003966 | 1.74E-02 | 2.09E-01 | 1.75E-01 |
| 762 | LOC113808041 | 0.37 | 0.17 | 0.18 | XM_027359368.1 | 4.02E-02 | 5.80E-03 | 7.29E-01 |
| 763 | LOC113816761 | 159.21 | 83.05 | 229.52 | XM_027368825.1 | 2.04E-01 | 5.40E-01 | 3.08E-02 |
| 764 | LOC113816460 | 0.15 | 0.13 | 0.58 | XM_027368507.1 | 6.78E-01 | 4.00E-02 | 8.12E-03 |
| 765 | LOC113801631 | 6.71 | 5.43 | 6.11 | XM_027352189.1 | 3.03E-02 | 1.56E-01 | 8.30E-01 |
| 766 | LOC113819008 | 182.01 | 67.38 | 101.82 | XM_027371261.1, BGI_novel_T005935 | 1.57E-04 | 1.64E-02 | 4.36E-01 |
| 767 | LOC113830106 | 3.91 | 1.46 | 2.56 | XM_027383294.1, BGI_novel_T009152 | 1.20E-03 | 1.92E-02 | 1.15E-01 |
| 768 | LOC113800661 | 5.33 | 3.80 | 8.23 | BGI_novel_T009506, BGI_novel_T009507, XM_027351469.1 | 3.26E-02 | 7.75E-01 | 8.34E-02 |
| 769 | LOC113809215 | 74.07 | 55.00 | 77.79 | XM_027360745.1 | 4.03E-02 | 1.46E-01 | 3.72E-01 |
| 770 | LOC113826338 | 18.58 | 12.48 | 18.91 | XM_027379209.1, BGI_novel_T008078 | 1.72E-02 | 3.33E-02 | 2.27E-01 |
| 771 | LOC113817581 | 436.12 | 263.21 | 274.27 | XM_027369629.1, XM_027369628.1 | 4.44E-02 | 1.05E-02 | 7.95E-01 |
| 772 | LOC113818707 | 3.38 | 2.15 | 2.84 | XM_027370891.1 | 3.18E-02 | 3.37E-02 | 5.41E-01 |
| 773 | LOC113807145 | 2.23 | 0.75 | 1.55 | BGI_novel_T002368, XM_027358351.1 | 2.25E-02 | 6.56E-02 | 3.13E-01 |
| 774 | LOC113828137 | 109.56 | 65.94 | 82.70 | XM_027381074.1, XM_027381073.1, BGI_novel_T006768, BGI_novel_T006767, BGI_novel_T006766 | 1.14E-02 | 1.15E-02 | 6.85E-01 |
| 775 | LOC113803886 | 3.63 | 3.03 | 3.56 | XM_027354698.1, BGI_novel_T001312, BGI_novel_T001310, BGI_novel_T001311 | 4.35E-02 | 6.17E-02 | 8.13E-01 |
| 776 | LOC113817047 | 6.17 | 5.03 | 6.10 | XM_027369048.1, BGI_novel_T005351, XM_027369047.1, XM_027369046.1 | 1.62E-01 | 1.58E-02 | 8.65E-01 |
| 777 | LOC113804155 | 2.16 | 1.29 | 1.66 | XM_027354994.1, XM_027354993.1 | 1.10E-03 | 4.02E-02 | 6.12E-01 |
| 778 | LOC113803441 | 0.17 | 0.08 | 0.23 | XM_027354221.1 | 7.97E-02 | 8.16E-01 | 3.02E-02 |
| 779 | LOC113824799 | 5.30 | 4.86 | 5.15 | XM_027377587.1, BGI_novel_T007655, XM_027377588.1 | 2.47E-01 | 2.85E-02 | 5.63E-01 |
| 780 | LOC113810828 | 1.09 | 0.19 | 1.88 | XM_027362475.1, BGI_novel_T003610 | 2.35E-02 | 9.80E-01 | 9.85E-02 |
| 781 | LOC113816982 | 1.46 | 1.23 | 2.26 | XM_027368974.1, BGI_novel_T005336 | 4.37E-02 | 4.40E-01 | 9.56E-03 |
| 782 | LOC113816541 | 16.33 | 12.33 | 27.01 | BGI_novel_T005272, XM_027368575.1, BGI_novel_T005273, XM_027368573.1, BGI_novel_T005274, BGI_novel_T005271 | 2.05E-01 | 3.46E-01 | 3.28E-02 |
| 783 | LOC113816991 | 0.03 | 0.00 | 0.42 | XM_027368983.1 | 3.52E-01 | 3.28E-02 | 2.72E-03 |
| 784 | LOC113822210 | 0.17 | 0.04 | 0.11 | XM_027374748.1 | 1.87E-02 | 2.07E-01 | 2.09E-01 |
| 785 | LOC113804876 | 0.27 | 0.08 | 0.67 | XM_027355787.1 | 4.00E-01 | 4.60E-01 | 1.69E-02 |
| 786 | LOC113823594 | 4.74 | 3.28 | 5.50 | XM_027376274.1, XM_027376275.1 | 1.22E-02 | 3.07E-01 | 1.18E-01 |
| 787 | LOC113803144 | 0.55 | 0.35 | 0.89 | XM_027353870.1 | 2.20E-03 | 6.48E-01 | 5.63E-03 |
| 788 | LOC113804085 | 178.27 | 113.11 | 181.12 | BGI_novel_T001352, BGI_novel_T001351, XM_027354916.1 | 2.96E-02 | 3.08E-01 | 2.55E-01 |
| 789 | LOC113823681 | 0.01 | 0.00 | 0.10 | XM_027376360.1 | 7.01E-01 | 1.79E-02 | 3.68E-03 |
| 790 | LOC113828456 | 0.09 | 0.06 | 0.09 | XM_027381440.1, XM_027381441.1 | 3.64E-02 | 2.53E-01 | 3.75E-01 |
| 791 | LOC113812900 | 0.08 | 0.07 | 0.41 | XM_027364823.1 | 7.88E-01 | 2.21E-02 | 3.59E-02 |
| 792 | LOC113829437 | 0.21 | 0.03 | 0.70 | XM_027382611.1 | 2.19E-01 | 4.03E-01 | 2.49E-02 |
| 793 | LOC113808527 | 32.11 | 18.30 | 26.43 | XM_027359955.1, BGI_novel_T002830, XM_027359952.1 | 7.04E-07 | 6.89E-03 | 3.34E-01 |
| 794 | LOC113816495 | 2.60 | 1.75 | 3.91 | XM_027368534.1 | 1.81E-02 | 5.94E-01 | 1.10E-02 |
| 795 | LOC113829828 | 10.20 | 5.33 | 7.34 | XM_027383001.1 | 5.04E-04 | 6.47E-04 | 4.36E-01 |
| 796 | LOC113802445 | 6.48 | 3.43 | 5.31 | XM_027353021.1, BGI_novel_T009937 | 2.66E-02 | 1.34E-02 | 3.88E-01 |
| 797 | LOC113811066 | 14.58 | 7.49 | 9.37 | XM_027362737.1 | 1.43E-01 | 4.35E-03 | 9.96E-01 |
| 798 | LOC113814477 | 0.35 | 0.27 | 1.63 | XM_027366508.1, XM_027366507.1, XM_027366506.1 | 5.01E-01 | 9.67E-02 | 8.28E-03 |
| 799 | LOC113827557 | 1.26 | 1.16 | 3.29 | XM_027380447.1, XM_027380448.1 | 5.21E-01 | 6.79E-03 | 4.25E-02 |
| 800 | LOC113827171 | 154.14 | 87.05 | 136.29 | XM_027380069.1 | 1.16E-02 | 1.37E-01 | 3.25E-01 |
| 801 | LOC113808462 | 112.48 | 100.00 | 115.07 | XM_027359880.1 | 2.87E-01 | 1.98E-02 | 6.78E-01 |
| 802 | LOC113821398 | 205.41 | 39.46 | 143.05 | XM_027373880.1 | 1.48E-08 | 6.11E-02 | 1.30E-03 |
| 803 | LOC113829837 | 0.68 | 0.35 | 1.23 | XM_027383014.1 | 2.34E-03 | 3.23E-01 | 4.40E-04 |
| 804 | LOC113814814 | 0.48 | 0.27 | 0.94 | XM_027366870.1 | 7.96E-02 | 4.22E-01 | 3.27E-02 |
| 805 | LOC113814821 | 0.12 | 0.07 | 0.39 | XM_027366883.1 | 2.67E-01 | 1.36E-01 | 2.23E-02 |
| 806 | LOC113815140 | 0.04 | 0.00 | 0.08 | XM_027367237.1 | 3.17E-02 | 7.50E-01 | 3.79E-02 |
| 807 | LOC113830101 | 0.75 | 0.44 | 1.56 | BGI_novel_T009153, XM_027383290.1 | 8.21E-02 | 4.56E-01 | 1.19E-02 |
| 808 | LOC113810773 | 0.11 | 0.10 | 0.44 | XM_027362418.1, XM_027362420.1 | 5.99E-01 | 1.37E-02 | 5.74E-03 |
| 809 | LOC113823563 | 20.12 | 3.85 | 20.87 | XM_027376233.1 | 2.81E-03 | 6.27E-01 | 1.06E-03 |
| 810 | LOC113818953 | 2.94 | 2.10 | 2.54 | BGI_novel_T005887, XM_027371176.1 | 4.96E-02 | 9.16E-03 | 8.36E-01 |
| 811 | LOC113829346 | 12.60 | 7.74 | 9.43 | XM_027382501.1 | 1.94E-02 | 2.53E-02 | 8.94E-01 |
| 812 | LOC113820005 | 497.01 | 269.48 | 465.54 | XM_027372259.1 | 1.10E-03 | 4.11E-01 | 1.64E-01 |
| 813 | BGI_novel_G000644 | 1.11 | 0.10 | 0.15 | BGI_novel_T003892 | 2.42E-04 | 1.70E-02 | 6.82E-01 |
| 814 | LOC113828511 | 16.86 | 13.57 | 15.40 | XM_027381497.1, XM_027381496.1, XM_027381495.1 | 7.17E-02 | 1.87E-02 | 6.10E-01 |
| 815 | LOC113823595 | 1.46 | 0.87 | 1.31 | XM_027376279.1 | 1.81E-02 | 9.22E-02 | 3.41E-01 |
| 816 | LOC113803149 | 3.81 | 2.17 | 2.60 | XM_027353873.1 | 7.97E-02 | 4.30E-03 | 9.03E-01 |
| 817 | LOC113813189 | 1.50 | 1.49 | 1.58 | XM_027365145.1 | 3.47E-01 | 2.27E-02 | 3.65E-01 |
| 818 | LOC113817049 | 1.68 | 1.35 | 3.74 | XM_027369050.1 | 1.03E-01 | 6.06E-03 | 8.41E-04 |
| 819 | LOC113823870 | 0.44 | 0.35 | 1.22 | XM_027376587.1, XM_027376583.1, XM_027376586.1, XM_027376584.1, XM_027376585.1, XM_027376588.1 | 4.59E-01 | 3.34E-02 | 3.58E-02 |
| 820 | LOC113819485 | 4.25 | 3.62 | 3.79 | BGI_novel_T006037, BGI_novel_T006038, BGI_novel_T006039, XM_027371720.1, BGI_novel_T006040 | 3.83E-02 | 1.36E-02 | 9.80E-01 |
| 821 | LOC113816539 | 0.55 | 0.39 | 0.66 | XM_027368570.1 | 1.68E-02 | 3.75E-01 | 1.25E-01 |
| 822 | LOC113823699 | 10.91 | 9.24 | 13.27 | BGI_novel_T007265, XM_027376385.1 | 4.62E-02 | 4.49E-01 | 2.51E-01 |
| 823 | LOC113806697 | 157.16 | 107.61 | 146.66 | XM_027357866.1 | 3.97E-02 | 1.07E-02 | 6.54E-01 |
| 824 | LOC113818431 | 0.11 | 0.02 | 0.15 | XM_027370603.1 | 1.27E-02 | 9.89E-01 | 5.09E-02 |
| 825 | LOC113807843 | 3.49 | 2.26 | 2.75 | XM_027359148.1 | 1.26E-02 | 1.36E-02 | 9.34E-01 |
| 826 | LOC113809677 | 1.48 | 0.66 | 1.08 | XM_027361337.1 | 2.08E-03 | 1.71E-02 | 3.19E-01 |
| 827 | LOC113823564 | 12.39 | 4.55 | 5.34 | XM_027376234.1 | 2.65E-03 | 5.19E-04 | 9.43E-01 |
| 828 | LOC113802175 | 21.38 | 13.38 | 18.65 | XM_027352697.1, XM_027352699.1, XM_027352696.1 | 5.80E-03 | 4.08E-03 | 4.77E-01 |
| 829 | LOC113802177 | 44.34 | 20.81 | 35.59 | XM_027352704.1, XM_027352701.1, XM_027352705.1, XM_027352703.1, BGI_novel_T009853, XM_027352702.1 | 1.28E-03 | 6.90E-03 | 2.03E-01 |
| 830 | LOC113817445 | 9.14 | 5.59 | 6.85 | BGI_novel_T000526, XM_027369524.1, BGI_novel_T000525, XM_027369531.1, XM_027369519.1, BGI_novel_T000523, BGI_novel_T000524 | 1.41E-02 | 1.19E-02 | 8.89E-01 |
| 831 | LOC113817668 | 32.07 | 7.23 | 12.62 | XM_027369748.1, BGI_novel_T000532, BGI_novel_T000529, BGI_novel_T000533, BGI_novel_T000530, BGI_novel_T000527, BGI_novel_T000531 | 3.67E-05 | 3.93E-04 | 2.24E-01 |
| 832 | LOC113808519 | 143.39 | 82.34 | 101.34 | BGI_novel_T002831, BGI_novel_T002840, BGI_novel_T002837, BGI_novel_T002833, BGI_novel_T002839, BGI_novel_T002836, XM_027359946.1, BGI_novel_T002832, BGI_novel_T002838, BGI_novel_T002834, BGI_novel_T002835 | 7.03E-02 | 4.73E-02 | 9.85E-01 |
| 833 | LOC113805049 | 0.42 | 0.28 | 0.67 | XM_027355957.1 | 6.96E-02 | 5.34E-01 | 2.52E-02 |
| 834 | LOC113800579 | 21.67 | 11.13 | 17.47 | XM_027351372.1 | 1.53E-03 | 6.01E-02 | 1.56E-01 |
| 835 | LOC113824079 | 0.18 | 0.05 | 0.18 | XM_027376827.1 | 1.19E-02 | 3.70E-01 | 3.97E-02 |
| 836 | LOC113805431 | 122.19 | 75.60 | 84.73 | XM_027356419.1 | 2.53E-02 | 2.85E-02 | 9.70E-01 |
| 837 | LOC113803004 | 5.29 | 3.14 | 6.30 | XM_027353710.1, BGI_novel_T001049, BGI_novel_T001048 | 3.09E-02 | 4.83E-01 | 5.96E-02 |
| 838 | LOC113827175 | 7.35 | 4.42 | 5.89 | BGI_novel_T008300, XM_027380072.1, XM_027380073.1 | 1.49E-02 | 1.40E-01 | 5.97E-01 |
| 839 | LOC113813885 | 18.75 | 12.65 | 12.66 | XM_027365939.1, BGI_novel_T004508 | 7.31E-03 | 6.16E-04 | 4.61E-01 |
| 840 | LOC113824467 | 2.14 | 1.54 | 1.81 | XM_027377208.1 | 1.90E-02 | 1.58E-02 | 9.52E-01 |
| 841 | LOC113805242 | 2.05 | 1.40 | 2.51 | XM_027356209.1, XM_027356210.1 | 2.24E-02 | 4.57E-01 | 1.04E-01 |
| 842 | LOC113824062 | 14.68 | 10.79 | 13.34 | XM_027376804.1, XM_027376801.1, XM_027376803.1 | 2.02E-01 | 2.16E-02 | 8.68E-01 |
| 843 | LOC113815875 | 13.57 | 13.18 | 16.46 | XM_027367893.1, BGI_novel_T005034 | 3.01E-01 | 4.71E-02 | 4.40E-01 |
| 844 | LOC113812170 | 28.72 | 26.56 | 27.87 | XM_027364007.1, XM_027364006.1, BGI_novel_T004007, BGI_novel_T004008, XM_027364009.1, BGI_novel_T004010, BGI_novel_T004009, XM_027364005.1, BGI_novel_T004011, XM_027364003.1, XM_027364008.1, XM_027364004.1 | 4.21E-01 | 2.21E-02 | 4.54E-01 |
| 845 | LOC113809630 | 0.31 | 0.10 | 0.30 | XM_027361271.1 | 2.66E-02 | 5.91E-01 | 4.31E-01 |
| 846 | LOC113827371 | 40.96 | 29.63 | 55.13 | XM_027380251.1 | 2.25E-04 | 7.69E-01 | 2.88E-03 |
| 847 | LOC113817647 | 44.38 | 39.43 | 52.27 | XM_027369702.1, BGI_novel_T005541, XM_027369703.1, XM_027369701.1 | 1.99E-03 | 8.89E-02 | 4.20E-01 |
| 848 | LOC113812953 | 6.38 | 6.26 | 14.36 | BGI_novel_T004217, XM_027364892.1, BGI_novel_T004218, BGI_novel_T004216 | 7.11E-01 | 7.05E-02 | 4.35E-02 |
| 849 | BGI_novel_G001577 | 0.45 | 0.00 | 0.21 | BGI_novel_T009511 | 3.74E-02 | 4.44E-01 | 1.57E-02 |
| 850 | LOC113822382 | 4.46 | 2.96 | 5.74 | BGI_novel_T006850, XM_027374915.1, XM_027374914.1, BGI_novel_T006848, BGI_novel_T006847, BGI_novel_T006849, BGI_novel_T006852, XM_027374916.1, BGI_novel_T006851 | 6.05E-03 | 7.07E-01 | 4.70E-02 |
| 851 | LOC113815725 | 0.60 | 0.10 | 0.94 | BGI_novel_T004997, XM_027367746.1 | 2.13E-02 | 9.61E-01 | 2.23E-02 |
| 852 | LOC113802828 | 0.04 | 0.00 | 0.12 | XM_027353468.1, XM_027353467.1 | 1.14E-02 | 6.74E-01 | 3.50E-02 |
| 853 | LOC113802111 | 14.53 | 12.20 | 16.84 | BGI_novel_T009795, XM_027352580.1, XM_027352584.1, BGI_novel_T009796, XM_027352582.1, XM_027352583.1, BGI_novel_T009794, BGI_novel_T009791, BGI_novel_T009792, XM_027352581.1, BGI_novel_T009793 | 2.82E-02 | 4.06E-01 | 3.10E-01 |
| 854 | LOC113800681 | 12.19 | 10.28 | 13.74 | BGI_novel_T009773, BGI_novel_T009771, BGI_novel_T009784, BGI_novel_T009786, BGI_novel_T009800, BGI_novel_T009768, BGI_novel_T009778, BGI_novel_T009781, BGI_novel_T009788, BGI_novel_T009775, BGI_novel_T009770, XM_027351485.1, BGI_novel_T009790, BGI_novel_T009783, BGI_novel_T009777, BGI_novel_T009780, BGI_novel_T009774, BGI_novel_T009772, BGI_novel_T009787, BGI_novel_T009769, BGI_novel_T009779, BGI_novel_T009782, BGI_novel_T009801, BGI_novel_T009785 | 2.95E-02 | 3.99E-01 | 4.58E-01 |
| 855 | LOC113810651 | 1.24 | 1.23 | 2.06 | XM_027362292.1 | 4.02E-01 | 1.47E-01 | 4.44E-02 |
| 856 | LOC113821310 | 14.32 | 10.68 | 11.86 | XM_027373802.1 | 2.48E-02 | 4.61E-03 | 7.12E-01 |
| 857 | LOC113815935 | 18.14 | 11.53 | 13.44 | XM_027367946.1 | 1.14E-03 | 1.27E-03 | 9.60E-01 |
| 858 | LOC113821008 | 5.86 | 5.04 | 6.04 | BGI_novel_T006493, XM_027373435.1, XM_027373434.1 | 1.13E-01 | 4.54E-02 | 7.73E-01 |
| 859 | LOC113807371 | 31.71 | 14.99 | 22.06 | XM_027358606.1 | 1.79E-04 | 1.78E-03 | 2.94E-01 |
| 860 | LOC113812616 | 77.04 | 73.57 | 108.39 | BGI_novel_T004163, BGI_novel_T004157, BGI_novel_T004153, BGI_novel_T004162, BGI_novel_T004152, XM_027364525.1, BGI_novel_T004164, BGI_novel_T004160, BGI_novel_T004154, BGI_novel_T004150, BGI_novel_T004156, BGI_novel_T004158, BGI_novel_T004159, BGI_novel_T004165, BGI_novel_T004155, BGI_novel_T004161, BGI_novel_T004151 | 1.32E-02 | 9.05E-01 | 5.33E-02 |
| 861 | LOC113825864 | 0.65 | 0.41 | 3.73 | BGI_novel_T007927, XM_027378699.1 | 6.37E-01 | 2.88E-02 | 4.14E-02 |
| 862 | LOC113827469 | 26.40 | 14.82 | 34.19 | XM_027380363.1, BGI_novel_T000888, BGI_novel_T000890, BGI_novel_T000891, BGI_novel_T000892, BGI_novel_T000887, BGI_novel_T000889 | 3.23E-02 | 6.92E-01 | 2.78E-02 |
| 863 | LOC113822032 | 0.02 | 0.01 | 0.15 | XM_027374544.1 | 4.67E-01 | 2.63E-02 | 5.81E-03 |
| 864 | LOC113806766 | 29.87 | 17.80 | 25.84 | XM_027357932.1 | 3.74E-02 | 1.59E-01 | 4.80E-01 |
| 865 | LOC113814281 | 15.40 | 10.90 | 17.65 | BGI_novel_T004622, BGI_novel_T004627, BGI_novel_T004625, BGI_novel_T004618, XM_027366322.1, BGI_novel_T004626, BGI_novel_T004619, BGI_novel_T004623, BGI_novel_T004620, BGI_novel_T004624, BGI_novel_T004621 | 1.05E-02 | 4.15E-01 | 8.51E-02 |
| 866 | LOC113809333 | 14.66 | 7.03 | 10.69 | XM_027360895.1, XM_027360898.1, XM_027360899.1, XM_027360897.1, XM_027360896.1 | 6.63E-03 | 7.54E-04 | 4.25E-01 |
| 867 | LOC113806937 | 12.14 | 9.08 | 10.28 | XM_027358061.1 | 6.91E-03 | 1.68E-01 | 9.53E-01 |
| 868 | LOC113810978 | 65.49 | 46.51 | 53.10 | XM_027362646.1 | 1.21E-01 | 2.81E-02 | 9.78E-01 |
| 869 | LOC113825529 | 2.52 | 0.20 | 1.11 | BGI_novel_T000077, XM_027378404.1 | 5.61E-08 | 3.45E-01 | 3.53E-02 |
| 870 | LOC113820710 | 19.35 | 10.32 | 25.65 | XM_027373053.1 | 2.39E-02 | 9.54E-01 | 6.37E-02 |
| 871 | LOC113813215 | 106.04 | 74.73 | 91.04 | BGI_novel_T004278, XM_027365174.1 | 3.76E-01 | 1.77E-02 | 4.28E-01 |
| 872 | LOC113821103 | 1.47 | 1.09 | 1.52 | XM_027373551.1 | 4.65E-02 | 6.47E-02 | 4.08E-01 |
| 873 | LOC113812736 | 2.70 | 1.85 | 2.29 | XM_027364661.1 | 1.62E-02 | 1.03E-02 | 9.55E-01 |
| 874 | LOC113810905 | 3.64 | 0.45 | 4.34 | XM_027362555.1, BGI_novel_T003660, XM_027362556.1 | 3.56E-03 | 7.36E-01 | 2.75E-02 |
| 875 | LOC113828101 | 0.71 | 0.01 | 0.68 | XM_027381040.1, XM_027381039.1 | 1.20E-03 | 6.44E-01 | 4.85E-02 |
| 876 | LOC113812565 | 0.53 | 0.32 | 0.68 | XM_027364472.1, BGI_novel_T004135 | 3.70E-02 | 7.41E-01 | 1.45E-01 |
| 877 | LOC113822453 | 1.00 | 0.87 | 2.84 | XM_027374997.1, XM_027374996.1 | 3.73E-01 | 1.88E-05 | 2.06E-03 |
| 878 | LOC113802475 | 0.35 | 0.14 | 0.67 | XM_027353076.1 | 1.80E-01 | 7.63E-01 | 3.81E-02 |
| 879 | LOC113815638 | 4.42 | 3.26 | 5.52 | BGI_novel_T004964, XM_027367662.1, BGI_novel_T004963 | 3.79E-02 | 5.03E-01 | 1.93E-01 |
| 880 | LOC113804180 | 0.44 | 0.02 | 0.77 | XM_027355018.1, BGI_novel_T000144 | 1.84E-02 | 8.60E-01 | 9.17E-02 |
| 881 | LOC113800357 | 5.02 | 4.19 | 5.79 | XM_027351120.1, BGI_novel_T009431, XM_027351121.1, XM_027351122.1 | 1.36E-02 | 2.28E-01 | 2.95E-01 |
| 882 | LOC113829323 | 0.17 | 0.13 | 0.41 | XM_027382471.1 | 5.25E-01 | 9.08E-02 | 2.92E-02 |
| 883 | LOC113821355 | 8.94 | 5.78 | 10.03 | XM_027373824.1, XM_027373826.1, BGI_novel_T006597, BGI_novel_T006600, BGI_novel_T006598, BGI_novel_T006604, XM_027373829.1, BGI_novel_T006601, XM_027373825.1, BGI_novel_T006596, BGI_novel_T006603, XM_027373828.1, BGI_novel_T006602, BGI_novel_T006599, XM_027373827.1 | 3.56E-03 | 3.87E-01 | 1.05E-01 |
| 884 | LOC113814185 | 2.81 | 2.01 | 2.49 | XM_027366207.1 | 1.85E-02 | 1.52E-01 | 7.65E-01 |
| 885 | BGI_novel_G001261 | 1.92 | 0.00 | 3.55 | BGI_novel_T007648 | 1.89E-01 | 9.40E-01 | 4.31E-02 |
| 886 | LOC113821079 | 7.70 | 7.53 | 7.71 | BGI_novel_T006503, BGI_novel_T006502, XM_027373519.1 | 8.44E-01 | 9.94E-03 | 1.88E-01 |
| 887 | LOC113812697 | 2.66 | 2.28 | 5.09 | BGI_novel_T004174, XM_027364618.1 | 1.61E-01 | 2.36E-01 | 8.44E-03 |
| 888 | LOC113818932 | 0.23 | 0.14 | 0.48 | XM_027371149.1 | 3.23E-01 | 3.93E-01 | 2.97E-02 |
| 889 | BGI_novel_G001445 | 84.33 | 72.61 | 77.65 | BGI_novel_T008777 | 1.49E-01 | 2.74E-02 | 6.19E-01 |
| 890 | BGI_novel_G001446 | 105.85 | 68.40 | 84.69 | BGI_novel_T008778 | 1.03E-03 | 1.04E-02 | 8.83E-01 |
| 891 | LOC113813205 | 0.27 | 0.14 | 0.32 | XM_027365165.1 | 1.67E-02 | 5.36E-01 | 8.23E-02 |
| 892 | LOC113829279 | 6.28 | 3.57 | 6.57 | BGI_novel_T008985, XM_027382403.1 | 4.10E-03 | 3.63E-01 | 5.06E-01 |
| 893 | LOC113822776 | 0.19 | 0.00 | 0.03 | XM_027375311.1 | 4.57E-02 | 1.25E-01 | 4.43E-01 |
| 894 | LOC113827072 | 1.03 | 1.01 | 3.43 | XM_027379966.1, BGI_novel_T008262, BGI_novel_T008263 | 4.58E-01 | 2.51E-02 | 1.44E-02 |
| 895 | LOC113810959 | 7.33 | 4.46 | 6.75 | BGI_novel_T003678, XM_027362620.1, BGI_novel_T003677, XM_027362621.1, BGI_novel_T003676, XM_027362619.1, XM_027362622.1, XM_027362625.1 | 3.63E-02 | 2.94E-02 | 3.75E-01 |
| 896 | LOC113819491 | 12.96 | 10.63 | 14.72 | XM_027371729.1, BGI_novel_T006046 | 2.29E-02 | 2.05E-01 | 2.04E-01 |
| 897 | LOC113819782 | 4.02 | 3.16 | 3.61 | XM_027371974.1, BGI_novel_T006123 | 4.22E-02 | 6.08E-04 | 8.61E-01 |
| 898 | LOC113804037 | 0.08 | 0.00 | 0.09 | XM_027354869.1 | 2.75E-02 | 8.78E-01 | 7.66E-02 |
| 899 | LOC113826633 | 1.88 | 0.10 | 1.42 | XM_027379524.1 | 3.00E-06 | 3.10E-01 | 1.93E-02 |
| 900 | LOC113830612 | 2.28 | 2.22 | 8.70 | BGI_novel_T009263, XM_027383834.1, BGI_novel_T009262 | 9.43E-01 | 1.39E-02 | 9.53E-02 |
| 901 | BGI_novel_G001519 | 73.57 | 55.66 | 71.00 | BGI_novel_T009268 | 1.08E-01 | 2.50E-03 | 9.49E-01 |
| 902 | LOC113812826 | 0.96 | 0.19 | 0.53 | XM_027364744.1 | 6.86E-04 | 7.37E-02 | 1.05E-01 |
| 903 | LOC113811553 | 224.85 | 36.83 | 70.09 | XM_027363323.1, BGI_novel_T003826 | 2.78E-08 | 1.39E-02 | 2.58E-01 |
| 904 | LOC113812805 | 0.69 | 0.17 | 0.46 | XM_027364721.1, BGI_novel_T004186 | 7.41E-09 | 3.40E-02 | 6.84E-02 |
| 905 | LOC113811544 | 219.50 | 117.72 | 177.18 | BGI_novel_T003823, BGI_novel_T003821, BGI_novel_T003819, BGI_novel_T003824, BGI_novel_T003820, XM_027363311.1, BGI_novel_T003822 | 2.69E-02 | 1.52E-01 | 5.10E-01 |
| 906 | LOC113806564 | 76.67 | 42.91 | 60.83 | BGI_novel_T002162, XM_027357721.1 | 3.02E-02 | 4.41E-02 | 6.17E-01 |
| 907 | LOC113809101 | 46.22 | 12.79 | 35.75 | XM_027360590.1 | 3.62E-02 | 5.01E-01 | 1.15E-01 |
| 908 | LOC113807141 | 1.97 | 1.39 | 2.44 | XM_027358347.1, BGI_novel_T002353, BGI_novel_T002354 | 2.87E-02 | 6.05E-01 | 2.68E-02 |
| 909 | LOC113826370 | 1.62 | 1.36 | 1.49 | XM_027379245.1 | 1.72E-01 | 1.45E-03 | 6.96E-01 |
| 910 | LOC113821474 | 3.28 | 2.23 | 4.04 | XM_027373983.1, XM_027373985.1, XM_027373984.1, XM_027373986.1, BGI_novel_T006637 | 6.01E-03 | 3.26E-01 | 3.17E-02 |
| 911 | LOC113812184 | 4.98 | 1.76 | 3.00 | XM_027364024.1 | 5.76E-05 | 9.09E-02 | 6.39E-01 |
| 912 | BGI_novel_G000619 | 1.03 | 0.27 | 0.81 | BGI_novel_T003750 | 3.81E-02 | 1.32E-02 | 2.15E-01 |
| 913 | LOC113805712 | 4.94 | 4.65 | 4.93 | XM_027356766.1 | 1.97E-01 | 6.10E-03 | 1.59E-01 |
| 914 | LOC113810329 | 5.08 | 4.44 | 5.25 | XM_027362009.1 | 2.49E-02 | 3.91E-02 | 9.43E-01 |
| 915 | LOC113807451 | 2.45 | 0.49 | 4.97 | XM_027358717.1 | 4.33E-02 | 4.75E-01 | 1.71E-04 |
| 916 | LOC113800833 | 5.51 | 4.16 | 5.77 | XM_027351628.1, XM_027351629.1, XM_027351630.1 | 3.37E-02 | 1.37E-01 | 3.19E-01 |
| 917 | LOC113815832 | 3.24 | 2.72 | 3.40 | XM_027367848.1, XM_027367847.1, XM_027367846.1 | 1.13E-01 | 3.57E-02 | 7.96E-01 |
| 918 | LOC113810792 | 0.46 | 0.38 | 1.04 | XM_027362435.1 | 5.66E-01 | 2.99E-02 | 1.12E-01 |
| 919 | LOC113820806 | 4.57 | 3.87 | 4.39 | XM_027373167.1 | 4.13E-02 | 8.94E-02 | 8.52E-01 |
| 920 | LOC113818752 | 2.32 | 0.80 | 2.25 | XM_027370943.1, XM_027370942.1 | 1.40E-02 | 4.75E-01 | 1.43E-01 |
| 921 | LOC113817907 | 37.83 | 23.34 | 47.54 | XM_027370029.1 | 8.09E-03 | 8.92E-01 | 8.53E-02 |
| 922 | LOC113813673 | 0.43 | 0.29 | 1.13 | XM_027365703.1, XM_027365702.1, XM_027365704.1 | 3.34E-01 | 1.64E-01 | 2.10E-02 |
| 923 | LOC113800432 | 2.05 | 0.07 | 1.39 | BGI_novel_T009448, XM_027351199.1 | 5.01E-07 | 3.39E-01 | 4.85E-02 |
| 924 | LOC113803654 | 3.03 | 2.30 | 3.32 | XM_027354460.1 | 4.50E-03 | 5.29E-02 | 2.54E-01 |
| 925 | LOC113826064 | 0.39 | 0.22 | 0.67 | XM_027378938.1 | 9.28E-02 | 5.09E-01 | 3.04E-02 |
| 926 | BGI_novel_G000140 | 11.36 | 7.20 | 8.01 | BGI_novel_T000998 | 2.80E-02 | 4.87E-03 | 8.16E-01 |
| 927 | LOC113805990 | 9.38 | 2.23 | 9.47 | XM_027357078.1 | 1.17E-03 | 5.56E-01 | 4.63E-02 |
| 928 | LOC113811177 | 0.31 | 0.23 | 1.48 | XM_027362858.1 | 8.49E-01 | 9.34E-03 | 2.91E-02 |
| 929 | LOC113811178 | 12.89 | 6.57 | 32.42 | XM_027362859.1 | 2.26E-01 | 1.23E-01 | 2.25E-02 |
| 930 | LOC113817364 | 4.08 | 1.90 | 3.72 | XM_027369399.1 | 6.45E-03 | 5.54E-02 | 2.15E-01 |
| 931 | LOC113812819 | 3.84 | 1.42 | 2.43 | XM_027364738.1, XM_027364737.1 | 5.65E-03 | 1.77E-02 | 2.77E-01 |
| 932 | LOC113828560 | 0.24 | 0.22 | 0.77 | XM_027381563.1 | 4.71E-01 | 1.54E-03 | 1.30E-03 |
| 933 | LOC113819364 | 3.95 | 2.36 | 5.46 | XM_027371598.1 | 3.89E-02 | 8.47E-01 | 3.76E-02 |
| 934 | LOC113816451 | 1.21 | 0.59 | 1.09 | XM_027368496.1, BGI_novel_T005241 | 2.27E-04 | 2.87E-02 | 6.87E-02 |
| 935 | LOC113823260 | 18.28 | 11.37 | 15.97 | XM_027375877.1, BGI_novel_T007133 | 6.17E-03 | 3.06E-02 | 3.19E-01 |
| 936 | LOC113806425 | 1.50 | 0.45 | 1.14 | XM_027357540.1, XM_027357539.1 | 3.04E-02 | 2.39E-01 | 1.46E-01 |
| 937 | LOC113805166 | 0.07 | 0.01 | 0.47 | XM_027356112.1 | 2.67E-01 | 2.07E-01 | 1.48E-02 |
| 938 | LOC113807511 | 0.93 | 0.83 | 3.80 | XM_027358781.1 | 4.84E-01 | 6.01E-02 | 2.36E-02 |
| 939 | LOC113810328 | 0.37 | 0.11 | 0.30 | XM_027362008.1, BGI_novel_T003435 | 8.90E-03 | 4.31E-01 | 2.57E-01 |
| 940 | LOC113815730 | 0.02 | 0.00 | 0.08 | XM_027367751.1 | 3.69E-01 | 1.83E-01 | 2.25E-02 |
| 941 | LOC113818991 | 2.82 | 2.05 | 2.38 | XM_027371229.1 | 7.10E-02 | 1.95E-04 | 6.97E-01 |
| 942 | LOC113819419 | 0.67 | 0.01 | 0.18 | XM_027371658.1 | 3.86E-03 | 1.37E-01 | 7.03E-02 |
| 943 | LOC113822746 | 1.46 | 0.04 | 0.44 | XM_027375286.1 | 1.07E-12 | 3.51E-04 | 1.36E-03 |
| 944 | LOC113829219 | 0.12 | 0.02 | 0.18 | XM_027382334.1 | 4.94E-02 | 9.61E-01 | 2.01E-02 |
| 945 | LOC113803831 | 0.55 | 0.51 | 1.85 | XM_027354646.1 | 7.01E-01 | 6.44E-03 | 4.49E-03 |
| 946 | BGI_novel_G001412 | 34.15 | 18.16 | 20.10 | BGI_novel_T008585 | 1.47E-04 | 3.03E-04 | 9.17E-01 |
| 947 | LOC113815356 | 11.56 | 9.58 | 22.75 | BGI_novel_T004909, XM_027367447.1 | 1.33E-01 | 2.91E-01 | 3.45E-02 |
| 948 | LOC113815890 | 7.71 | 2.60 | 3.74 | XM_027367906.1 | 4.03E-03 | 2.03E-02 | 5.91E-01 |
| 949 | LOC113820200 | 216.47 | 133.06 | 332.71 | XM_027372495.1, XM_027372493.1 | 1.61E-01 | 5.89E-01 | 4.98E-02 |
| 950 | LOC113824071 | 4.84 | 2.63 | 6.88 | XM_027376818.1, XM_027376819.1 | 3.92E-02 | 7.98E-01 | 3.69E-02 |
| 951 | LOC113808862 | 6.81 | 4.76 | 5.94 | XM_027360359.1, XM_027360358.1, BGI_novel_T002961 | 2.85E-01 | 2.09E-02 | 9.42E-01 |
| 952 | LOC113828523 | 51.75 | 30.43 | 47.84 | BGI_novel_T008686, XM_027381514.1, BGI_novel_T008684, XM_027381512.1, XM_027381510.1, BGI_novel_T008683, XM_027381513.1, BGI_novel_T008685, BGI_novel_T008688, XM_027381511.1, XM_027381509.1, BGI_novel_T008687 | 1.65E-02 | 1.49E-01 | 2.60E-01 |
| 953 | LOC113820623 | 1.65 | 1.56 | 3.43 | BGI_novel_T000605, BGI_novel_T000604, XM_027372966.1 | 6.86E-01 | 1.37E-01 | 3.79E-02 |
| 954 | LOC113824781 | 36.26 | 24.93 | 31.06 | XM_027377569.1, BGI_novel_T007650 | 4.03E-03 | 4.51E-03 | 9.89E-01 |
| 955 | LOC113808485 | 2.09 | 1.33 | 1.63 | XM_027359903.1 | 5.61E-03 | 2.46E-02 | 9.45E-01 |
| 956 | LOC113807665 | 8.56 | 1.87 | 3.76 | XM_027358976.1, XM_027358978.1, XM_027358979.1, XM_027358977.1 | 4.79E-04 | 2.20E-02 | 2.75E-01 |
| 957 | LOC113804364 | 0.18 | 0.08 | 0.38 | XM_027355220.1, BGI_novel_T001427 | 1.18E-02 | 7.99E-01 | 1.39E-02 |
| 958 | BGI_novel_G000494 | 4.08 | 1.90 | 3.60 | BGI_novel_T003036 | 4.18E-03 | 1.08E-01 | 2.17E-01 |
| 959 | LOC113805316 | 0.86 | 0.36 | 0.94 | XM_027356310.1 | 2.22E-02 | 6.34E-01 | 3.34E-01 |
| 960 | LOC113803453 | 0.87 | 0.78 | 3.08 | XM_027354238.1 | 6.05E-01 | 1.52E-01 | 3.58E-02 |
| 961 | LOC113804667 | 0.39 | 0.10 | 0.70 | BGI_novel_T001516, XM_027355549.1 | 1.05E-01 | 8.49E-01 | 3.16E-02 |
| 962 | LOC113828398 | 73.73 | 45.74 | 63.77 | XM_027381364.1 | 2.71E-04 | 1.62E-02 | 4.24E-01 |
| 963 | LOC113824401 | 4.15 | 2.22 | 8.35 | XM_027377141.1 | 2.92E-01 | 5.34E-01 | 4.11E-02 |
| 964 | LOC113823422 | 2.05 | 0.90 | 1.19 | XM_027376048.1, BGI_novel_T007170 | 2.04E-03 | 8.52E-03 | 6.57E-01 |
| 965 | LOC113813399 | 0.75 | 0.57 | 1.36 | XM_027365369.1 | 2.88E-01 | 3.03E-01 | 3.47E-02 |
| 966 | LOC113802342 | 0.18 | 0.04 | 0.17 | XM_027352901.1 | 2.86E-02 | 6.18E-01 | 2.70E-01 |
| 967 | LOC113817363 | 1.50 | 0.64 | 0.92 | XM_027369398.1 | 6.39E-02 | 4.60E-03 | 6.56E-01 |
| 968 | LOC113815352 | 0.20 | 0.00 | 0.22 | XM_027367444.1 | 1.18E-03 | 7.28E-01 | 7.30E-02 |
| 969 | LOC113808140 | 0.06 | 0.01 | 0.30 | XM_027359475.1 | 3.52E-01 | 1.17E-01 | 2.78E-03 |
| 970 | LOC113802072 | 2.29 | 2.02 | 6.48 | XM_027352536.1 | 3.21E-01 | 9.43E-02 | 2.65E-02 |
| 971 | LOC113807941 | 0.01 | 0.00 | 0.04 | XM_027359267.1 | 2.96E-01 | 3.86E-01 | 4.55E-02 |
| 972 | LOC113814221 | 1.23 | 1.06 | 2.29 | XM_027366257.1, XM_027366256.1 | 3.69E-01 | 4.04E-01 | 9.56E-03 |
| 973 | LOC113821731 | 0.24 | 0.11 | 0.52 | XM_027374257.1 | 4.99E-02 | 5.43E-01 | 5.75E-02 |
| 974 | BGI_novel_G000195 | 15.28 | 0.00 | 6.20 | BGI_novel_T001289 | 4.51E-03 | 5.04E-01 | 2.72E-01 |
| 975 | LOC113819745 | 2.96 | 2.66 | 13.10 | XM_027371937.1 | 5.82E-01 | 4.40E-02 | 4.36E-02 |
| 976 | LOC113808863 | 5.17 | 2.73 | 4.10 | BGI_novel_T002962, BGI_novel_T002963, XM_027360361.1, BGI_novel_T002964 | 1.26E-02 | 2.38E-01 | 4.03E-01 |
| 977 | LOC113800831 | 177.08 | 108.20 | 140.49 | XM_027351627.1, BGI_novel_T009558 | 1.42E-05 | 2.21E-02 | 5.64E-01 |
| 978 | LOC113813261 | 0.17 | 0.03 | 0.14 | XM_027365226.1, XM_027365225.1 | 2.00E-03 | 3.87E-01 | 1.42E-02 |
| 979 | LOC113815350 | 0.12 | 0.01 | 0.24 | BGI_novel_T004905, XM_027367442.1 | 4.53E-03 | 9.35E-01 | 3.96E-02 |
| 980 | LOC113809637 | 204.01 | 31.27 | 114.56 | XM_027361286.1, XM_027361288.1, XM_027361287.1, XM_027361285.1 | 5.21E-05 | 3.48E-02 | 3.01E-02 |
| 981 | LOC113811068 | 0.10 | 0.03 | 0.07 | XM_027362738.1 | 4.66E-02 | 2.21E-01 | 4.91E-01 |
| 982 | LOC113830142 | 0.16 | 0.01 | 0.42 | XM_027383346.1, XM_027383345.1 | 1.31E-02 | 3.44E-01 | 2.81E-03 |
| 983 | LOC113806454 | 805.47 | 198.85 | 262.91 | BGI_novel_T002128, BGI_novel_T002131, BGI_novel_T002130, BGI_novel_T002127, XM_027357587.1, BGI_novel_T002132, BGI_novel_T002134, BGI_novel_T002133, BGI_novel_T002129 | 7.70E-04 | 3.53E-04 | 7.62E-01 |
| 984 | LOC113814381 | 5.12 | 2.44 | 3.67 | XM_027366431.1, XM_027366438.1, BGI_novel_T004662, XM_027366433.1, XM_027366432.1, XM_027366436.1, XM_027366434.1, XM_027366435.1, XM_027366430.1, XM_027366437.1 | 4.91E-02 | 9.52E-02 | 4.56E-01 |
| 985 | LOC113800862 | 0.30 | 0.11 | 0.25 | XM_027351666.1, XM_027351661.1, XM_027351667.1, XM_027351664.1, XM_027351663.1, XM_027351662.1 | 3.17E-02 | 2.63E-01 | 1.94E-01 |
| 986 | LOC113817231 | 1.46 | 0.31 | 2.39 | XM_027369251.1, BGI_novel_T005416 | 2.88E-03 | 9.97E-01 | 8.10E-04 |
| 987 | LOC113809890 | 0.02 | 0.00 | 0.02 | XM_027361565.1 | 4.40E-02 | 8.48E-01 | 2.43E-01 |
| 988 | LOC113806268 | 59.38 | 41.73 | 53.21 | XM_027357384.1 | 3.99E-04 | 1.50E-03 | 8.23E-01 |
| 989 | LOC113810980 | 233.82 | 198.42 | 221.42 | XM_027362648.1, XM_027362649.1 | 4.41E-02 | 1.24E-01 | 8.29E-01 |
| 990 | LOC113801042 | 0.56 | 0.37 | 0.78 | XM_027351848.1, XM_027351849.1 | 4.22E-02 | 9.19E-01 | 9.59E-02 |
| 991 | BGI_novel_G000285 | 22.25 | 12.21 | 17.04 | BGI_novel_T001719 | 1.29E-02 | 9.08E-03 | 5.56E-01 |
| 992 | LOC113827214 | 0.30 | 0.02 | 0.44 | XM_027380110.1 | 4.87E-02 | 8.99E-01 | 7.55E-02 |
| 993 | LOC113804193 | 35.00 | 22.69 | 32.50 | BGI_novel_T001388, BGI_novel_T001389, BGI_novel_T001391, XM_027355025.1, BGI_novel_T001390 | 4.57E-02 | 1.25E-01 | 6.21E-01 |
| 994 | LOC113806696 | 13.41 | 9.83 | 13.14 | XM_027357865.1, XM_027357861.1, XM_027357864.1, XM_027357862.1, XM_027357863.1 | 1.31E-02 | 2.60E-03 | 5.41E-01 |
| 995 | LOC113827766 | 0.43 | 0.17 | 0.31 | XM_027380663.1 | 4.93E-02 | 1.54E-01 | 4.54E-01 |
| 996 | LOC113818940 | 1.55 | 1.06 | 1.27 | XM_027371157.1 | 3.54E-01 | 2.03E-02 | 9.45E-01 |
| 997 | LOC113829244 | 0.01 | 0.01 | 0.16 | XM_027382364.1 | 5.24E-01 | 5.79E-02 | 1.15E-02 |
| 998 | LOC113804475 | 24.54 | 13.59 | 14.52 | BGI_novel_T001461, XM_027355361.1, BGI_novel_T001462, BGI_novel_T001460 | 4.61E-04 | 1.59E-04 | 7.92E-01 |
| 999 | LOC113817184 | 11.42 | 8.42 | 13.25 | XM_027369196.1, BGI_novel_T005389 | 1.56E-02 | 3.00E-01 | 1.56E-01 |
| 1000 | LOC113800802 | 5.24 | 3.53 | 6.80 | BGI_novel_T009547, XM_027351588.1, BGI_novel_T009549, BGI_novel_T009548, BGI_novel_T009550 | 8.60E-03 | 8.83E-01 | 7.49E-02 |
| 1001 | LOC113815384 | 1.10 | 0.02 | 1.77 | XM_027367472.1, BGI_novel_T004917 | 6.54E-04 | 9.22E-01 | 2.38E-02 |
| 1002 | LOC113822204 | 0.62 | 0.12 | 1.14 | XM_027374743.1 | 1.90E-02 | 9.48E-01 | 2.43E-02 |
| 1003 | LOC113803637 | 18.61 | 11.71 | 14.65 | XM_027354444.1 | 3.55E-03 | 1.07E-01 | 6.89E-01 |
| 1004 | LOC113823053 | 16.20 | 8.88 | 10.69 | XM_027375658.1, BGI_novel_T007052, BGI_novel_T007053, XM_027375657.1, XM_027375659.1 | 9.30E-04 | 1.62E-03 | 9.78E-01 |
| 1005 | LOC113817572 | 17.32 | 10.54 | 13.73 | XM_027369619.1 | 2.44E-03 | 2.94E-02 | 9.77E-01 |
| 1006 | LOC113823180 | 30.80 | 19.27 | 21.87 | XM_027375797.1 | 7.65E-04 | 1.75E-04 | 6.53E-01 |
| 1007 | LOC113802172 | 5.00 | 2.48 | 21.38 | XM_027352694.1, BGI_novel_T009849, BGI_novel_T009850, XM_027352693.1, BGI_novel_T009848 | 1.37E-02 | 1.34E-01 | 6.82E-03 |
| 1008 | LOC113817744 | 0.54 | 0.14 | 0.67 | XM_027369829.1 | 3.16E-02 | 9.30E-01 | 3.35E-02 |
| 1009 | LOC113817412 | 0.82 | 0.80 | 2.03 | XM_027369475.1, XM_027369472.1, XM_027369469.1, XM_027369470.1, XM_027369476.1, XM_027369473.1, XM_027369474.1 | 8.65E-01 | 4.00E-02 | 2.45E-01 |
| 1010 | LOC113817008 | 41.69 | 25.54 | 33.72 | XM_027369005.1 | 3.11E-03 | 7.80E-03 | 5.89E-01 |
| 1011 | LOC113812359 | 0.31 | 0.07 | 0.27 | XM_027364233.1 | 3.57E-02 | 4.35E-01 | 9.95E-02 |
| 1012 | LOC113803909 | 3.16 | 1.87 | 3.23 | XM_027354719.1, BGI_novel_T003773 | 1.85E-03 | 1.93E-01 | 7.78E-02 |
| 1013 | LOC113809297 | 0.34 | 0.05 | 0.22 | XM_027360834.1 | 3.23E-02 | 2.34E-01 | 3.75E-01 |
| 1014 | LOC113807825 | 0.81 | 0.81 | 2.38 | XM_027359110.1 | 8.31E-01 | 1.95E-01 | 4.58E-02 |
| 1015 | LOC113805952 | 2.99 | 2.62 | 4.06 | XM_027357036.1 | 3.05E-02 | 7.78E-01 | 1.66E-01 |
| 1016 | LOC113805384 | 0.31 | 0.06 | 0.20 | XM_027356374.1 | 7.41E-03 | 4.31E-01 | 9.55E-02 |
| 1017 | LOC113817117 | 3.79 | 2.44 | 4.41 | XM_027369124.1 | 7.21E-03 | 3.76E-01 | 8.82E-02 |
| 1018 | LOC113802855 | 0.26 | 0.11 | 0.44 | XM_027353504.1, XM_027353505.1 | 4.34E-02 | 9.46E-01 | 7.61E-02 |
| 1019 | LOC113802754 | 1.43 | 0.18 | 0.95 | XM_027353367.1 | 1.25E-04 | 1.49E-01 | 8.24E-02 |
| 1020 | LOC113822056 | 37.01 | 8.98 | 16.75 | XM_027374574.1 | 2.51E-04 | 9.11E-04 | 2.17E-01 |
| 1021 | LOC113807540 | 0.23 | 0.00 | 0.09 | XM_027358821.1 | 2.92E-04 | 7.06E-02 | 6.97E-02 |
| 1022 | LOC113823521 | 0.07 | 0.00 | 0.05 | XM_027376185.1 | 4.46E-02 | 4.08E-01 | 1.55E-01 |
| 1023 | LOC113815106 | 0.28 | 0.20 | 1.07 | XM_027367187.1 | 5.62E-01 | 1.23E-01 | 1.44E-02 |
| 1024 | LOC113820254 | 0.86 | 0.15 | 1.06 | XM_027372570.1 | 4.47E-03 | 8.27E-01 | 2.38E-02 |
| 1025 | LOC113804488 | 0.72 | 0.11 | 0.88 | XM_027355374.1, BGI_novel_T001465 | 1.32E-02 | 7.28E-01 | 8.74E-02 |
| 1026 | LOC113803966 | 1.35 | 0.31 | 0.63 | XM_027354773.1 | 4.94E-03 | 6.10E-02 | 4.36E-01 |
| 1027 | LOC113829620 | 1.13 | 0.86 | 1.01 | XM_027382823.1 | 4.98E-02 | 1.69E-02 | 9.09E-01 |
| 1028 | LOC113815447 | 1.51 | 0.55 | 13.43 | BGI_novel_T004919, XM_027367515.1 | 4.36E-01 | 3.31E-01 | 4.27E-02 |
| 1029 | LOC113808334 | 0.09 | 0.06 | 0.30 | XM_027359741.1 | 5.44E-01 | 2.08E-01 | 4.51E-02 |
| 1030 | LOC113826373 | 0.43 | 0.00 | 0.17 | XM_027379248.1 | 3.69E-03 | 1.49E-01 | 1.73E-01 |
| 1031 | LOC113814380 | 0.45 | 0.40 | 0.90 | XM_027366429.1 | 4.54E-01 | 2.11E-01 | 3.20E-02 |
| 1032 | LOC113814529 | 0.17 | 0.02 | 0.48 | XM_027366571.1, XM_027366572.1 | 3.43E-01 | 6.30E-01 | 1.27E-02 |
| 1033 | LOC113799941 | 0.79 | 0.36 | 0.52 | XM_027350643.1, XM_027350642.1 | 3.57E-02 | 7.71E-02 | 6.50E-01 |
| 1034 | LOC113800043 | 0.26 | 0.02 | 0.79 | XM_027350766.1 | 1.82E-01 | 5.29E-01 | 2.55E-02 |
| 1035 | LOC113800821 | 0.05 | 0.01 | 0.19 | XM_027351618.1 | 1.39E-01 | 2.25E-01 | 5.05E-03 |
| 1036 | LOC113800854 | 0.43 | 0.25 | 0.73 | XM_027351651.1 | 2.11E-01 | 6.24E-01 | 4.92E-02 |
| 1037 | LOC113800980 | 6.18 | 3.51 | 7.01 | XM_027351791.1 | 1.78E-02 | 7.76E-01 | 1.27E-01 |
| 1038 | LOC113801102 | 1.35 | 0.44 | 0.86 | BGI_novel_T001006, XM_027351921.1, XM_027351945.1, XM_027351925.1, XM_027351932.1, XM_027351938.1 | 5.94E-03 | 5.48E-03 | 2.58E-01 |
| 1039 | LOC113801235 | 13.02 | 6.73 | 9.01 | XM_027352054.1, XM_027352053.1, BGI_novel_T009654, XM_027352045.1, BGI_novel_T009651, XM_027352052.1, XM_027352050.1, XM_027352047.1, BGI_novel_T009653, XM_027352048.1, XM_027352051.1, BGI_novel_T009652, XM_027352049.1 | 3.55E-02 | 1.83E-02 | 6.92E-01 |
| 1040 | LOC113802069 | 0.81 | 0.00 | 7.03 | XM_027352528.1 | 5.38E-03 | 1.73E-01 | 8.10E-03 |
| 1041 | LOC113802176 | 81.01 | 53.51 | 76.01 | XM_027352700.1 | 1.59E-02 | 2.13E-01 | 4.29E-01 |
| 1042 | LOC113802474 | 8.20 | 7.07 | 10.78 | XM_027353094.1, BGI_novel_T001022, XM_027353079.1, XM_027353086.1 | 3.01E-02 | 8.89E-01 | 1.20E-01 |
| 1043 | LOC113802506 | 0.50 | 0.00 | 0.13 | XM_027353110.1 | 1.72E-02 | 3.05E-01 | 3.70E-01 |
| 1044 | LOC113802821 | 0.07 | 0.02 | 0.25 | XM_027353449.1 | 6.38E-02 | 2.32E-01 | 8.78E-03 |
| 1045 | LOC113802896 | 7.87 | 6.57 | 8.72 | BGI_novel_T000105, XM_027353548.1, XM_027353561.1, XM_027353554.1 | 4.83E-02 | 4.42E-02 | 6.61E-01 |
| 1046 | LOC113803672 | 3.08 | 1.89 | 3.09 | XM_027354477.1 | 2.54E-02 | 1.72E-01 | 6.80E-02 |
| 1047 | LOC113803696 | 12.02 | 11.51 | 57.41 | XM_027354507.1 | 9.83E-01 | 1.11E-01 | 4.76E-02 |
| 1048 | LOC113803974 | 388.80 | 144.72 | 239.08 | XM_027354784.1 | 1.92E-02 | 6.04E-02 | 4.86E-01 |
| 1049 | LOC113804000 | 16.65 | 11.22 | 12.61 | XM_027354817.1 | 1.45E-01 | 1.00E-02 | 9.14E-01 |
| 1050 | LOC113804029 | 8.28 | 4.41 | 4.67 | XM_027354857.1 | 5.60E-04 | 1.80E-04 | 7.47E-01 |
| 1051 | LOC113804412 | 7.21 | 4.67 | 11.20 | BGI_novel_T001444, XM_027355284.1, BGI_novel_T001443, XM_027355282.1 | 3.32E-02 | 7.45E-01 | 8.56E-02 |
| 1052 | LOC113804491 | 29.90 | 17.87 | 28.97 | BGI_novel_T000149, XM_027355381.1, BGI_novel_T000148 | 4.13E-02 | 1.91E-01 | 3.98E-01 |
| 1053 | LOC113804631 | 78.29 | 34.73 | 56.46 | XM_027355520.1 | 4.92E-03 | 1.21E-01 | 3.18E-01 |
| 1054 | LOC113804790 | 10.13 | 2.66 | 9.58 | XM_027355689.1 | 6.37E-06 | 2.78E-01 | 2.30E-02 |
| 1055 | LOC113805617 | 0.84 | 0.42 | 0.82 | BGI_novel_T001837, XM_027356660.1 | 1.11E-02 | 4.29E-01 | 1.25E-01 |
| 1056 | LOC113805638 | 11.69 | 5.97 | 10.23 | XM_027356691.1, XM_027356690.1, XM_027356689.1 | 3.37E-02 | 4.51E-02 | 3.03E-01 |
| 1057 | LOC113805645 | 2.99 | 2.01 | 2.45 | XM_027356700.1 | 7.58E-02 | 3.87E-02 | 9.53E-01 |
| 1058 | LOC113805934 | 0.09 | 0.00 | 4.82 | XM_027357013.1 | 4.35E-01 | 1.75E-02 | 2.11E-03 |
| 1059 | LOC113806191 | 3.53 | 0.52 | 4.58 | BGI_novel_T000189, BGI_novel_T000190, XM_027357316.1, BGI_novel_T000191 | 3.67E-02 | 6.45E-01 | 9.87E-02 |
| 1060 | LOC113806392 | 3.74 | 2.95 | 3.10 | XM_027357519.1 | 1.93E-01 | 4.77E-02 | 5.52E-01 |
| 1061 | LOC113806565 | 4.55 | 2.21 | 4.67 | XM_027357723.1 | 4.82E-03 | 4.52E-01 | 6.87E-02 |
| 1062 | LOC113807369 | 0.64 | 0.26 | 1.38 | XM_027358603.1, XM_027358602.1 | 6.19E-02 | 2.79E-01 | 2.17E-03 |
| 1063 | LOC113807424 | 30.94 | 25.57 | 29.35 | XM_027358676.1 | 1.04E-01 | 4.33E-02 | 7.99E-01 |
| 1064 | LOC113807624 | 40.11 | 26.49 | 30.33 | XM_027358933.1 | 9.66E-03 | 8.82E-03 | 9.08E-01 |
| 1065 | LOC113807686 | 0.72 | 0.03 | 1.67 | XM_027359003.1 | 5.39E-03 | 8.86E-01 | 2.98E-02 |
| 1066 | LOC113807807 | 18.67 | 5.16 | 6.83 | XM_027359092.1, BGI_novel_T002651, BGI_novel_T002652 | 3.76E-02 | 2.63E-01 | 9.95E-01 |
| 1067 | LOC113808400 | 2.63 | 0.41 | 0.84 | XM_027359811.1 | 1.22E-01 | 2.73E-02 | 7.36E-01 |
| 1068 | LOC113808473 | 4.42 | 2.35 | 4.80 | XM_027359887.1, XM_027359889.1, XM_027359892.1, BGI_novel_T002808, XM_027359893.1, XM_027359890.1, BGI_novel_T002810, BGI_novel_T002809, XM_027359888.1 | 3.93E-02 | 1.02E-01 | 1.98E-01 |
| 1069 | LOC113808509 | 0.30 | 0.05 | 0.51 | XM_027359936.1 | 1.82E-01 | 6.34E-01 | 4.68E-02 |
| 1070 | LOC113808822 | 4.44 | 2.08 | 8.75 | BGI_novel_T002942, XM_027360321.1 | 3.50E-03 | 6.55E-01 | 8.02E-02 |
| 1071 | LOC113808996 | 2.10 | 0.52 | 3.52 | BGI_novel_T003001, BGI_novel_T003000, XM_027360477.1, XM_027360478.1 | 6.38E-03 | 9.53E-01 | 1.48E-01 |
| 1072 | LOC113809538 | 798.78 | 186.24 | 341.12 | BGI_novel_T003218, BGI_novel_T003217, BGI_novel_T003219, BGI_novel_T003216, XM_027361177.1, BGI_novel_T003220, BGI_novel_T003214, BGI_novel_T003213, BGI_novel_T003221, XM_027361178.1, BGI_novel_T003211, BGI_novel_T003215, BGI_novel_T003212 | 1.10E-02 | 5.83E-03 | 7.04E-01 |
| 1073 | LOC113809894 | 0.21 | 0.06 | 0.44 | XM_027361568.1 | 3.54E-02 | 5.88E-01 | 7.09E-03 |
| 1074 | LOC113810078 | 0.43 | 0.13 | 0.30 | XM_027361763.1 | 1.69E-04 | 3.28E-02 | 1.91E-01 |
| 1075 | LOC113804490 | 0.20 | 0.00 | 0.17 | XM_027355376.1 | 4.72E-02 | 7.60E-01 | 2.01E-01 |
| 1076 | LOC113811180 | 1.47 | 0.95 | 1.78 | XM_027362860.1 | 4.22E-02 | 6.46E-01 | 1.21E-01 |
| 1077 | LOC113811226 | 61.27 | 37.15 | 51.62 | XM_027362906.1, BGI_novel_T003735, BGI_novel_T003734 | 1.90E-02 | 6.94E-02 | 5.21E-01 |
| 1078 | LOC113811295 | 4.47 | 0.45 | 2.91 | XM_027363009.1, BGI_novel_T003745 | 2.09E-03 | 2.31E-01 | 1.52E-03 |
| 1079 | LOC113812011 | 29.95 | 14.24 | 20.28 | XM_027363875.1 | 7.20E-04 | 3.44E-04 | 4.59E-01 |
| 1080 | LOC113812014 | 15.22 | 9.92 | 13.65 | XM_027363879.1 | 1.24E-02 | 2.94E-02 | 4.60E-01 |
| 1081 | LOC113812193 | 5.25 | 1.71 | 3.39 | XM_027364033.1 | 1.26E-03 | 2.59E-02 | 3.77E-01 |
| 1082 | LOC113812481 | 9.93 | 2.45 | 5.57 | XM_027364383.1 | 3.12E-03 | 3.78E-02 | 1.39E-01 |
| 1083 | LOC113813552 | 32.43 | 18.86 | 39.09 | XM_027365556.1 | 1.21E-02 | 5.30E-01 | 1.25E-01 |
| 1084 | LOC113813755 | 0.06 | 0.01 | 0.02 | XM_027365814.1 | 3.71E-03 | 1.64E-03 | 7.47E-01 |
| 1085 | LOC113815370 | 4.32 | 0.13 | 5.38 | XM_027367460.1 | 2.34E-03 | 8.07E-01 | 1.53E-02 |
| 1086 | LOC113815912 | 0.33 | 0.07 | 0.60 | XM_027367923.1 | 2.36E-01 | 6.75E-01 | 1.33E-02 |
| 1087 | LOC113816122 | 1.74 | 0.57 | 1.78 | XM_027368142.1, XM_027368143.1, XM_027368140.1, XM_027368139.1, XM_027368141.1 | 9.22E-04 | 2.74E-01 | 1.80E-02 |
| 1088 | LOC113816183 | 1.55 | 1.16 | 2.83 | XM_027368228.1 | 2.65E-01 | 2.68E-01 | 1.89E-02 |
| 1089 | LOC113816198 | 1.11 | 0.24 | 0.31 | XM_027368245.1, BGI_novel_T000478 | 7.61E-03 | 9.72E-03 | 8.95E-01 |
| 1090 | LOC113816321 | 8.08 | 5.66 | 9.76 | BGI_novel_T005195, BGI_novel_T005198, XM_027368364.1, BGI_novel_T005196, BGI_novel_T005197 | 3.34E-02 | 3.11E-01 | 1.37E-01 |
| 1091 | LOC113816641 | 0.39 | 0.24 | 0.73 | XM_027368698.1, XM_027368697.1, XM_027368699.1, BGI_novel_T005295, XM_027368696.1, XM_027368701.1 | 7.46E-02 | 3.97E-01 | 7.34E-03 |
| 1092 | LOC113816671 | 0.80 | 0.20 | 2.05 | XM_027368729.1 | 1.64E-01 | 5.72E-01 | 1.24E-02 |
| 1093 | LOC113816758 | 0.39 | 0.06 | 1.37 | XM_027368819.1 | 1.49E-01 | 3.81E-01 | 4.04E-02 |
| 1094 | LOC113817001 | 1.33 | 0.55 | 1.70 | BGI_novel_T005340, XM_027368998.1 | 1.71E-02 | 9.61E-01 | 3.61E-02 |
| 1095 | LOC113817274 | 68.94 | 27.03 | 49.45 | XM_027369299.1 | 4.18E-03 | 4.32E-02 | 1.57E-01 |
| 1096 | LOC113817276 | 28.02 | 19.01 | 23.56 | XM_027369301.1, XM_027369300.1 | 3.93E-02 | 1.60E-01 | 6.95E-01 |
| 1097 | LOC113817662 | 6.02 | 3.72 | 6.06 | BGI_novel_T005544, BGI_novel_T005545, XM_027369732.1 | 3.42E-02 | 3.57E-01 | 3.32E-01 |
| 1098 | LOC113817946 | 7.35 | 2.89 | 3.43 | XM_027370069.1, BGI_novel_T000554 | 6.42E-05 | 4.74E-05 | 8.67E-01 |
| 1099 | LOC113818213 | 0.15 | 0.05 | 0.16 | XM_027370390.1 | 1.22E-02 | 6.15E-01 | 4.43E-02 |
| 1100 | LOC113818687 | 8.00 | 5.48 | 7.00 | XM_027370875.1 | 2.76E-03 | 1.18E-02 | 9.19E-01 |
| 1101 | LOC113818762 | 0.23 | 0.00 | 0.21 | XM_027370949.1 | 4.88E-02 | 6.91E-01 | 4.17E-01 |
| 1102 | LOC113818885 | 0.99 | 0.44 | 1.06 | XM_027371097.1, XM_027371096.1, BGI_novel_T005871 | 2.52E-02 | 5.54E-01 | 4.30E-01 |
| 1103 | LOC113819222 | 761.03 | 255.38 | 861.03 | BGI_novel_T005984, BGI_novel_T005983, XM_027371472.1 | 1.42E-02 | 8.35E-01 | 2.70E-02 |
| 1104 | LOC113819228 | 2.39 | 0.51 | 3.23 | XM_027371478.1 | 6.00E-02 | 8.61E-01 | 8.69E-03 |
| 1105 | LOC113819394 | 4.85 | 0.91 | 4.60 | XM_027371634.1, XM_027371633.1, BGI_novel_T006021, BGI_novel_T006022, XM_027371635.1 | 3.52E-05 | 3.47E-01 | 9.46E-03 |
| 1106 | LOC113819403 | 0.58 | 0.06 | 6.38 | XM_027371645.1 | 3.02E-01 | 1.62E-01 | 3.53E-02 |
| 1107 | LOC113819619 | 0.08 | 0.01 | 0.11 | XM_027371826.1 | 3.67E-02 | 8.52E-01 | 6.40E-02 |
| 1108 | LOC113819742 | 0.15 | 0.01 | 0.07 | XM_027371935.1 | 8.31E-05 | 3.67E-02 | 2.19E-01 |
| 1109 | LOC113819781 | 9.39 | 8.72 | 9.64 | XM_027372022.1, XM_027372050.1, XM_027372034.1, XM_027372005.1, XM_027371975.1, XM_027371981.1, BGI_novel_T000585, XM_027372015.1, BGI_novel_T000586, XM_027372010.1, XM_027371999.1, BGI_novel_T000587, XM_027371991.1 | 1.21E-01 | 2.96E-02 | 5.66E-01 |
| 1110 | LOC113819839 | 1.58 | 0.73 | 1.82 | XM_027372046.1, XM_027372045.1 | 2.18E-03 | 8.61E-01 | 6.33E-02 |
| 1111 | LOC113820080 | 0.44 | 0.17 | 0.78 | XM_027372338.1 | 8.39E-02 | 5.29E-01 | 5.51E-03 |
| 1112 | LOC113820105 | 1.42 | 0.40 | 0.84 | BGI_novel_T006221, BGI_novel_T006222, XM_027372371.1 | 2.11E-02 | 4.51E-01 | 2.84E-01 |
| 1113 | LOC113820187 | 0.07 | 0.02 | 0.10 | XM_027372469.1 | 1.35E-01 | 9.83E-01 | 4.73E-02 |
| 1114 | LOC113820206 | 3.91 | 3.67 | 12.62 | XM_027372507.1, BGI_novel_T006243 | 8.93E-01 | 3.24E-02 | 8.73E-02 |
| 1115 | LOC113820349 | 4.73 | 0.26 | 4.79 | XM_027372685.1, XM_027372684.1 | 8.84E-03 | 7.28E-01 | 4.89E-02 |
| 1116 | LOC113820869 | 61.78 | 40.78 | 92.31 | BGI_novel_T006418, BGI_novel_T006419, XM_027373254.1 | 2.55E-02 | 6.61E-01 | 8.89E-03 |
| 1117 | LOC113821400 | 69.89 | 6.86 | 20.64 | XM_027373884.1 | 1.45E-05 | 4.07E-04 | 1.16E-01 |
| 1118 | LOC113822071 | 28.40 | 16.87 | 21.61 | XM_027374594.1 | 3.22E-02 | 2.85E-02 | 8.95E-01 |
| 1119 | LOC113822408 | 6.93 | 3.62 | 4.39 | XM_027374942.1, BGI_novel_T006862, BGI_novel_T006861 | 4.02E-02 | 3.27E-05 | 9.51E-01 |
| 1120 | LOC113822794 | 0.22 | 0.05 | 0.34 | XM_027375327.1 | 2.45E-02 | 9.08E-01 | 1.53E-01 |
| 1121 | LOC113822982 | 0.67 | 0.37 | 1.87 | XM_027375535.1 | 4.26E-01 | 4.37E-01 | 4.74E-02 |
| 1122 | LOC113823614 | 0.10 | 0.01 | 0.22 | XM_027376303.1 | 3.16E-01 | 6.34E-01 | 5.03E-03 |
| 1123 | LOC113823803 | 0.11 | 0.03 | 0.26 | XM_027376490.1 | 1.92E-01 | 5.66E-01 | 4.84E-02 |
| 1124 | LOC113823850 | 19.22 | 5.67 | 9.33 | XM_027376559.1, BGI_novel_T007299, BGI_novel_T007298, BGI_novel_T007301, BGI_novel_T007302, BGI_novel_T007300 | 5.15E-02 | 3.50E-02 | 5.07E-01 |
| 1125 | LOC113823851 | 19.18 | 4.14 | 14.41 | XM_027376560.1, XM_027376561.1 | 9.51E-07 | 3.40E-02 | 8.34E-05 |
| 1126 | LOC113823862 | 4.80 | 0.95 | 1.38 | BGI_novel_T007307, XM_027376576.1, BGI_novel_T007305, BGI_novel_T007304, BGI_novel_T007306 | 2.04E-02 | 3.50E-02 | 4.74E-01 |
| 1127 | LOC113824034 | 1.55 | 1.06 | 1.51 | XM_027376775.1 | 4.53E-02 | 5.70E-02 | 6.32E-01 |
| 1128 | LOC113824295 | 2.96 | 0.24 | 11.07 | XM_027377035.1 | 1.24E-02 | 5.69E-01 | 9.71E-03 |
| 1129 | LOC113824539 | 29.70 | 15.61 | 27.33 | XM_027377278.1, XM_027377279.1 | 1.81E-02 | 1.10E-01 | 2.14E-01 |
| 1130 | LOC113824935 | 5.29 | 2.79 | 4.85 | XM_027377746.1, XM_027377722.1, XM_027377707.1, XM_027377715.1, XM_027377738.1, XM_027377728.1 | 2.36E-04 | 4.55E-02 | 1.25E-01 |
| 1131 | LOC113825364 | 0.73 | 0.33 | 0.51 | XM_027378179.1 | 1.45E-02 | 7.49E-03 | 5.75E-01 |
| 1132 | LOC113826157 | 0.09 | 0.00 | 0.79 | BGI_novel_T000807, XM_027379032.1 | 3.96E-02 | 8.86E-02 | 1.94E-03 |
| 1133 | LOC113826197 | 0.25 | 0.00 | 5.85 | BGI_novel_T008025, XM_027379072.1 | 2.78E-01 | 4.57E-03 | 3.76E-04 |
| 1134 | LOC113827127 | 119.19 | 35.47 | 165.74 | BGI_novel_T008287, BGI_novel_T008288, BGI_novel_T008285, BGI_novel_T008282, BGI_novel_T008284, XM_027380023.1, BGI_novel_T008286 | 1.36E-02 | 9.51E-01 | 1.06E-02 |
| 1135 | LOC113827122 | 27.55 | 2.51 | 20.71 | XM_027380018.1, BGI_novel_T008270, BGI_novel_T008271 | 3.27E-03 | 2.67E-01 | 1.53E-02 |
| 1136 | LOC113827160 | 0.14 | 0.00 | 4.35 | XM_027380061.1 | 6.62E-02 | 3.81E-02 | 2.74E-01 |
| 1137 | LOC113827495 | 0.28 | 0.08 | 7.95 | XM_027380384.1 | 2.64E-01 | 6.62E-03 | 1.68E-03 |
| 1138 | LOC113827633 | 1.99 | 1.84 | 3.69 | XM_027380531.1 | 2.97E-01 | 9.21E-02 | 2.43E-03 |
| 1139 | LOC113827878 | 0.05 | 0.02 | 0.42 | XM_027380820.1, XM_027380821.1 | 7.08E-01 | 2.90E-02 | 2.57E-03 |
| 1140 | LOC113827881 | 1.03 | 0.00 | 0.21 | XM_027380824.1 | 1.40E-04 | 2.47E-02 | 7.17E-02 |
| 1141 | LOC113827879 | 80.55 | 37.55 | 76.04 | XM_027380822.1 | 2.92E-02 | 6.09E-01 | 2.84E-01 |
| 1142 | LOC113828087 | 0.03 | 0.02 | 0.24 | XM_027381028.1 | 6.84E-01 | 3.27E-02 | 7.90E-03 |
| 1143 | LOC113828197 | 1.17 | 0.83 | 0.94 | XM_027381125.1 | 1.84E-01 | 1.59E-02 | 9.45E-01 |
| 1144 | LOC113828344 | 9.39 | 5.04 | 8.71 | XM_027381285.1, BGI_novel_T008623 | 2.04E-02 | 2.29E-01 | 2.58E-01 |
| 1145 | LOC113828819 | 0.01 | 0.00 | 0.09 | XM_027381849.1 | 6.36E-01 | 6.81E-02 | 1.28E-02 |
| 1146 | LOC113829026 | 10.62 | 1.63 | 9.17 | XM_027382103.1 | 3.16E-03 | 7.30E-01 | 6.38E-03 |
| 1147 | LOC113829505 | 0.82 | 0.34 | 0.90 | XM_027382686.1 | 1.49E-02 | 5.69E-01 | 3.81E-02 |
| 1148 | LOC113813165 | 1.22 | 0.80 | 1.23 | XM_027365114.1 | 4.93E-02 | 1.08E-01 | 5.31E-01 |
| 1149 | LOC113829644 | 6.50 | 1.56 | 3.98 | XM_027382853.1, BGI_novel_T009064, BGI_novel_T009065 | 5.87E-03 | 2.74E-02 | 3.07E-01 |
| 1150 | LOC113816596 | 91.96 | 78.57 | 97.90 | XM_027368635.1, XM_027368634.1 | 4.42E-01 | 4.45E-02 | 9.02E-01 |
| 1151 | LOC113830118 | 144.42 | 111.72 | 133.30 | XM_027383312.1 | 3.32E-01 | 5.98E-03 | 9.43E-01 |
| 1152 | LOC113830313 | 6.40 | 4.23 | 6.15 | XM_027383512.1 | 1.16E-04 | 1.64E-02 | 2.39E-01 |
| 1153 | LOC113830606 | 13.82 | 8.88 | 9.61 | XM_027383844.1, XM_027383832.1, XM_027383837.1, BGI_novel_T000990 | 8.30E-02 | 3.36E-02 | 6.79E-01 |
| 1154 | LOC113808108 | 290.54 | 58.35 | 269.91 | XM_027359435.1 | 3.05E-03 | 4.07E-01 | 2.11E-02 |
| 1155 | LOC113800203 | 0.77 | 0.48 | 0.83 | XM_027350928.1 | 1.15E-02 | 3.55E-01 | 4.94E-01 |
| 1156 | LOC113810559 | 2.07 | 0.39 | 9.39 | XM_027362181.1, BGI_novel_T003502, BGI_novel_T003501 | 1.21E-01 | 3.54E-01 | 5.73E-03 |
| 1157 | LOC113823002 | 8.13 | 3.26 | 4.20 | XM_027375573.1 | 2.36E-02 | 4.97E-03 | 8.30E-01 |
| 1158 | BGI_novel_G000189 | 0.21 | 0.00 | 0.70 | BGI_novel_T001236 | 1.40E-01 | 3.03E-01 | 5.52E-03 |
| 1159 | BGI_novel_G000231 | 6.79 | 2.92 | 7.35 | BGI_novel_T001452 | 2.49E-02 | 3.68E-01 | 6.63E-02 |
| 1160 | BGI_novel_G000520 | 28.00 | 18.65 | 20.73 | BGI_novel_T003141 | 1.39E-01 | 1.08E-03 | 8.47E-01 |
| 1161 | BGI_novel_G000625 | 0.78 | 0.16 | 0.44 | BGI_novel_T003785 | 4.42E-03 | 2.17E-01 | 1.50E-01 |
| 1162 | BGI_novel_G000642 | 0.64 | 0.00 | 4.10 | BGI_novel_T003886 | 1.26E-01 | 1.48E-01 | 6.97E-03 |
| 1163 | BGI_novel_G000904 | 8.08 | 3.24 | 9.19 | BGI_novel_T005418 | 1.34E-02 | 6.82E-01 | 4.65E-02 |
| 1164 | BGI_novel_G001092 | 0.23 | 0.12 | 0.84 | BGI_novel_T006545 | 4.12E-01 | 1.19E-01 | 4.84E-02 |
| 1165 | BGI_novel_G001124 | 0.79 | 0.20 | 0.40 | BGI_novel_T006829 | 2.15E-02 | 9.70E-02 | 3.55E-01 |
| 1166 | BGI_novel_G001377 | 0.40 | 0.06 | 1.23 | BGI_novel_T008245 | 2.88E-01 | 4.09E-01 | 1.28E-02 |
| 1167 | BGI_novel_G001451 | 111.44 | 20.26 | 99.54 | BGI_novel_T008861 | 1.34E-03 | 4.37E-01 | 8.87E-03 |
| 1168 | BGI_novel_G001559 | 1.43 | 0.07 | 0.90 | BGI_novel_T009449 | 1.25E-05 | 2.22E-01 | 9.71E-02 |
| 1169 | BGI_novel_G001589 | 0.49 | 0.28 | 0.83 | BGI_novel_T009536 | 2.87E-01 | 8.36E-01 | 3.45E-02 |
